# Engineering Strategies to Suppress Thermal Runaway Propagation in Lithium‐Ion Battery: Mechanisms, Metrics, Materials, and Evaluation Methods

**DOI:** 10.1002/advs.76502

**Published:** 2026-07-13

**Authors:** Jinrong Su, Anna DiFelice, Jayani Mawela, Jianing Gan, Shuai Che, Wonhee Cho, Hanghang Yan, Yaohong Xiao, Xinxin Yao, Xiangwei Guo, Rui Wang, Dingchuan Xue, Hanlong Liu, Solomon Adera, Zhan Chen, Lei Chen

**Affiliations:** ^1^ Department of Mechanical Engineering University of Michigan Dearborn Michigan USA; ^2^ Department of Chemistry University of Michigan Ann Arbor Michigan USA; ^3^ Energy Transport Lab (ETL), Department of Mechanical Engineering University of Michigan Ann Arbor Michigan USA

**Keywords:** battery safety, experimental assessment, flame‐retardant materials, numerical modeling, thermal barriers, thermal runaway propagation

## Abstract

Thermal runaway propagation (TRP) in lithium‐ion battery modules and packs represents a critical safety challenge, as failure of a single cell can rapidly escalate into system‐level hazards. This review provides an engineering‐oriented overview of TRP suppression strategies, focusing on propagation mechanisms, mitigation materials, and evaluation methodologies. First, the distinction between thermal runaway initiation and inter‐cell propagation is clarified, emphasizing the roles of conductive, convective, and radiative heat transfers, gas venting, and combustion in driving cell‐to‐cell failure. The influences of system configuration, propagation modes, and key factors are systematically discussed, along with measurable metrics for TRP risk assessment. Second, materials for TRP suppression are reviewed from a pathway‐oriented functional perspective, including thermal buffering, thermal insulation, flame and gas suppression, and multifunctional composite barriers. Particular attention is paid to how these materials are practically integrated into battery modules and packs to interrupt dominant propagation pathways. Finally, experimental and simulation approaches for TRP assessment are reviewed, highlighting propagation experiments performed under different abuse conditions, as well as physics‐based and data‐driven models. Current challenges and future research directions are outlined. Overall, this review bridges fundamental understanding and engineering practice related to TRP, providing guidance for safer lithium‐ion battery systems with enhanced resistance to TRP.

## Graphical abstract

Thermal runaway propagation can transform a single‐cell failure into a system‐level hazard in lithium‐ion battery packs. This review clarifies how heat transfer, gas venting, combustion, and configuration govern cell‐to‐cell failure, and links measurable metrics, pathway‐oriented suppression materials, and experimental/modeling tools to guide safer module and pack designs with enhanced resistance to cascading failure.

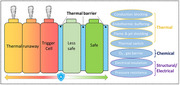

**Abbreviations**
ANN: Artificial neural networks
APP: Ammonium phosphate
ARC: Accelerating rate calorimetry
BH: Bottom surface heating
BTMS: Battery thermal management system
CCH: Calcium chloride hexahydrate
CE: Ceramic
CFD: Computational fluid dynamics
CN: Carbon nitride
CNN: Convolutional neural network
CNT: Carbon nanotubes
DOPO: 9, 10‐dihydro‐9‐oxa‐10‐phosphaphenanthrene‐ 10‐oxide
DSC: Differential scanning calorimetry
DTA: Differential thermal analysis
EDS/EDX: Energy‐dispersive X‐ray spectroscopy
EG: Expanded graphite
EV: Electric vehicles
FE: Finite element
FV: Finite volume
HA: Hexadecyl alcohol
HCCP: Hexachlorocyclotriphosphazene
HPCP: Hexaphenoxycyclotriphosphazene
HRR: Heat release rate
HRR_max_
: Maximum heat release rate
ISC: Internal short circuit
IVP: Isolated venting packaging
LCO: Lithium cobalt oxide
LFP: Lithium iron phosphate
LIB: Lithium‐ion battery
LMO: Lithium manganese oxide
LOI: Limiting oxygen index
LSH: Large surface heating
MCH: Magnesium chloride hexahydrate
MDI: Diphenylmethane diisocyanate
MP: Melamine phosphate
NCA: Nickel cobalt aluminum oxide
NCM: Nickel‐cobalt‐manganese
NCO: Nickel cobalt oxide
NP: Normal packaging
PCFC: Pyrolysis combustion flow calorimetry
PCMs: Phase change materials
PDE: Partial differential equations
PDMS: Polydimethylsiloxane
PEG: Poly(ethylene glycol)
pHRR: Peak heat release rate
PLF140: Oligomeric ethyl ethylene phosphate
PP: Polypropylene
PSF: Phase change material‐silicone rubber foam
py‐FTIR: Pyrolysis–Fourier transform infrared spectroscopy
py‐GC‐MS: Pyrolysis gas chromatography with mass spectrometry
RPUF: Rigid polyurethane foams
SEI: Solid‐electrolyte interphase
SEM: Scanning electron microscope
SH: Side surface heating
SOC: State of charge
SOH: State of health
TCPP: Tris(1‐chloro‐2‐propyl) phosphate​
TGA: Thermogravimetric analysis
TG‐DSC: Thermogravimetry‐differential scanning calorimetry
THR: Total heat release
TPP: Triphenyl phosphate
TR: Thermal runaway
TRN: Thermal resistance networks
TRP: Thermal runaway propagation
TSP: Total smoke production
TSR: Total smoke release
TTI: Time to ignition

## 1 Introduction

Lithium‐ion batteries (LIBs) have become the dominant energy storage technology for electric vehicles (EVs) and large‐scale energy storage systems due to their high energy density and favorable electrochemical performance [1, 2]. However, the increasing integration of large numbers of cells into compact modules and packs has significantly elevated safety concerns [3, 4, 5]. Under abuse conditions, LIBs may undergo thermal runaway (TR), a catastrophic failure mode characterized by uncontrollable heat generation, gas venting, and potential combustion [6, 7].

While the onset of TR in a single cell is hazardous, the situation becomes considerably more critical at the module and pack level, where the failure of one cell can propagate to adjacent cells. This phenomenon, known as thermal runaway propagation (TRP), can trigger cascading failures across multiple cells, leading to large‐scale fires, explosions, and severe system‐level damages [8].

From an intrinsic safety perspective, it is unrealistic to assume that single‐cell failure can be eliminated, given the inherent probability of defects, aging, and extreme operating conditions in LIBs. In contrast, preventing or delaying failure propagation from cell to cell directly limits the scale and consequences of an initial failure [9]. As modern battery systems typically contain hundreds or even thousands of cells, suppressing or delaying thermal propagation has become a central strategy for improving the intrinsic safety of battery systems.

Unlike TR initiation, which is governed primarily by cell‐intrinsic properties and abuse conditions [10], TRP is driven by inter‐cell interactions and system‐level design factors. Heat transfer through cell casings and tabs, vent‐gas release and ignition, flame jet impingement, and mechanical coupling collectively determine whether a localized failure escalates into a multi‐cell event [11, 12, 13]. As a result, TRP is highly sensitive to system configuration, including cell spacing [14, 15], packaging and venting design [16, 17], state of charge (SOC) [15, 18, 19, 20], cell chemistry [19, 21, 22], etc. Understanding these propagation mechanisms is essential for mitigating large‐scale catastrophic TR events.

**FIGURE 1:**
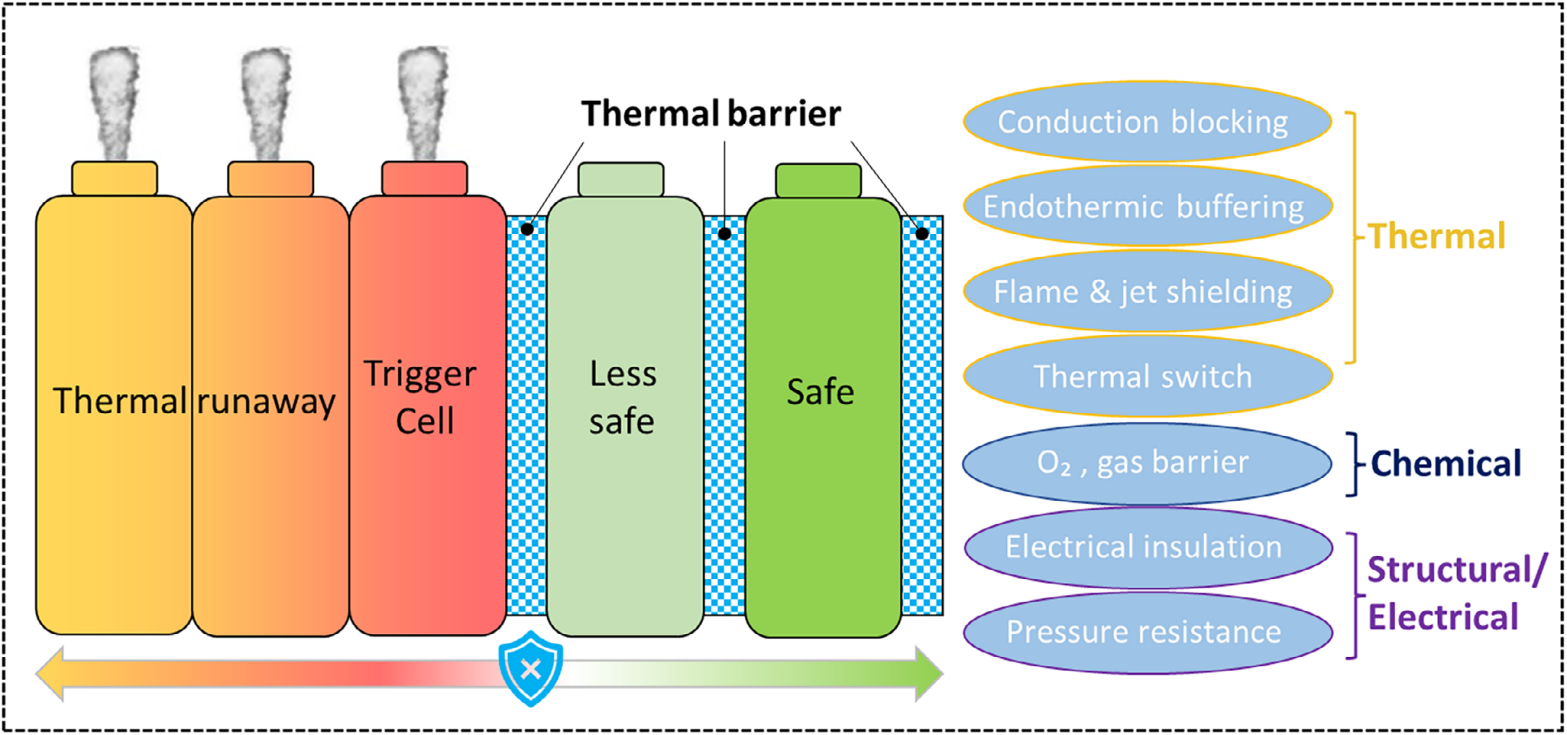
Conceptual schematic of thermal runaway propagation and mitigation mechanisms in battery modules.

As illustrated in Figure 1, thermal barriers provide an effective and largely passive approach for mitigating TRP at the module level [23]. Depending on their material composition, structure, and integration strategy, thermal barriers can suppress propagation through multiple complementary mechanisms [24]. These include thermal functions such as blocking conductive heat transfer, providing endothermic thermal buffering, shielding flames and high‐velocity vent‐gas jets, and exhibiting thermal‐switching behavior under elevated temperatures [23, 25, 26, 27]. Beyond thermal effects, certain barrier designs can also offer chemical isolation by limiting oxygen or flammable gas transport, as well as structural and electrical protection, including electrical insulation and pressure resistance [28, 29, 30]. By interrupting dominant propagation pathways, such barriers can substantially delay or suppress cell‐to‐cell failure.

Despite the significant progress in materials development and battery management systems, limited research has been carried out to systematically address TRP and its mitigation strategies at the battery system level. Although extensive experimental and modeling studies have investigated TR behavior under various abuse conditions, TRP‐related investigations remain fragmented across disparate triggering methods, module configurations, and mitigation concepts. As a result, direct comparison and generalization of propagation behavior are often challenging. Moreover, engineering metrics for evaluating propagation severity and mitigation effectiveness are not consistently defined, and material‐level flame‐retardant strategies are frequently discussed without sufficient linkage to module‐level integration and system‐level performance.

To address these gaps, this review provides an engineering‐oriented overview of recent advances in TRP suppression. First, the fundamental distinction between TR initiation and inter‐cell propagation is clarified, and the dominant physical pathways governing cell‐to‐cell failure are summarized. Key factors influencing TRP behavior at the cell, module, and environmental levels are then discussed. Engineering measurable metrics for TRP risk assessment are reviewed to establish a quantitative basis for evaluating propagation severity and mitigation effectiveness. Subsequently, flame‐retardant and thermal barrier materials are examined from a functional perspective, with emphasis on how they interrupt dominant propagation pathways when integrated into battery modules and packs. Finally, experimental methodologies and computational approaches for TRP assessment are summarized, highlighting their complementary roles in mechanism elucidation and mitigation design. By bridging fundamental understanding and engineering practice, this review aims to provide guidance for the development of safer lithium‐ion battery systems with enhanced resistance to TRP.

## 2 Mechanisms of Thermal Runaway Propagation

TRP is a system‐level escalation process that determines whether an initially localized TR event remains isolated or evolves into a cascading multi‐cell failure. In contrast to TR initiation, which is governed by internal electrochemical reactions and local abuse conditions, TRP is controlled by intercell coupling mechanisms, where heat transfer between adjacent cells and the surrounding module structure. This distinction is critical because propagation directly impacts key hazard outcomes, including the number of failed cells, peak temperature, heat release rate (HRR), and overall event duration. Accordingly, Section 2.1 differentiates initiation and propagation, Section 2.2 maps dominant propagation pathways and governing factors, and Section 2.3 connects these mechanisms to engineering measurable metrics that enable objective risk assessment and mitigation benchmarking.

### 2.1 Differentiating TR and TRP

TR can be initiated by various forms of abuse, including mechanical, electrical, and thermal abuse [1]. Mechanical abuse involves physical deformation or penetration of battery components, often resulting from collisions and crashes or manufacturing defects [31]. It may lead to separator rupture and internal short circuits (ISCs). Electrical abuse encompasses external short circuit [32, 33, 34], overcharging [17, 35], and over‐discharging [36, 37, 38], which can result in joule heating, excess lithium deposition, or dendrite growth [39, 40]. Thermal abuse refers to exposure to extreme temperature conditions. When at high temperatures, external heating or localized hotspots will result in separator melting and cascade exothermic reactions. When in low temperatures, reaction slowdown may induce lithium dendrite growth and trigger ISC [41].

**FIGURE 2:**
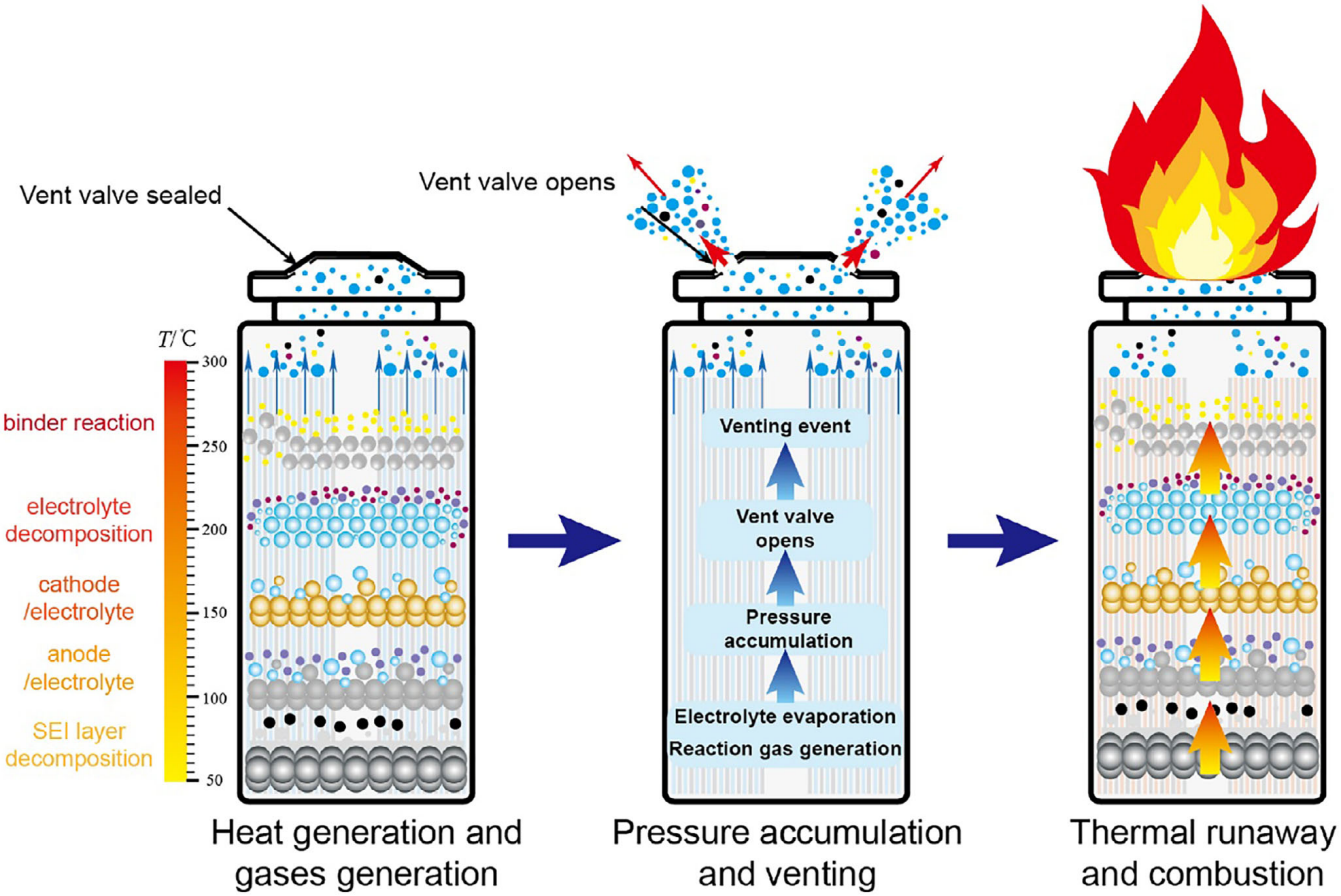
Schematic illustration of thermal runaway process [11]. Copyright 2022, Elsevier.

During the initiation stage of TR, localized failures trigger exothermic reactions within an individual cell. As illustrated in Figure 2, this process begins with heat generation and gas evolution driven by solid electrolyte interphase (SEI) decomposition [42], cathode–electrolyte reactions, electrolyte decomposition, and binder reactions [11]. The accumulation of reaction gases leads to a rapid rise in internal pressure, eventually causing the safety vent to open and release high‐temperature, flammable gases. If ignition occurs, the cell further evolves into TR accompanied by external combustion and intense heat release. The TR process is largely governed by cell‐intrinsic properties, including material chemistry, internal defects, and local abuse severity.

TR initiation is governed by coupled interfacial reactions, gas evolution, and localized failure rather than by a simple sequence of independent temperature‐triggered events [43]. SEI and cathode electrolyte interphase instability can accelerate electrolyte decomposition and gas generation [44], while electrolyte formulation, salt chemistry, additives, SOC, and electrode surface reactivity influence the decomposition pathways and gas species [45, 46]. At higher temperatures, cathode structural degradation and oxygen release may further promote electrolyte oxidation and combustion, especially in high‐energy layered oxide cathodes [47]. Meanwhile, separator shrinkage, melting, mechanical deformation, or ISCs can create localized Joule heating and nonuniform temperature fields [48]. These processes control the transition from mild self‐heating to rapid TR and provide the mechanistic basis for understanding subsequent cell‐to‐cell propagation.

In contrast, TRP is a system‐level phenomenon dominated by inter‐cell interactions rather than the intrinsic stability of an individual cell. Once TR occurs in a trigger cell, the released heat, gases, and flames may be transferred to neighboring cells through conductive heat transfer via casings and tabs, convective heating by vented gases, and radiative heat flux from flames or hot surfaces. These coupling mechanisms determine whether a localized failure remains isolated or escalates into a multi‐cell event. Therefore, while TR reflects the susceptibility of a single cell to failure, propagation governs the escalation and severity of system‐level hazards.

### 2.2 Propagation Pathways and Key Factors Governing TRP

#### 2.2.1 Dominant Heat‐Transfer Pathways and Propagation Modes

The heat released from one cell undergoing TR may be transferred to adjacent cells through three major heat transfer pathways: conduction, convection, and radiation [49]. Conduction is often the dominant pathway in tightly packed modules [22], where direct thermal contact through cell casing, tabs, and structural fixtures enables rapid heat transfer between neighboring cells [50]. Convection arises from the release and transport of high‐temperature vent gases during TR. In semi‐confined spaces, these gases may accumulate, recirculate, and ignite, significantly enhancing convective heat transfer and flame spread [51]. Radiation also plays a significant role, particularly at high temperatures, where intense thermal radiation from a runaway cell can preheat or ignite nearby cells even without direct contact [49].

**FIGURE 3:**
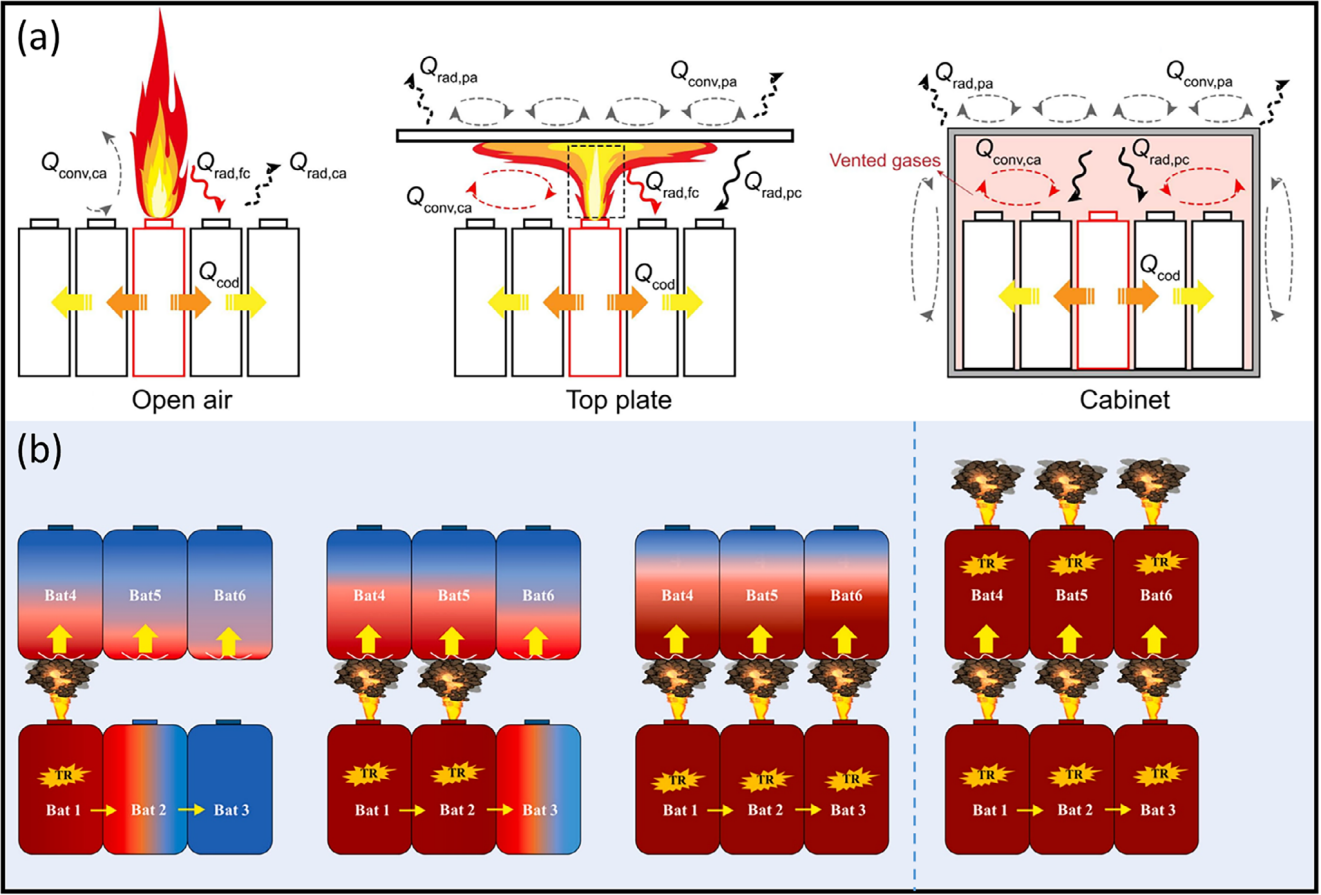
Thermal runaway propagation path (a) Schematics of heat transfer paths for the battery module under various space characteristics [51]. Copyright 2023, Elsevier. (b) Sequential propagation (left panels) and synchronous propagation (right panels) [55]. Copyright 2025, Elsevier.

The practical application scenario of LIBs typically involves three interacting elements: the batteries, the surrounding fluid, and the package enclosure. Accordingly, the dominant heat transfer pathways vary with system configuration, as illustrated in Figure 3a [51]. In open‐air configuration, conduction across cell‐to‐cell interfaces dominates, supplemented by limited convective dissipation to the surroundings. Under a top‐ceiling configuration, jet flames impinge on the ceiling and form a ceiling jet, which deflects horizontally, greatly extending the flame surface and heat transfer area [52]. The top ceiling enhances both radiative feedback and convective heating of neighboring cells. In a cabinet‐like enclosure, oxygen depletion weakens combustion efficiency, but the restricted volume hinders hot‐gas dissipation, causing accumulation of high‐temperature gases. In this case, convective heat transfer from accumulated hot gases becomes the primary driver. These observations highlight that the system configuration determines which heat transfer mode dominates TRP.

In terms of temporal and spatial behavior, two primary propagation modes are commonly reported. When sequential propagation occurs, cells fail one after another along the heat transfer path, with measurable delays ranging from tens of seconds to minutes (Figure 3b) [53]. In contrast, synchronous propagation occurs when multiple cells in stacked configurations undergo TR simultaneously, often driven by strong radiative preheating or flame jetting [15, 54]. Synchronous propagation is associated with a much higher HRR and is therefore more dangerous than the sequential propagation [3]. For example, experiments have demonstrated that radiation from a bottom module can preheat the upper module until it undergoes nearly instantaneous, synchronous failure [15, 54]. In addition, Wang et al. identify a third, disordered propagation, in which an opposite or random propagation order of individual cells within the module is compared to sequential propagation [3]. This mode arises from the complex interplay of multiple factors, including heat transfer across large surface areas, lateral conduction, convective transfer through vent gases, and intrinsic variations among cells, making the propagation sequence irregular and difficult to predict.

#### 2.2.2 Engineering Factors Influencing Propagation Behavior

Propagation behavior is governed by interacting factors across three hierarchical categories: (i) cell‐level intrinsic properties, (ii) module‐level structural design parameters, and (iii) external operating and triggering conditions. These factors collectively determine heat generation intensity, thermal coupling efficiency, and the dominant heat transfer pathways during TRP.

At the cell level, intrinsic properties govern heat release intensity and failure characteristics during TRP, including cathode chemistry, geometry configuration, SOC, and state of health (SOH). For example, high SOC cells release greater stored energy and exhibit more severe propagation behavior [56]. Theiler et al. demonstrated that reducing the SOC of a cell ahead of the propagation path can significantly delay TRP, primarily by lowering the peak temperature and impeding heat transfer [18]. These findings underscore the potential of active SOC control as an effective strategy for TRP mitigation. Differences in cell chemistry also affect behavior. Multiple studies have shown that the TR behavior of LFP and NCM523 modules has a large difference during TRP events [22, 57]. NCM‐based modules often exhibit intense flaming and jetting, while LFP modules are more likely to release high‐speed white smoke without any burning behavior [58]. A review of calorimetric analyses from multiple studies summarizes the TR characteristics of commercial 18650 LIBs, suggesting a general hazard trend of NCA > LCO > NMC > LMO >> LFP [21, 59, 60, 61]. Electrolyte composition is another key cell‐level factor because it directly contacts the cathode, anode, separator, and interfacial layers, thereby affecting thermal stability, gas generation, and side reactions during abuse [62]. For example, Saha et al. demonstrated that different liquid electrolytes in lithium metal batteries lead to distinct TR characteristics through their effects on SEI composition, electrolyte decomposition, and radical‐driven or passivation‐driven reaction pathways [63]. Similar electrolyte–anode interactions have also been reported in sodium‐ion systems in Ranganathan et al.’s work, where anode composition and electrolyte chemistry jointly influence thermo‐electrochemical stability [64]. Conventional carbonate‐based liquid electrolytes, which commonly consist of lithium salts and organic carbonate solvents, can decompose under abuse conditions and generate flammable or toxic gases, thereby intensifying venting, ignition, pressure build‐up, and flame‐assisted propagation. For example, He et al. recently showed that an EC‐less electrolyte can suppress anode–electrolyte exothermic reactions, reduce heat generation, and delay TR in NCM811|Gr pouch cells [65].

In contrast, gel/quasi‐solid, solid‐state, flame‐retardant, and stimulation‐responsive electrolytes have been developed to improve intrinsic safety by reducing leakage, lowering flammability, stabilizing interfaces, or interrupting ion transport under overheating conditions. However, electrolyte safety cannot be evaluated only at the material level. For example, Chen et al. showed that several oxide solid electrolytes can still undergo TR when in contact with metallic Li because oxygen generated or activated from the solid electrolyte can react exothermically with molten Li [66]. Stimulation‐responsive electrolytes have also been proposed to interrupt ion transport or release protective components under thermal, electrical, or chemical stimuli, but their practical effectiveness still requires validation in complete cells and modules.

Coin cells can also serve as laboratory‐scale platforms for probing fundamental thermo‐electrochemical failure mechanisms. Although they are not representative of commercial‐format cells in terms of stored energy, packaging, venting behavior, and heat dissipation pathways, single coin cells are useful for studying material‐level reactivity, interfacial instability, gas generation, and composition‐dependent thermal stability. Stacked coin‐cell configurations can further provide simplified insight into heat accumulation and thermal coupling between adjacent small cells. For example, Zhou et al. performed ARC measurements using four‐coin‐cell stacks to extract thermo–kinetic parameters and then translated these signatures into a pouch‐cell TR model through hierarchical modeling [67]. Nevertheless, such translation should be made cautiously because pouch and cylindrical cells have much larger thermal mass, different casing structures, venting pathways, electrode architectures, and spatial temperature gradients. In addition, heat propagation is faster in pouch cells than in cylindrical cells because of the stronger heat conduction from larger surface areas between pouch cells [13, 57]. As cell dimensions increase, intra‐cell thermal propagation becomes an important intermediate process between local failure initiation and module‐level TRP. For small‐format cells, the trigger cell is often approximated as a lumped heat source in propagation analysis. However, this assumption becomes less accurate for large‐format pouches and prismatic cells, where strong spatial temperature gradients may develop within a single cell. Localized ISCs, nonuniform external heating, or local separator failure can first generate a thermal front that propagates across the electrode stack before the entire cell enters full TR.

At the module level, structural design parameters, such as cell arrangement, electrical connection, tab configuration, inter‐cell spacing, and gap‐filling materials, directly modulate TRP pathways and modes. Inter‐cell spacing and module geometry directly affect the efficiency of thermal coupling; smaller inter‐cell gaps reduce thermal resistance and shorten propagation times, whereas larger spacing or the presence of thermal barriers can delay and suppress propagation [68, 69]. For example, Lopez et al. investigated TRP in a 9P 18650 battery module with inter‐cell spacings of 1, 2, and 4 mm, reporting progressively reduced peak temperatures in adjacent cells as spacing increased [70], because the rate of heat transfer via both radiation and conduction through the tabs decreases with increasing distance. Similarly, Li et al. quantified heat transfer contributions under different heating powers and cell spacings, showing that air conduction dominated at low power and small gaps, while radiation became dominant at higher power and larger cell spacing [71]. These results demonstrate the shifting role of conduction and radiation in TRP under varying power and spacing conditions. Packaging and venting design further influence propagation behavior. Peng et al. conducted comparative experiments on battery modules with normal packaging (NP) and isolated venting packaging (IVP), revealing that NP modules exhibited faster TRP compared to IVP modules in all the tests [16].

Under external conditions, operating environment, triggering method, heating power, TR initiation location, and pressure further shape propagation dynamics and dominant heat transfer modes [19, 24, 72, 73, 74, 75]. Higher heating power enhances energy release rates and strengthens radiative contributions, while confined or poorly ventilated environments intensify heat accumulation and accelerate propagation [72, 73].

The key factors influencing TRP discussed in this section are summarized in Table 1, encompassing cell‐level properties, module‐level design parameters, and external environmental conditions.

**TABLE 1:** Key factors influencing TRP across cell, module level, and external environment.

Scale	Factors	Variables	References
Cell level	Geometric configuration	Coin, cylindrical, prismatic, pouch cell	[22, 67, 70]
	Cathode material	LFP, NMC, LCO, NCO	[21, 22, 60]
	Electrolyte composition	Liquid electrolyte, gel‐state electrolyte, polymer and quasi‐solid electrolytes, inorganic solid‐state electrolyte, stimulation‐responsive electrolyte	[63, 64, 65, 66, 76, 77]
	SOH	0%–100%	[78]
	SOC	0%–100%	[15, 20, 68, 70, 79, 80, 81, 82, 83]
Module‐level	Cell arrangement	Horizontal, vertical, linear, triangular, square, rectangular, rhomboid, and hexagonal	[14, 84, 85, 86]
	Electrical connection	Parallel, series, no connection	[57, 70, 78, 81, 83, 84, 87]
	Tab design	M‐type, S‐type, alternating, straight nickel tab, non‐flat nickel tab	[70, 83]
	Inter‐cell gap	0–20mm	[14, 15, 20, 57, 70, 79, 82, 88, 89]
	Gap‐filling material	Air, flammable PCM, non‐flammable PCM, graphite composite sheet, aerogel felt, ceramic fiber, double‐layer stainless steel, intumescent	[57, 79, 88, 89, 90]
External conditions	External environment	Open air, enclosed chamber, top venting, semi‐confined space, thermostat, wind tunnel	[14, 69, 70, 83, 89, 91]
	Triggering method	Heating rod, thin heating film, resistance wire, heating block, radiation heater, arc, overcharge, mechanical impact, nail penetration, embedded internal short‐circuit devices	[13, 80]
	Heating power	0–2000 W	[68, 73]
	TR initiation location	Center, middle of edge, corner, bottom, battery enclosure outside	[80, 92]
	Pressure	Low‐pressure	[22, 74, 86]

### 2.3 Engineering Measurable Metrics for TRP Risk Assessment

Mechanistic insights into TRP must ultimately be translated into engineering descriptors that enable comparison across module designs, trigger modalities, and mitigation concepts. Because TRP is inherently transient and spatially evolving, qualitative observations (e.g., “propagation occurred” or “flame was suppressed”) are insufficient for design optimization and technology benchmarking. Instead, propagation risk and mitigation performance require a consistent set of measurable metrics that capture (i) the thermal and energetic boundary conditions established by the trigger cell, and (ii) the escalation dynamics that govern cell‐to‐cell failure at module and pack scales. This section consolidates widely used TRP metrics, spanning critical onset temperatures [3, 6, 60, 72, 93], heat release indicators [81, 93], pressure evolution associated with venting and combustion [19, 22, 86, 94, 95], and propagation timing [14, 70, 78, 89, 93].

**FIGURE 4:**
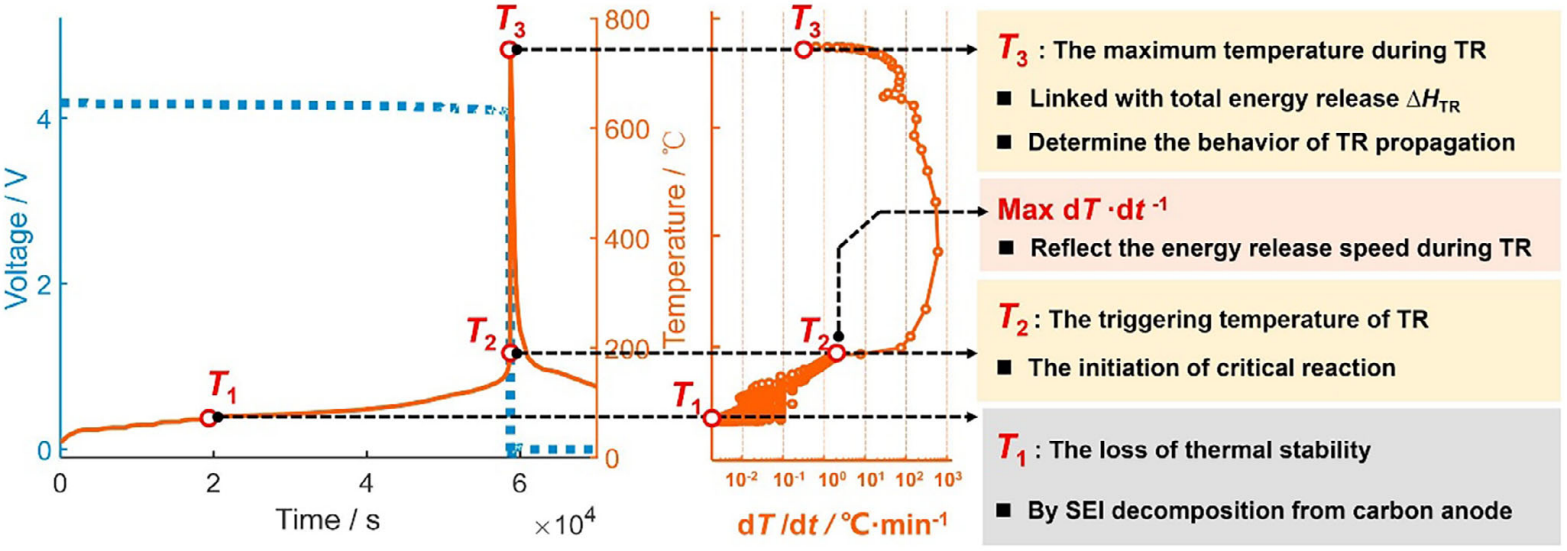
Critical temperatures of battery thermal runaway [96]. Copyright 2019, Elsevier.

At the cell level, characteristic onset temperatures {*T_1_*, *T_2_*, *T_3_*} measured using accelerating rate calorimetry (ARC) are widely used to link experimental observations with the three‐stage TR mechanism (incubation, trigger, runaway) by Feng et al. [96]. As illustrated in Figure 4, *T_1_* represents the onset temperature of detectable self‐heating. *T_2_* is the triggering temperature of battery TR, at which the temperature rise transitions from mild heating to an uncontrollable acceleration. In most studies, a temperature rising rate of 1°C/s is adopted as the criterion for TR onset [72, 93]. However, the definition of *T_2_* is not fully standardized across the experiments, which depends on the experimental configuration, cell format, thermal boundary condition, and diagnostic method. In ARC tests, low self‐heating‐rate thresholds can be useful for identifying the early transition from slow exothermic reactions to accelerated heat generation under near‐adiabatic conditions [97]. For example, Saha et al. performed ARC measurements on a stack of four coin cells and defined *T*_*2*_ as the temperature at which the self‐heating rate exceeds 1°C min^−^^1^ [63]. Similarly, Zhou et al. used ARC tests to evaluate the thermal instability of fast‐charged LFP cylindrical cells and defined *T*_*2*_ as the onset temperature of TR when dT/dt exceeds 1°C min^−^^1^ [98]. He et al. divided the ARC response of 18650 NCM523/graphite cells into multiple stages and used a temperature rise rate exceeding 30°C min^−^^1^ as the criterion for defining the TR trigger temperature [99]. Li et al. further defined TR triggering in overcharged 50 Ah LFP prismatic cells when the average surface temperature reached 133.18°C and the temperature rise rate exceeded 1°C s^−^^1^ (60°C min^−^^1^) [100]. Karmakar et al. used an even higher threshold in ARC tests of 3.25 Ah cylindrical 18650 Li‐ion cells, defining the beginning of TR when the temperature rise rate exceeds 100°C min^−^^1^ [101]. Externally heated cells, large‐format cells, and module‐level propagation tests may exhibit stronger thermal gradients, delayed sensor responses, and more rapid temperature escalation, leading to the use of higher temperature‐rise‐rate criteria. As a result, direct comparison of *T_2_* values across different studies should be performed with caution. For engineering benchmarking, *T_2_* should be reported together with the corresponding dT/dt threshold, heating protocol, sensor location, cell format, SOC, and thermal boundary conditions. *T_3_* (or *T_max_*) denotes the maximum temperature reached during TR. Maximum heat release rate (HRR_max_) serves as a critical indicator of runaway severity. The HRR, which is reflected by dT·dt^−1^, defined as the intensity of the battery TR and varies depending on the cell chemistry. When the local HRR exceeds the cooling or heat absorption capacity of the surrounding module structure, TR is likely to propagate to adjacent cells.

Beyond temperature‐ and rate‐based indicators, temporal descriptors provide complementary information on TR initiation kinetics and TRP mitigation effectiveness. Metrics such as time to self‐heating onset, time to TR onset, time to venting, time to ignition, propagation delay, and total propagation time capture different stages of the failure process [102]. For single‐cell abuse tests, time to self‐heating onset and time to TR onset reflect the resistance of the cell chemistry and internal components to accelerated exothermic reactions. Time to venting and time to ignition are closely associated with gas generation, pressure accumulation, and combustion hazard. For module‐level tests, propagation delay and total propagation time directly reflect the effectiveness of thermal barriers, spacing design, cooling structures, and venting pathways in delaying or interrupting cell‐to‐cell propagation.

Pressure rise associated with gas venting is an important risk indicator. Zhao et al. employed the EV‐ARC with a sealed canister to study the pressure of the battery during the TR process [103]. Liu et al. conducted in situ internal pressure measurements on large‐format 300 Ah LiFePO_4_ cells and showed that pressure evolution can be divided into distinct stages associated with inert gas expansion, electrolyte vaporization, and chemically generated gases [104]. Their results indicated that safety venting occurs when the internal pressure exceeds a critical threshold, accompanied by the release of high‐temperature and flammable gases. Importantly, pressure not only reflects the severity of internal reactions but also directly influences external hazard modes. Hofmann et al. demonstrated that reducing ambient pressure during TR can significantly suppress cell rupture and explosion by facilitating early gas release and enhanced endothermic cooling through electrolyte evaporation [105]. Their results indicate that pressure conditions critically affect gas‐phase behavior, ignition likelihood, and the violence of failure events.

Recent studies have further proposed refined or composite safety metrics that integrate multiple descriptors, such as onset time, TR onset temperature, maximum temperature rise rate, T_max_, HRR, gas generation, and pressure evolution. Compared with single indicators, these integrated metrics can provide a more comprehensive assessment of thermal stability, reaction kinetics, and hazard severity, which is particularly useful for comparing cathode chemistries, electrolyte formulations, and mitigation strategies. However, composite metrics also have limitations because their values may depend on testing protocols, heating conditions, sensor locations, and the availability of complete temperature, calorimetry, pressure, and gas data. Therefore, these metrics are useful for multidimensional safety ranking, but their cross‐study applicability still requires standardized testing methods and transparent reporting of the underlying parameters.

The ARC‐derived parameters do not directly describe propagation; instead, they define the initial thermal and energetic boundary conditions that govern whether propagation becomes possible at the module level.

At the module and pack level, propagation‐related metrics include the number of failed cells, total propagation time, propagation speed (cells∙s^−^^1^), and valve opening intervals, which collectively characterize the spatial and temporal evolution of TRP. For example, Huang et al. experimentally investigated TRP in large‐format NCM and LFP battery modules and quantified key propagation parameters, including inter‐cell propagation time, propagation speed, maximum temperature, mass loss, and gas release behavior. Their results showed that NCM modules exhibit significantly shorter propagation intervals, higher propagation speeds, and more severe thermal and combustion behavior than LFP modules.

These parameters are particularly valuable for comparing mitigation strategies across different module designs and triggering conditions. Table 2 summarizes the engineering metrics commonly reported in the literature for TRP assessment.

**TABLE 2:** Summary of key metrics reported for TRP experimental analysis.

Parameter	Definition	Reference
Adjacent cell maximum temperature (K)	Peak temperature reached by an adjacent cell during propagation	[60]
Temperature rise rate	The rate of temperature increase in an adjacent cell before TR	[81]
Mass loss (g)	The difference between the initial mass of the battery cell and the mass of the cell remnants after TR	[3, 45, 60]
Mass loss rate (%)	Rate of mass loss during TR	[60]
Heat release rate (HRR kW)	Instantaneous heat release rate of a battery fire	[69]
Number of failed cells	The number of cells that are triggered to TR	[69]
Valve opening interval (s)	Time interval between safety valve openings of adjacent cells	[81, 93]
Propagation time (s)	The interval between the TR trigger times of adjacent batteries	[72, 93]
Total propagation time (s)	The time interval from the moment TR is triggered in the first cell to the moment the last cell in the module experiences TR	[69]
Propagation speed (cell/s)	The number of cells that are triggered to TR divided by the time of TRP	[69, 83]

By translating complex failure phenomena into measurable quantities, these metrics provide a quantitative foundation for evaluating mitigation effectiveness. Accordingly, effective flame‐retardant and thermal management strategies must be designed to reduce peak temperatures, lower HRR, delay propagation onset, and limit the number of failed cells. The next section introduces the key functional mechanisms by which materials achieve these objectives and discusses their practical integration into battery modules and packs.

## 3 Materials and Architectures for TRP Mitigation

Flame‐retardant and thermal‐mitigation materials suppress TRP through multiple, often complementary, mechanisms operating across different temperature and spatial scales. At the most fundamental level, TRP mitigation strategies can be categorized into four primary mechanisms: (i) heat absorption and thermal buffering, which reduce peak temperature rise through endothermic phase transitions or decomposition; (ii) thermal insulation and low‐conductivity barriers, which restrict intercell heat transfer and maintain temperature gradients; (iii) gas suppression and flame inhibition, which dilute combustible gases, quench reactive radicals, and limit oxygen diffusion; and (iv) multifunctional and system‐level integration strategies, which combine thermal, chemical, and mechanical effects within compact architectures. These mechanisms do not act independently. Instead, effective TRP mitigation typically relies on synergistic coupling between heat absorption, heat‐flow restriction, gas‐phase suppression, and structural stabilization. The following sections systematically review these mechanisms, their representative material systems, and their engineering integration in battery modules and packs.

### 3.1 Heat Absorption and Thermal Buffering

Heat absorption is one of the most direct ways to mitigate TRP [106, 107]. These materials work through endothermic processes; instead of only blocking heat, they absorb a large amount of energy through melting, vaporization, or chemical decomposition (that is, utilizing the waste heat to break chemical bonds). This can reduce the peak temperature, slow down heat transfer to nearby cells, and delay TRP by lowering the temperature of the vent gas [108, 109, 110]. Heat‐absorbing materials can also act as a heat sink during normal battery operation, such as during discharge, and release the stored heat later when the temperature drops during charging, when the battery is in a colder environment, or when the battery is not operating at the peak load [111].

Phase change materials (PCMs) mainly work through the absorption of latent heat that is stored in the solid–liquid phase transition. Storing waste heat via phase change in PCMs has many advantages, including a nearly isothermal temperature that matches the melting/solidification temperature [106, 107, 108, 111, 112, 113, 114]. Moreover, using PCM in battery thermal management can significantly reduce cell‐to‐cell and cell‐to‐pack temperature variability by temporarily storing the waste thermal energy via solid–liquid melting. For a well‐designed system, employing PCM in battery thermal management systems can be used to set the upper temperature limit at the melting point of the PCM. By breaking the direct bond/link between the heat source and heat sink to disallow fast propagation of changes in the boundary condition at the heat source to the external cooling, using PCM creates a buffer zone between the heat source and heat sink. It is well suited for transient thermal management to reduce temperature fluctuation caused by nonuniform heating. During TRP, the battery temperature increases until it reaches the PCM phase change. The PCM then absorbs heat, which slows down the temperature rise. Compared with sensible heat storage, latent heat storage can provide a smaller temperature swing, since phase change occurs at constant temperature (isothermal process). Due to the order of magnitude higher energy storage capacity (compared to sensible heating), using PCM can substantially reduce temperature variability, hence indirectly reducing the probability of TR. Overall, using PCM in battery thermal management is an emerging trend that has huge potential in improving the cooling efficiency of battery cells and battery packs. For EV battery applications, an ideal PCM should have a melting point around 30–60°C, high latent heat per unit mass, a smaller volumetric expansion coefficient, good cycling stability, faster response for heating and cooling (that is, smaller time constant), and good safety that does not increase the risk of fire [111, 112].

Paraffin wax is the most used PCM. It was first introduced to reduce the need for complex active cooling systems that circulate air or liquid coolants. As a passive heat sink, paraffin wax has good thermal properties and a relatively low phase transition temperature, plus it is also low cost [113, 114]. During TRP, the liquid–solid PCM can keep the temperature lower while it melts, and after it becomes liquid, the low thermal conductivity can slow heat transfer from the runaway cell.

A key limitation of many PCMs is their low thermal conductivity, which limits the heat transfer rate [112, 114, 115, 116, 117]. Although the total heat capacity is high enough for TR, heat cannot spread through the whole PCM volume due to the low thermal diffusivity, which can cause local hot spots near the runaway location. Under extreme conditions, after the latent heat is fully used, PCM becomes a liability as it suffocates the heat transfer pathway due to its low thermal conductivity. Under extreme situations, this could even contribute to faster heating locally, with the potential to contain the heat spread. Another concern is the flammability of paraffin wax. If the PCM burns, combustion adds extra heat to the battery pack and can worsen the event [109, 118].

To improve PCM performance, thermal enhancement materials are often combined with PCMs [116]. This can be done by mixing high‐conductivity fillers (for example, carbon nanotubes, graphene powder, silicon nanoparticles) into the PCM or by infiltrating the PCM into a designed heat‐transfer structure, such as metal matrices(for example, copper or aluminum foam structures) or ordered carbon fiber [114]. PCMs are also combined with thermal insulation barriers such as aerogels, where the PCM helps control temperature during normal operation and early stages of abuse, while the aerogel serves as an effective insulator during TRP [118].

Among all enhancement materials, the graphite matrix is one of the most promising options because it offers high thermal conductivity, which does not decrease with the impregnation of the PCM, and is relatively simple to form [117]. Another approach is adding carbon fibers into paraffin wax, which can significantly increase PCM thermal conductivity. According to the study, both the fiber length and loading affect performance, and fibers longer than about 8 mm may not distribute uniformly, which reduces the benefit [114].

Metal matrices and metal nanoparticles can also enhance heat transfer in composite PCM structures. Experiments showed that a metal‐matrix/PCM composite reduced the maximum temperature difference between the battery surface and the PCM composite by up to 70%. Among nano‐enhanced PCMs, composites containing silver nanoparticles showed better thermal performance than those using other metal nanoparticles [113].

Silica can be used to absorb paraffin wax to reduce leakage and can also help limit thermal‐induced volumetric expansion concerns in graphite‐based structures [119, 120]. To address nonuniform filler distribution, carbon‐nanotube‐based three‐dimensional networks have been developed, which can reduce PCM leakage and improve structural uniformity [115].​

**FIGURE 5:**
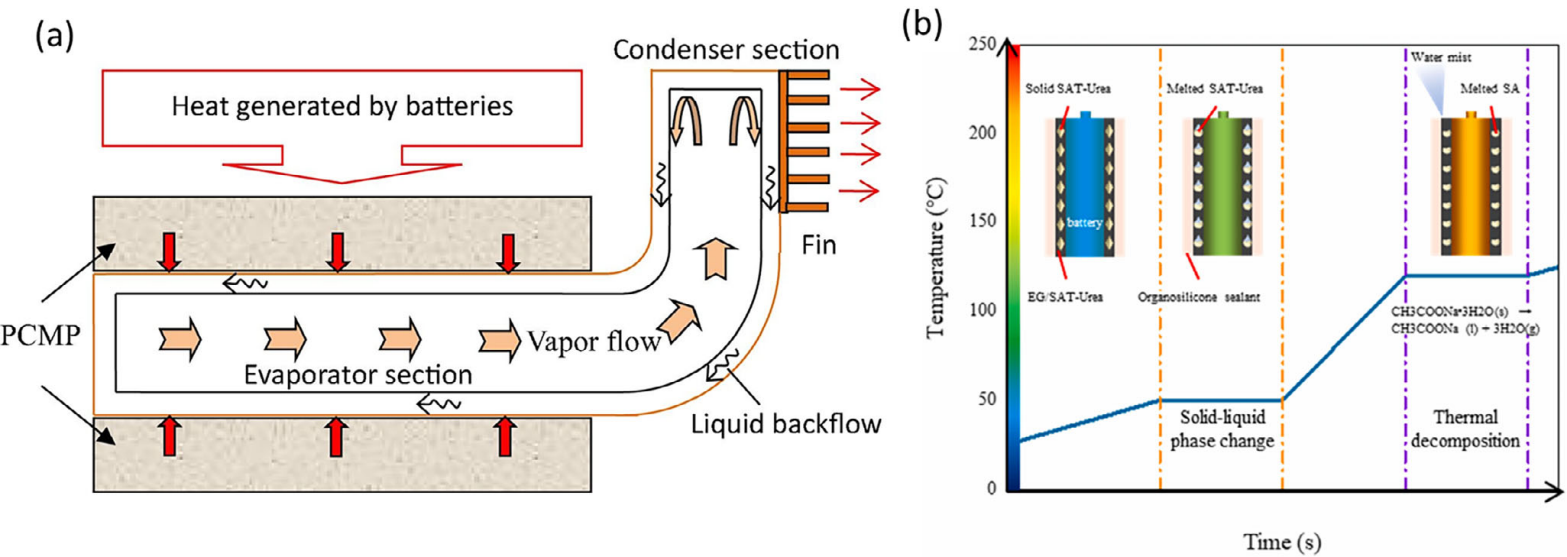
(a) Schematic diagram of heat transfer in a PCM system cooperating with a heat pipe [123]. Copyright 2017, Elsevier. (b) Design principle of the SAT‐urea/EG inorganic salt hydrate PCM system, showing latent heat storage and thermochemical storage occur at different temperatures [126]. Copyright 2023, Elsevier.

Interestingly, heat pipes have also been explored as an assist for PCM‐based thermal management [121]. In the concept shown in Figure 5a, the PCM absorbs heat while the heat pipe improves heat spreading and heat removal to the surrounding environment, which can reduce peak battery temperature and improve temperature uniformity. However, this approach is mainly designed for thermal management during normal operation and may not be optimized for extreme TR events. Since heat pipes can rapidly move heat from a small hot spot to the rest of the system, they could potentially change how heat builds up during TRP, so careful design is needed. It could also be an opportunity for future designs to adopt heat pipes into useful heat dissipation tools for the uniform temperature distribution through the whole PCM when TRP occurs.

Hydrates can also be used as thermochemical material (TCM) for energy storage [122, 123, 124, 125]. They are crystalline compounds consisting of water molecules and guest compounds. The thermal properties of hydrates depend on the guest compounds. Compared with organic waxes, they can offer better stability, and their phase transition temperatures can be close to room temperature. For example, trimethylol ethane hydrate shows a phase transition at 29.6°C and a latent heat of 190.1 kJ/kg, which is higher than typical paraffin‐based systems [125]. The hydrate also shows better thermal stability than pure paraffin wax. This makes hydrates a potential option for low‐temperature battery thermal management.

Another example, a sodium acetate trihydrate–urea/expanded graphite composite uses a two‐stage heat absorption mechanism [124], as shown in Figure 5b. In the first stage, it undergoes a solid–liquid transition at 50.3°C and provides a latent heat storage capacity of 181 kJ/kg. This helps keep battery temperature below about 50°C during normal operation, which can reduce performance loss. In the second stage, at a higher temperature, the material decomposes at 114.0°C and releases steam, with a thermochemical storage density of 567.3 kJ/kg, which can provide stronger protection during rare but severe TRP events. This two‐stage design is attractive because batteries operate normally most of the time but still need strong protection for extreme cases.​

However, the effectiveness of PCM‐based barriers is limited by their applicable temperature and heat‐flux ranges. PCMs are most effective before or during the early stage of thermal abuse, when latent heat absorption can delay temperature rise. Once the latent heat is exhausted, or when low thermal conductivity limits heat spreading within the material, local hot spots may still develop near the trigger cell. Material‐specific limitations should also be considered. For example, paraffin‐based PCMs may introduce flammability concerns, whereas hydrated‐salt PCMs can suffer from phase separation or supercooling, limiting their reliability as TRP barriers. Phase separation is a major challenge for salt‐hydrate PCMs. During repeated cycles, the hydrate can separate into a salt‐rich phase and water, which gradually reduces heat storage performance. Some studies have started to develop methods to reduce supercooling and phase separation [122]. Meanwhile, cost is still a concern because inorganic PCMs are often more expensive than paraffin wax. In the future, continued progress in formulation design and scalable processing is expected to make hydrate PCMs more stable, safer, and more practical for wider battery applications.

### 3.2 Thermal Insulation and Low Conductivity Barriers

Thermal insulation–based barriers can be considered as one of the most widely implemented strategies for mitigating TRP in LIB modules and packs [26, 27, 107, 126, 127]. The fundamental objective of this approach is to block or significantly slow down the heat transfer between adjacent cells by introducing materials with inherently low thermal conductivity. By maintaining a large temperature difference across cell interfaces, insulating materials delay the transfer of thermal energy from a cell undergoing TR, thereby reducing the likelihood that neighboring cells reach their TR onset temperature within a short time frame [27, 128, 129, 130]. This mechanism is particularly important in tightly packed battery packs, where intercell spacing is minimal and conductive heat transfer dominates the early stages of propagation.

Unlike PCMs or active cooling strategies, thermal insulation does not rely on heat absorption or heat removal; instead, it presents a barrier for heat transfer by increasing the conduction resistance. As a result, the effectiveness of insulation‐based barriers is primarily governed by intrinsic thermal conductivity, microstructural porosity, thermal stability at elevated temperatures, and the ability to maintain physical integrity during abuse conditions [25, 90, 107, 126]. Aerogels, mica‐ and ceramic‐based sheets, and polymer composites with porous or layered structures are typical representatives of this category and have been extensively investigated across different cell formats and module configurations [25, 27, 127, 131, 132, 133, 134, 135, 136].

Aerogels are among the most extensively studied insulating materials due to their exceptionally low thermal conductivity, which arises from their highly porous, nanoscale network structures [132, 137, 138, 139, 140, 141]. Silica aerogels and nanofiber‐based aerogel composites exhibit thermal conductivities that are orders of magnitude lower than those of dense polymers or ceramics [131, 132, 142]. Experimental studies demonstrate that aerogel interlayers placed between adjacent cells can significantly delay TRP, lower peak temperatures in neighboring cells, and, in some cases, completely suppress propagation under specific test conditions. Thickness‐dependent investigations further show that increasing aerogel thickness enhances propagation suppression by increasing the thermal resistance between cells [25, 75, 132, 133, 137, 138, 141]. However, these improvements are accompanied by increased volume and mass penalties, which directly impact module‐level energy density and packaging efficiency.

**FIGURE 6:**
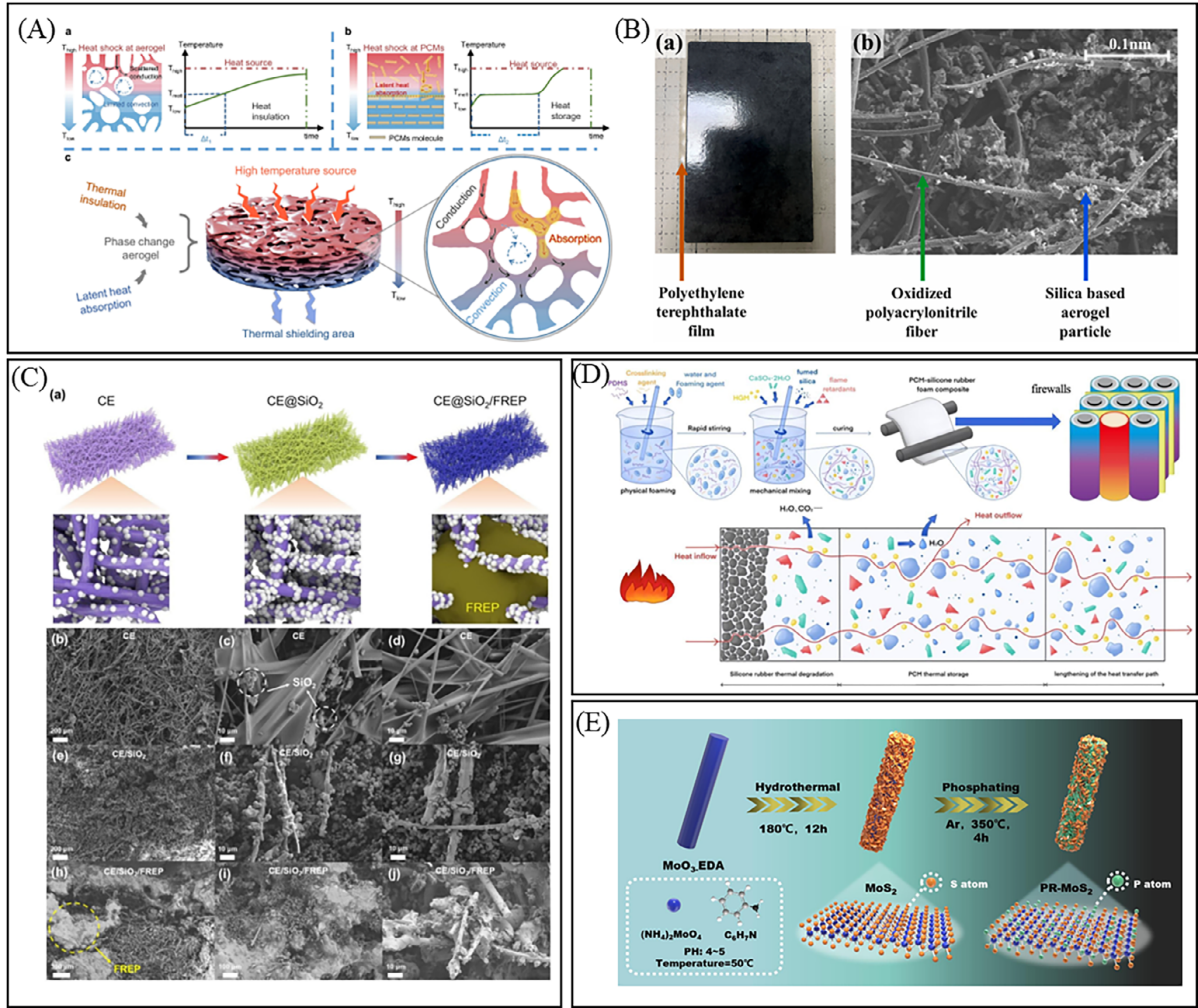
(A) An aerogel‐based PCM composite design as an effective insulation barrier under thermal shock conditions [143]. Copyright 2024, Springer Nature. (B) Optical and SEM images of a sample of nanofiber aerogel [133]. Copyright 2024, Elsevier. (C) Ceramic‐based thermal‐insulator synthesis and characterization [75]. Copyright 2025, Elsevier. (D) Illustration of PSF preparation and the schematics of the mechanisms of flame retardancy and thermal insulation of PSF [134]. Copyright 2025, Elsevier. (E) Preparation scheme of phosphorus‐doped MoS_2_ (PR‐MoS_2_) for epoxy‐based thermal‐insulation barrier [24]. Copyright 2024, Elsevier.

Despite their favorable thermal performance, conventional aerogels suffer from mechanical fragility and brittleness, which limit their direct applicability in practical battery systems [134, 137]. Under TR conditions, mechanical deformation, gas release, and pressure buildup can compromise barrier integrity. To address these limitations, several studies have explored reinforced aerogels and nanofiber aerogel composites that combine low thermal conductivity with improved mechanical robustness [75, 133, 141]. Aerogels can limit the effects of thermal shock by suppressing heat transfer through ultralow thermal conductivity (Figure 6A), while phase change materials stabilize temperature via latent heat absorption. PCM–aerogel composites combine both mechanisms, offering enhanced protection through simultaneous thermal insulation and heat buffering [143]. Moreover, Figure 6B shows an SEM image of a nanofiber aerogel, composed of silica‐based aerogel particles combined with oxidized polyacrylonitrile fibers and coated with a polyester film. The structural stability of this system provides effective thermal insulation in compact, pressure‐applied battery modules [133]. These hybrid aerogel systems retain insulation performance while better tolerating mechanical stress and thermal shock, making them more suitable for large‐format prismatic cells and densely packed modules.

Therefore, aerogel‐based barriers are most applicable to scenarios where conductive heat transfer between adjacent cells dominates, and moderate‐to‐high‐temperature insulation is required. However, their performance can be limited under strong mechanical compression, vibration, or cell swelling, which may damage the porous structure and increase effective thermal conductivity. Under severe TR conditions involving flame impingement, hot particle ejection, or high‐velocity gas jets, aerogel barriers may suffer from structural degradation or insufficient resistance to non‐conductive heat‐transfer pathways. These factors define the practical failure boundaries of aerogel‐based barriers in module‐level TRP mitigation.

Mica‐ and ceramic‐based insulating materials represent another important class of low thermal conductivity barriers. These materials offer excellent thermal stability, non‐flammability, and resistance to high‐temperature degradation, allowing them to maintain structural integrity well beyond typical TR temperatures [23, 27, 75, 135]. Figure 6C shows the synthesis of an improved type of ceramic (CE)‐based material. Initially, CE material has porous ceramic fibers with loosely attached SiO_2_ particles, limiting adhesion and insulation. However, after immersion in silica gel, followed by freeze‐drying, the system converts from a gel to an aerogel, which leads to filling gaps and binding the particles, enhancing mechanical integrity and thermal insulation. Remaining detachment is addressed by infusing the composite with flame‐retardant resin, which coats the fibers and reinforces the ceramic skeleton [75]. Commercial mica sheets and ceramic fiber boards have been evaluated as intercell barriers and shown to effectively suppress heat transfer and flame propagation. Their ability to act as both thermal and flame barriers makes them particularly attractive for severe abuse scenarios. However, the relatively high density, stiffness, and brittleness of ceramic and mica materials can introduce challenges related to mechanical compatibility and mass penalties, especially in applications where compactness and weight reduction are critical.

Polymer‐based insulating barriers provide a more integration‐friendly alternative and are therefore widely investigated for practical battery applications. Porous polymer composites, flame‐retardant polypropylene interlayers, and polymer foams combine low thermal conductivity with electrical insulation and mechanical compliance. Several experimental studies demonstrate that flame‐retardant polypropylene and silicone‐based barriers can significantly reduce intercell heat flux and delay TRP in cylindrical and prismatic cell assemblies [29, 126, 134, 144, 145]. Within the silicone‐based category, silicone elastomers and polydimethylsiloxane (PDMS)‐based composites are increasingly explored as insulating barriers due to their thermal stability, mechanical compliance, and tunable low thermal conductivity. Pristine PDMS exhibits inherently low thermal conductivity, which can be further tailored through composite design using fillers such as graphene, metal oxides, expanded graphite, or phase change materials [146, 147, 148, 149]. Reinforced PDMS systems maintain effective thermal resistance while improving interfacial conformity and mechanical integrity under thermal and mechanical stress. PDMS‐based phase change composites can additionally buffer transient heat during thermal abuse, contributing to delayed heat transfer to adjacent cells. Silicone rubber foams and fluorosilicone blends further reduce effective thermal conductivity through controlled porosity and expansion behavior, strengthening their role as passive insulation layers for suppressing intercell heat flux during TRP. Figure 6D illustrates the preparation of a phase change material‐silicone rubber foam (PSF) composite with multi‐scale fillers designed for thermal management. The optimized PSF material exhibits low thermal conductivity, high heat absorption, and mechanical reinforcement, providing excellent thermal insulation that delays heat transfer, flame retardancy, and structural robustness [134]. These materials are particularly attractive because they are compatible with conventional battery manufacturing processes and can be readily integrated into existing module designs.

Epoxy‐based insulating composites further expand the design space for polymer‐based barriers. When reinforced with inorganic fillers, layered materials, or flame‐retardant additives, epoxy composites can form thermally stable char layers during TR [24, 30, 75, 150]. In Figure 6E, the preparation of phosphorus‐doped MoS_2_ nanowires (PR‐MoS_2_) is shown. Incorporated into epoxy resin, this nanowire composite increases char yield, reduces peak heat release and CO production rates, and a 3 mm interlayer between pouch cells has the capability to fully suppress TR while keeping the surviving battery temperatures below 100 °C and preserving structural and electrochemical integrity [24]. This char formation not only limits heat transfer but also suppresses flame spread and reduces oxygen diffusion toward neighboring cells. Such multifunctional insulation behavior enhances safety performance while maintaining mechanical strength under high‐temperature conditions. However, epoxy systems generally exhibit higher thermal conductivity than aerogels, highlighting the need for careful formulation and microstructural control [75, 151].

Comparative studies across different insulating material classes consistently identify low thermal conductivity as the dominant parameter governing propagation suppression. Insulation‐based barriers are effective at maintaining large temperature gradients between cells, slowing down heat propagation even under severe abuse conditions. At the same time, multiple studies emphasize compromises associated with excessive insulation. Highly insulating barriers can trap heat within the initiating cell, potentially leading to local hotspots, internal pressure, and mechanical stress. These effects may influence failure severity or exhaust gas release behavior, particularly in confined module designs [29, 134, 144].

Consequently, the engineering integration of thermal insulation barriers requires careful optimization of material selection, thickness, placement, and coverage area. Insulation performance must be balanced against practical constraints such as module volume, mass, manufacturability, and compatibility with venting pathways. Rather than maximizing insulation in isolation, effective designs tailor insulation properties to specific cell formats, module architectures, and expected abuse scenarios. When appropriately optimized, thermal insulation and low‐conductivity barriers provide a robust and scalable foundation for passive TRP mitigation.

### 3.3 Gas Suppression and Dilution

Flame retardants utilizing gas suppression prevent reactive, high‐temperature gases from spreading and reaching other flammable components of the cell. Both gas and condensed‐phase reactions contribute to diluting or quenching flammable gas products and forming a char layer, respectively. In the beginning, halogenated compounds were used in flame‐retardant materials, quenching the reactive gaseous species formed during a fire, but these compounds proved to be toxic and persistent, leading to their prohibition [159, 160]. Later, gas‐phase active phosphorus, nitrogen, and metal‐based flame‐retardants are more common.

Phosphorus and nitrogen‐containing compounds are common in new formulations as they will produce beneficial gas‐phase products when degraded [161, 162]. Li et al. scaffold the flame retardants hexaphenoxycyclotriphosphazene (HPCP) and triphenyl phosphate (TPP) in paraffin, expanded graphite (EG), and a PEG‐based coordination polymer, creating a new PCM capable of both absorbing heat and extinguishing fires during TR [163]. During TR, the HPCP, TPP, and expanded graphite synergistically form a dense char layer that prevents heat and oxygen from igniting neighboring cells. HPCP and TPP generate phosphoric acid, PO, and PO_2_* that neutralize gas‐phase radicals, and the HPCP releases NH_3_ and N_2_, incombustible gases that dilute flammable gases and restrict oxygen consumption. Containing both nitrogen and phosphorus, phosphazene is crucial in the formulation, and samples without it could not fully self‐extinguish. Phosphorus‐ and nitrogen‐based flame retardants suppress and dilute reactive gas release, creating effective flame‐retardant materials.

Other fillers also work through gas dilution, such as hydrated metals and metal hydroxides. When heated, hydrated metals and metal hydroxides release inflammable gas that will dilute both oxygen and reactive or toxic gas products [164, 165].In addition, remaining metals may catalyze the formation of a char layer. Similarly, aerogels will release water when heated, but unlike metal‐based fillers, may instead trap the inflammable gases within holes, creating pockets that can isolate heat and increase the flame‐retardancy similarly to intumescent materials [166].

In some materials, the char layer and condensed‐phase reactions can prohibit the release of gaseous products. In Lv et al.’s work, a phosphorus‐free, boron‐based, nickel flame retardant was mixed into epoxy resin [167]. As the material was heated, a strong surface char layer was created which prevented the escape of gases and led to the formation of large holes within the body of the material. These holes delayed the release of bulk volatile products while also becoming insulation layers. Here, the condensed‐phase reactions and formation of a strong char could have a substantial effect on smoke and gas release.

In summary, effective flame‐retardants work both in the solid and gas phases to limit the reactivity of gas products, buffer the spread of heat, and suppress smoke release. Phosphorus species create radical‐quenching byproducts that prevent fire from spreading. Both nitrogen and phosphorus compounds also degrade into nonreactive and nonflammable gases, diluting oxygen and reactive gases. Hydrated metal salts, metal hydroxides, and aerogels utilize water to suppress the flame. Finally, the formed char layers prohibit combustion by limiting oxygen and, in some cases, by trapping gas within the material.

### 3.4 Multifunctional Composites and Integrated Design

Multifunctional composite barriers represent an important evolution in TRP mitigation, addressing the limitations of single‐function insulation or heat‐absorbing materials. Some examples are presented in Table 3. By combining multiple mitigation mechanisms—such as heat absorption, thermal insulation, flame suppression, and mechanical reinforcement—within a single material system, multifunctional composites achieve synergistic effects that enhance overall safety performance. This integrated approach has gained increasing attention as battery modules are required to be more compact, lightweight, and energy dense while meeting increasingly demanding safety requirements [23, 29, 90, 127, 135, 136, 157, 168].

**TABLE 3:** Examples of TRP‐suppressing materials, highlighting the use case and mechanism of suppression.

Material (example)	Functional role	Key mechanism	References
Paraffin‐based PCM	Heat absorption buffer	Latent heat storage during phase transition	[107, 111, 152, 153]
Flame‐retardant PCM composite (paraffin + additives)	Heat absorption + flame suppression	Latent heat absorption + flame inhibition	[131, 136, 154, 155, 156]
CNT‐enhanced inorganic PCM	Heat spreading + absorption	Enhanced thermal conductivity improves heat dissipation	[142, 156]
PCM–silicone rubber foam composite	Thermal management + insulation	Heat absorption + elastic thermal barrier	[134]
Silica aerogel sheet	Thermal insulation barrier	Ultralow thermal conductivity	[25, 137, 139]
Nanofiber aerogel composite	Structural thermal barrier	Porous insulation+ mechanical robustness	[133, 157]
Polyimide–silica composite aerogel	Insulation + flame resistance	Thermal insulation + fire retardance	[132, 141]
Ceramic fiber–reinforced aerogel	Mechanical & thermal barrier	Structural integrity + heat shielding	[75]
Aerogel + PCM multilayer barrier	Hybrid thermal barrier	Insulation + latent heat absorption	[138, 158]
Flame‐retardant polypropylene (PP) barrier	Inter‐cell thermal barrier	Low thermal conductivity + flame resistance	[144]
Thermo–mechanical–chemical composite barrier	Multifunctional protection layer	Thermal insulation + mechanical constraint + chemical suppression	[29, 135]
Phosphorus‐doped MoS_2_/epoxy composite	Fire‐resistant barrier	Char formation + thermal shielding	[24]
Heat‐triggered fire‐extinguishing polymer composite	Active suppression layer	Heat‐activated release of extinguishing agents	[150]
Interstitial heat‐absorbing barriers	Cell‐to‐cell protection	Heat absorption between cells	[26, 90]
Integrated liquid cooling + aerogel system	Passive–active hybrid cooling	Active heat removal + passive insulation	[127, 140]

A key motivation for multifunctional barrier development is the growing gap between safety requirements and packaging constraints. Battery designers are under constant pressure from customers and system integrators to reduce module size and weight while increasing energy and power density. As a result, stacking multiple single‐function layers is often impractical. Multifunctional composites offer a pathway to achieve safety functions in fewer layers, reducing volume and mass penalties while maintaining or improving their effectiveness [28, 128, 169, 170, 171, 172].

One widely explored multifunctional strategy involves combining PCM with thermally insulating frameworks [27, 107, 111, 128, 142, 152, 154, 157, 169, 173]. In PCM–aerogel composites, latent heat absorption from the PCM delays the temperature rise during TR, while the aerogel framework blocks conductive heat transfer between cells. Experimental studies show that these hybrid systems outperform either PCMs or aerogels alone, providing longer propagation delay times and lower peak temperatures in adjacent cells [141, 143, 153, 158, 174, 175]. The aerogel structure also helps confine molten PCM, reducing leakage and maintaining barrier integrity under high‐temperature conditions.

Similar synergistic behavior has been reported for PCM composites embedded in polymer foams or elastomeric matrices. In these systems, the polymer framework provides mechanical support, electrical insulation, and flame retardancy, while the PCM contributes thermal buffering through phase transition. Flexible PCM‐based composites have demonstrated improved compatibility with realistic battery geometries and better tolerance to mechanical deformation compared to rigid PCM blocks. These properties are particularly advantageous for large‐format prismatic cells and cell‐to‐pack architectures [23, 28, 127, 142, 154, 155, 168].

Polymer–ceramic hybrid composites form another important class of multifunctional barriers. In these materials, ceramic components contribute thermal stability and intrinsic flame resistance, while polymer matrices offer flexibility, toughness, and ease of processing [75, 135]. Epoxy and silicone‐based composites reinforced with ceramic fillers or layered inorganic materials have been shown to suppress TRP through a combination of reduced heat transfer, char formation, and mechanical integrity at elevated temperatures [24, 30, 134]. Phosphorus‐containing additives further enhance flame suppression by promoting the formation of radical‐quenching species and stable char layers that limit oxygen diffusion and combustible gas ignition [24, 155].

Nanostructured multifunctional composites expand the design space by enabling precise control over thermal, mechanical, and chemical properties. Aerogel–polymer hybrids and nanofiber‐reinforced architectures provide low thermal conductivity with resistance to mechanical collapse, addressing a key limitation of conventional aerogels [133, 135, 142, 156]. These materials maintain structural integrity under combined thermal and mechanical stress, which is critical in densely packed modules where barrier deformation can compromise safety performance.

Beyond material‐level design, multifunctional concepts increasingly extend to module‐ and system‐level integration strategies. Compartmentalization approaches physically isolated groups of cells with multifunctional barriers, limiting the spatial extent of TR events. Venting control strategies direct hot gases and flames away from neighboring cells and critical components, reducing the likelihood of secondary ignition. Flame‐free propagation concepts further minimize fire risk by preventing direct flame impingement on adjacent cells. Studies combining multifunctional materials with optimized venting and compartmentalization demonstrate substantially improved mitigation performance compared with material‐only solutions [176, 177].

Integrated design strategies also benefit from modeling and optimization efforts that account for thermal, mechanical, and geometric constraints simultaneously. Model‐based studies show that barrier placement, thickness distribution, and interstitial coverage can be optimized to maximize propagation suppression while minimizing penalties in mass and volume. These results highlight that multifunctional barriers are most effective when treated as part of an integrated system design rather than as isolated material inserts.

Overall, multifunctional composites and integrated design strategies represent a shift toward practical and scalable TRP mitigation. By combining heat absorption, thermal insulation, flame suppression, and mechanical stability within a single material or structural element, these approaches align safety performance with real‐world engineering limitations. As battery systems continue to evolve toward higher energy density and tighter integration, multifunctional barriers are likely to play an increasingly central role in achieving robust TRP mitigation without sacrificing compactness or performance.

## 4 Experimental and Computational Approaches

The effectiveness of TRP mitigation strategies ultimately depends on whether they can shift propagation outcomes under realistic boundary conditions. Therefore, TRP research requires a closed loop between module‐level comparative experiments, material‐level characterization, and predictive modeling. Experimental platforms provide application‐relevant validation by intentionally triggering failure in a designated cell and observing whether and how propagation evolves across neighbors, whereas modeling frameworks enable mechanistic interpretation and efficient exploration of design spaces at reduced risk and cost.

Importantly, cross‐study comparability remains a key barrier: propagation outcomes are highly sensitive to trigger modality, module geometry, venting/combustion environment, and measurement definitions, making “suppression” or “delay” claims difficult to benchmark without consistent reporting. This section therefore summarizes experimental and computational approaches from a design‐oriented perspective: (i) how propagation tests are conceptually designed and compared, (ii) how mitigation effectiveness is quantified in controlled module configurations, and (iii) how physics‐based, hybrid, and data‐driven models provide predictive guidance for integration strategies and material screening prior to high‐risk large‐scale testing. Section 4.1 reviews module‐level TRP experiments (how propagation tests are designed, triggered, and compared), Section 4.2 outlines material‐level thermal/flammability characterization that informs mitigation design and model inputs, and Section 4.3 reviews modeling approaches.

### 4.1 Experimental Methods for Propagation and Mitigation Assessment

Experimental methods provide the primary means for evaluating TRP and validating mitigation strategies at the module level. Through controlled abuse scenarios, TRP experiments enable direct observation of propagation behavior and allow mitigation performance to be assessed via comparative testing.

In the following subsections, representative experimental methodologies are summarized from three complementary perspectives: (i) the conceptual design principles underlying TRP propagation, (ii) triggering strategies used to initiate propagation, and (iii) experimental approaches for assessing the effectiveness of propagation mitigation.

#### 4.1.1 Conceptual Design for TRP Experiments

Rather than assessing the abuse tolerance or failure threshold of an individual cell, TRP evaluation experiments are designed to intentionally trigger failure in a designated trigger cell and examine whether, how, and how rapidly the failure propagates to neighboring cells within a module. This experimental paradigm explicitly targets cell‐to‐cell interactions, including interfacial heat transfer [49], vent‐gas ignition, flame jet impingement [178], and pressure‐driven mechanical effects, which collectively govern escalation from localized failure to module‐level hazards.

From a mitigation assessment perspective, the core design principle of such experiments is comparative evaluation. For example, Lopez et al. systematically compared baseline modules with modified configurations incorporating increased inter‐cell spacing, radiant barriers, and intumescent materials, enabling direct assessment of how module design and interstitial protection alter propagation behavior [70]. By introducing flame‐retardant materials, thermal barriers, or structural modifications into otherwise identical module configurations, TRP tests enable direct comparison of propagation behavior with and without mitigation. For example, Liang et al. adopted a strictly controlled comparative TRP experimental design, in which identical prismatic battery modules were subjected to the same nail‐penetration triggering conditions, while only the inter‐cell thermal environment was varied. By comparing a baseline air‐cooled module with otherwise identical modules incorporating immersion cooling, the study quantified mitigation effectiveness using propagation‐oriented metrics such as propagation interval, peak temperature, and temperature rise rate [179]. Such comparisons allow researchers to isolate the effectiveness of suppression strategies under controlled conditions, independent of intrinsic cell‐to‐cell variability. Consequently, TRP experiments serve as a critical bridge between material‐level characterization and system‐level safety evaluation.

#### 4.1.2 Triggering Strategies for Initiating TRP

A prerequisite requirement of TRP experiments is the reproducible initiation of TR in the trigger cell. Triggering strategies are commonly categorized into thermal, electrical, and mechanical abuses [180].

**FIGURE 7:**
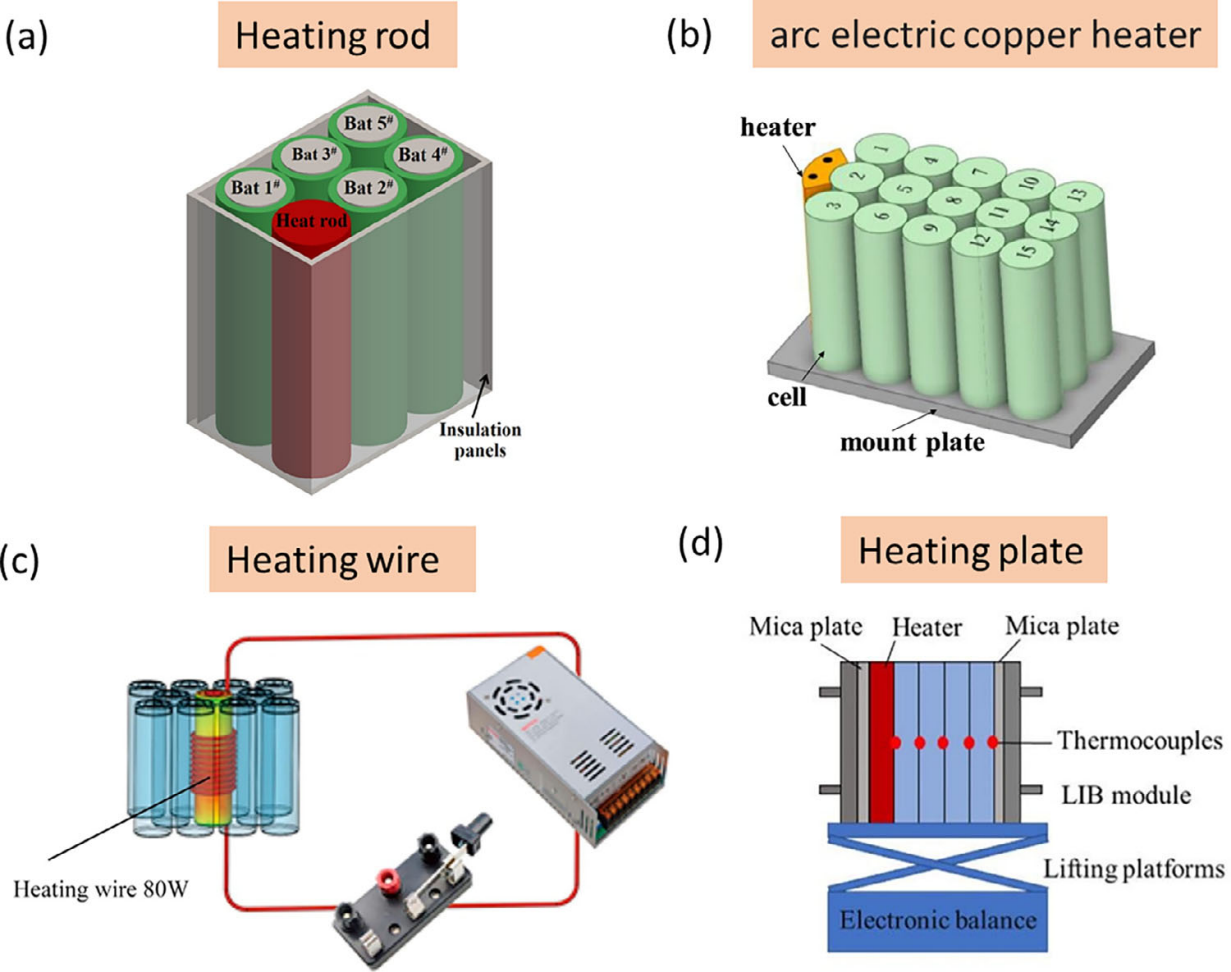
Schematic illustrations of typical thermal‐abuse triggering methods employed in TRP experiments: (a) heating rod [22], Copyright 2022, Elsevier. (b) ARC electric copper heater [69], Copyright 2025, Elsevier. (c) heating wire [90], Copyright 2019, Elsevier. And (d) heating plate [78]. Copyright 2024, Elsevier.

Among the various approaches, thermal abuse is the most widely adopted strategy for TRP studies due to its high controllability and reproducibility. External heating is considered to be the best repeatable triggering method [72], in which a single trigger cell is externally heated until TR occurs, and the subsequent propagation to neighboring cells is monitored. As illustrated in Figure 7, several external heating strategies have been developed to impose controlled thermal loading on the trigger cell, including heating rods [22], heating wires [90], ARC electric copper heaters [69], and heating plates [78]. Huang et al. compared the three most representative heating positions, namely the large surface heating (LSH), side surface heating (SH), and bottom surface heating (BH), with the same heat flux density [181]. Their results show that TR under large surface heating exhibited the highest smoke volume, jet velocity, and longest duration of jet. Although these methods differ in heating configuration and boundary conditions, they share a common objective: to reproducibly initiate TR in a single cell while minimizing uncertainties associated with electrochemical or mechanical failure modes. Consequently, thermal‐abuse‐based TRP tests provide a robust experimental platform for isolating cell‐to‐cell heat transfer and evaluating the effectiveness of thermal barriers and flame‐retardant materials in suppressing propagation.

**FIGURE 8:**
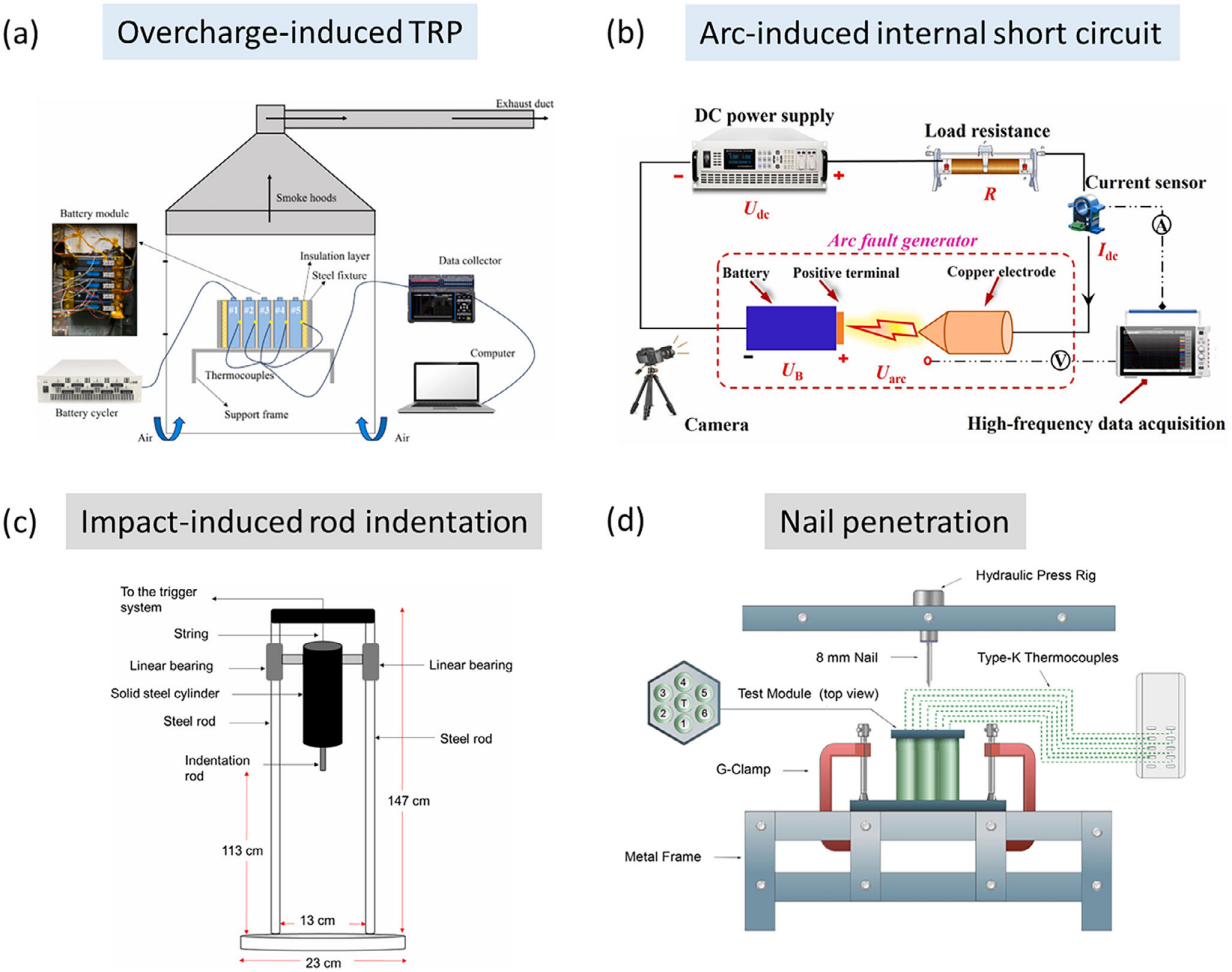
Electrical and mechanical abuse methods used to trigger TRP. (a) Overcharging [183], Copyright 2024, Elsevier. (b) Arc‐induced internal short circuit [187], Copyright 2024, Elsevier. (c) Impact‐induced rod indentation [188], Copyright 2022, Elsevier. and (d) nail penetration [189]. Copyright 2025, Elsevier.

Electrical and mechanical abuse methods provide complementary triggering pathways that are more representative of accident‐like or operational failure scenarios. Electrical abuse triggers TRP through electrochemical instability and internal heat generation [8]. Among these methods, overcharge‐induced TRP has been extensively studied, as excessive charging accelerates lithium plating [39, 40], electrolyte decomposition, and gas release [182], ultimately leading to TR and propagation to adjacent cells [27, 82, 178]. As shown in Figure 8a, continuous charging beyond the rated voltage was applied to trigger TRP in Li et al.’s work [183]. Huang et al. experimentally compared TRP induced by overcharging and overheating and quantified the normalized critical energy required to trigger TR under each condition [184]. They found that overcharge‐induced TR is more severe and catastrophic due to the higher heat release, more combustible gases, and mass loss. Mallarapu et al. compared TRP triggered by embedded internal short‐circuit devices and thin‐film heating, and found that the former resulted in more limited propagation in multi‐cell configurations [185].

More recently, arc‐induced ISCs have been identified as a critical but previously underexplored TRP trigger, particularly relevant to battery energy storage systems [186]. As illustrated in Figure 8b, sustained series arcs at loose terminals or degraded connections generate intense localized heating at the electrode–tab interface in Xu et al.’s work, which can melt separators and initiate ISCs [187]. Compared with conventional thermal abuse, arc‐induced triggering introduces coupled electrical–thermal effects and can result in earlier ignition and more violent propagation behavior.

Mechanical abuse tests simulate accident‐related failure scenarios such as collision, crush, or penetration. Impact‐induced rod indentation [188] and nail penetration tests [189] are employed to directly create ISCs by physically damaging the electrode–separator structure. As shown in Figure 8c, Md Said et al. demonstrated that impact‐induced rod indentation can trigger violent TR accompanied by intense flame jets and ejection of hot battery contents, while limited contact area and air gaps can significantly hinder heat transfer to adjacent cells, thereby suppressing propagation [188]. Lai et al. compared TRP behaviors under heating, nail penetration, and overcharge triggering at the module level and revealed that trigger mode strongly influences the early‐stage propagation time and severity, whereas these differences tend to diminish as propagation progresses [12]. They further reported that the nail penetration leads to the shortest trigger time, while the heating trigger offers better experimental controllability and lower cost, making it more suitable for systematic TRP testing.

#### 4.1.3 Mitigation Effectiveness Assessment

Mitigation assessment in TRP experiments is typically achieved through controlled comparisons rather than introducing new evaluation quantities. Baseline modules are contrasted with architectures incorporating thermal barriers, intercell fillers, increased spacing, or composite flame‐retardant structures, and mitigation is reflected in whether propagation is suppressed, whether onset in adjacent cells is delayed, and whether severity indicators such as peak temperature and flame/venting intensity are reduced. Notably, relative performance can be more engineering‐relevant than absolute suppression: substantial propagation delay or severity attenuation may satisfy safety requirements by enabling intervention or preventing large‐scale escalation, even if complete propagation prevention is not achieved.

**FIGURE 9:**
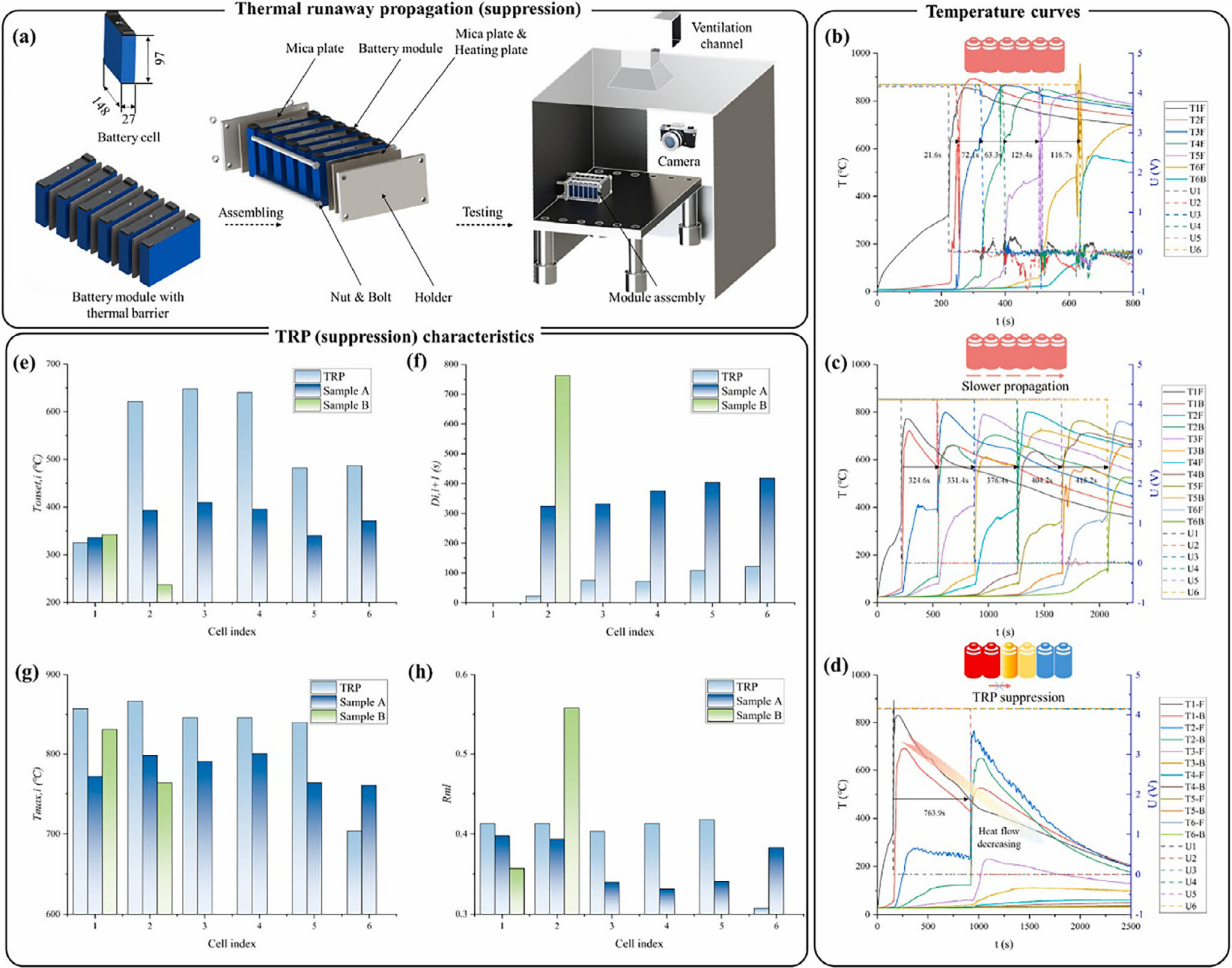
Experimental assessment of TRP mitigation at the module level in Chen et al.’s work [23]. Copyright 2025, Elsevier. (a) TRP suppression experimental setup; (b–d) Temperature–voltage profiles (TRP, barrier A/B based TRP suppression); (e, f) TRP parameter comparison in three tests, including thermal runaway triggering temperature, TRP interval, maximum temperature, and maximum temperature time.

Liu et al. investigated overcharge‐triggered TRP in battery modules with and without thermal insulation layers [190]. By comparing temperature and voltage responses under a 3C overcharge condition, they showed that aerogel‐ and fiber‐based insulation materials effectively blocked heat transfer and maintained the surface temperature of the adjacent cell below 200°C, thereby suppressing propagation. In such experiments, mitigation effectiveness is reflected in qualitative and semi‐quantitative outcomes, including whether propagation occurs at all, whether the onset of failure in adjacent cells is delayed, and whether the intensity of propagation—manifested by peak temperatures, flame jets, or gas release—is reduced. As shown in Figure 9, Chen et al. designed a module‐level TRP suppression experiment incorporating composite thermal barriers between adjacent cells (Figure 9a) [23]. The temperature–voltage profiles under baseline and barrier‐assisted conditions (Figure 9b–d) reveal pronounced delays in the onset of TR in neighboring cells and a clear reduction in peak temperatures during propagation. Correspondingly, the extracted TRP parameters (Figure 9e,f) show a substantial extension of the propagation interval and attenuation of propagation severity when thermal barriers are introduced. Their experiments demonstrated that the thermal barrier effectively suppressed TRP, extending the propagation interval between the first two triggered cells to 762.8 s. These observations provide direct evidence of how specific design interventions alter propagation pathways, such as conductive heat transfer or vent‐gas ignition.

Importantly, experimental assessment of mitigation effectiveness emphasizes relative performance rather than absolute thresholds. Even when complete suppression of propagation is not achieved, substantial delays or reductions in propagation severity may satisfy engineering safety requirements by enabling emergency intervention or preventing large‐scale escalation. In this sense, TRP experiments provide a practical and application‐oriented framework for validating mitigation concepts prior to system‐level deployment.

#### 4.1.4 System‐Level Integration and Trade‐Offs in TRP Mitigation

TRP mitigation should be considered as a system‐level integration problem rather than only a material‐selection or cooling‐design problem. In practical battery modules, the effectiveness of a mitigation strategy depends not only on the intrinsic properties of thermal barriers, flame‐retardant materials, PCMs, or cooling components, but also on how these elements are integrated with cell spacing, module structure, venting pathways, cooling plates, electrical connections, and mechanical constraints. These design elements collectively determine the dominant heat‐transfer routes, gas/flame exposure, contact thermal resistance, pressure relief behavior, and propagation sequence during TRP. Therefore, system‐level TRP mitigation requires coordinated design of cooling pathways, inter‐cell barriers, venting direction, cell arrangement, and packaging constraints.

During the normal charging and discharging process, a battery thermal management system (BTMS) is essential for maintaining cells within a suitable operating temperature window and limiting temperature nonuniformity across the battery pack. Previous studies commonly suggest that lLIBs should operate near 15°C–35°C, while the temperature difference among cells is generally maintained below ≈5°C [191, 192]. Most conventional BTMS designs are developed for normal operating conditions, such as fast charging, high‐power discharging, and transient drive cycles, where the primary design objective is to efficiently extract heat from battery cells and minimize cell‐to‐cell, cell‐to‐pack, and pack‐to‐pack temperature variability in a battery module [193].

Recently, increasing attention has been given to full‐lifecycle battery thermal safety management, which considers TR caused by normal operation and accidents [128]. Although BTMS and TRP‐mitigation systems both involve controlling rapid thermal propagation, their operational requirements and focus are fundamentally different. Under normal operation, the heat generation rate is typically in the order of watts to tens of watts per cell, depending on cell chemistry, capacity, C‐rate, SOC, temperature, and cooling conditions [194, 195]. In contrast, during TR, the heat‐release rate can increase by several orders of magnitude to a few kilowatts, depending on chemistry, geometry, SOC, abuse trigger, and calorimetry method [196]. This large difference in thermal energy release rate explains why a BTMS that is effective during normal cycling may not be sufficient to prevent TRP under abuse conditions.

To slow down TRP within a battery module, the BTMS should be designed to address both normal operating conditions and abuse scenarios. Various BTMS strategies have been proposed to suppress TRP in LIBs, including both active cooling methods and passive protection approaches. Among active methods, liquid cooling has been widely investigated because it can support both normal battery cooling and thermal safety control. Cold plates, mini‐channel liquid cooling, liquid spray cooling, and immersion cooling can remove heat directly or indirectly from the cell surface. In a recent study by Yang and co‐workers, different BTMS configurations for suppressing TRP were compared, and showed that liquid cooling can effectively slow down thermal propagation when a sufficiently large coolant flow rate is used [197]. In a related numerical study, Fu et al. used mini‐channel liquid cooling and found that channel width and coolant velocity strongly influence TRP mitigation [198]. These studies indicate that active cooling can contribute to thermal safety management. However, they also highlight an important system‐level challenge: the coolant flow rate required during TRP may be much higher than that required during normal operation. This may increase the demand for pump capacity, radiator size, control complexity, and emergency‐response reliability.

Spray cooling and water‐mist cooling have also been investigated for TR suppression [199, 200]. However, their practical implementation requires careful consideration of electrical insulation, coolant compatibility, flame‐induced buoyancy, gas release/ejection, and whether droplets or spray can effectively reach the heat release zone. Immersion cooling can provide strong thermal coupling between cells and coolant, but its practical adoption in EVs is still limited by coolant cost, long‐term chemical stability, toxicity and disposal concerns, sealing reliability, and serviceability [128].

Because of these limitations, passive thermal‐safety components are often needed in addition to active cooling. Thermal insulation‐based barriers are one of the most widely used passive strategies for mitigating TRP in lithium‐ion battery packs. These barriers can provide effective thermal insulation, heat absorption, flame shielding, and gas or particle blocking. Their basic mechanism is to reduce the heat transferred from the failed cell to neighboring cells, thereby delaying or preventing secondary TR. From this standpoint, low thermal conductivity material is beneficial during TRP because it limits heat propagation, but it can increase thermal resistance during normal cycling if the barrier interrupts the main heat rejection mechanism. Therefore, the key engineering question is not simply whether insulation should be used, but where and how it should be used and integrated with the system‐level BTMS.

From the perspective of normal BTMS operation, inter‐cell thermal insulation does not necessarily degrade cooling performance if the primary heat‐rejection pathway from each cell to the cooling infrastructure is preserved. In most single‐phase liquid‐cooled cold plate designs, heat is mainly removed through direct or indirect contact between the cell surface and the cold plate [193]. Therefore, when the insulation layer is placed mainly between adjacent cells rather than between the cell and the cold plate, its influence on the normal liquid cooling mechanism and the resulting heat rejection rate is limited. This design concept has been demonstrated in several active–passive hybrid configurations. Yang et al. experimentally showed that aerogel insulation alone delayed TRP but could not completely stop it, and that liquid cooling alone provided only a limited delay. In contrast, the combined use of aerogel insulation and a liquid‐cooling plate successfully prevented TRP in the tested module [201]. Rui et al. further investigated the synergistic effect of inter‐cell insulation and bottom liquid cooling, demonstrating that thermal insulation between neighboring cells and heat dissipation through the cooling plate can work together to mitigate TRP [201]. Liu et al. also proposed a hybrid design using mini‐channel cold plates and insulation layers to inhibit TRP in a battery pack [202]. These studies indicate that active cooling and passive barriers can be complementary rather than contradictory: the cooling plate removes heat during normal operation, whereas the inter‐cell barrier suppresses abnormal cell‐to‐cell heat transfer during TR.

Phase change materials (PCMs) provide another representative example of this trade‐off. PCMs are used as passive BTMS materials because they can absorb heat via solid–liquid melting. However, conventional organic PCMs, such as paraffin, generally suffer from low thermal conductivity and flammability. Therefore, recent studies have focused on composite PCMs with enhanced thermal conductivity, improved flame retardancy, and higher thermal stability. For example, inorganic hydrated‐salt PCMs can absorb heat and release water at elevated temperatures, making them attractive for both thermal management and TRP mitigation [123, 142]. Metal‐based PCMs or multi‐temperature microencapsulated PCMs have also been proposed to provide thermal regulation during normal operation and additional heat absorption during abuse conditions [203]. These materials suggest a promising direction for thermally adaptive safety design, although their cost, mass, volume, manufacturability, and long‐term durability still require further evaluation.

Overall, the trade‐off between BTMS performance and safety requirements should be addressed through integrated design rather than treating cooling and insulation as separate entities. The two (cooling effectiveness and safety considerations) are intertwined in a battery thermal management system design. Hybrid cooling‐barrier designs, thermally adaptive materials, and optimized layouts can maintain efficient heat removal during normal operation while limiting heat transfer, flame spread, and particle ejection during TR [108, 204, 205, 206]. Nevertheless, the effect of insulation remains design‐dependent. In air‐cooled modules, or in layouts where lateral heat spreading between cells contributes significantly to temperature uniformity, inter‐cell insulation may increase thermal resistance and worsen temperature nonuniformity. By contrast, in many cold plate single‐phase liquid cooling systems, properly positioned inter‐cell insulation is less likely to compromise normal BTMS performance because the dominant heat transfer path is from the cell to the cold plate, rather than from cell to cell. Future work should therefore optimize cooling efficiency, TRP suppression, mass, volume, cost, manufacturability, and long‐term reliability at the cell, module, and pack levels.

### 4.2 Material‐Level Thermal and Flammability Characterization

After TRP experiments are performed, characterizing the heat‐resistant, thermal barrier materials alone can yield (1) the temperatures of phase transitions or degradations, (2) the time‐dependent absorption or release of energy, (3) the identity of potential degradation products, (4) the approximate flammability, and (5) mechanical suitability. Through this analysis, the mechanism behind favorable thermal behavior and the real‐world applicability are explored. Metrics such as latent heat for PCMs, thermal conductivity for barrier materials, and the limiting oxygen index for flame‐retardant materials can be used to compare chemically different materials.

1. Estimating temperatures of phase transitions and degradations during TRP, including the assessment of thermal stability. Thermogravimetric analysis (TGA) and differential scanning calorimetry (DSC) are the most common ways to analyze the response of a material to increasing heat.
2. Diagnosing the spread, absorption, and release of heat from the material. Thermal conductivity and diffusivity characterize how heat can spread through the material. Cone calorimetry is commonly used to calculate the HRR over time.
3. Characterizing degradation products in smoke or char layers. Cone calorimeters also detect smoke release. Pyrolysis–Fourier transform infrared spectroscopy (py‐FTIR or TG‐FTIR) and pyrolysis gas chromatography with mass spectrometry (py‐GC‐MS) can separate and detect species within gaseous byproducts. Analysis of the char after burning is also common using scanning electron microscopy (SEM), FTIR, or Raman spectroscopy.
4. Testing the flammability of the material. Two standard tests are used to quantify flammability, the UL‐94 and the limiting oxygen index (LOI).
5. Examining the mechanical properties. Tensile, flexural, and compressive stress–strain curves may determine how cell separators behave mechanically during TRP experiments. Dynamic mechanical analysis probes how mechanical properties will change with increased use or temperature.

Being easily accessible and quick, TGA, DSC, thermal conductivity, cone calorimetry, LOI, UL‐94, and compressibility measurements are commonly used to find the best flame‐retardant candidate material to run on a TRP platform. Some values for different types of flame‐retardant materials are in Table 4.

**TABLE 4:** Examples of data from thermal conductivity measurements, TGA, DSC, cone calorimetry, PCFC, LOI, and UL‐94 testing for common flame‐retardant materials and fillers. From TGA using N_2_, the temperatures at max mass loss rates (T_max_) and the ending %residue are presented. DSC is used to calculate the latent heat of PCMs. With cone calorimetry and PCFC, the peak heat release rate (pHRR) and total heat release (THR) are calculated. APP = ammonium polyphosphate, CN = carbon nitride, CNT = carbon nanotubes, DOPO = 9, 10‐dihydro‐9‐oxa‐10‐phosphaphenanthrene‐ 10‐oxide, EG = expanded graphite, HA = hexadecyl alcohol, HCCP = hexachlorocyclotriphosphazene, MCH = magnesium chloride hexahydrate, MDI = diphenylmethane diisocyanate, MP = melamine phosphate, NCM = recycled nickel–cobalt–manganese cathode material, PLF140 = oligomeric ethyl ethylene phosphate, TCPP = tris(1‐chloro‐2‐propyl) phosphate.

Primary Material	Fillers (wt%)	Thermal conductivity (Wm^−1^K^−1^)	T_max_ (°C)	%residue (wt%)	Latent heat (J∙g^−1^)	pHRR (kW∙m^−2^)(W∙g^−1^)*	THR (MJ∙m^−2^) (kJ∙g^−1^)**	LOI	UL‐94	Ref
Epoxy resin	Pure epoxy resin	0.26	387–418	5.6 @ 800°C, 13.3 @ 700°C	−	1068, 568*	76, 33.2**	21.5–26	N.R.	[207, 208, 209]
	4.5 % DOPO	−	−	−	−	724	73	31.5	V‐1	[207]
	10% DOPO‐ABZ	−	350	20.2 @ 800°C	−	609	67	32	V‐0	[207]
	2% CNT	−	402	19.9 @700°C	−	384*	23.0**	33.5	−	[209]
	2% N‐doped CNT	−	404	19.1 @700°C	−	439*	24.8**	31.5	−	[209]
	2% O‐doped CNT	−	401	23.4 @700°C	−	348*	22.3**	35	−	[209]
Paraffin	Pure paraffin	0.2–0.41	∼290	−	191.0– 242	−	−	−	−	[208, 210, 211]
	20 wt% graphite + 20 wt% boron nitride	1.02–1.20	−	−	95.9–138.0	−	−	−	−	[210]
	11% epoxy + 44% APP + 1% EG	0.47	−	−	−	−	155	−	NR	[208]
	11% epoxy + 44% MCH + 1% EG	0.61	−	−	∼800	−	107.5	−	V0	[208]
	5% carbon fiber	1.45	∼260	−	−	−	−	−	−	[211]
	5% graphene nanoplatelets	0.91	∼280	−	−	−	−	−	−	[211]
	20% EG	2.894	−	−	121.4	−	−	−	−	[212]
	10% EG + 10% SiC	4.086	−	−	122.2	−	−	−	−	[212]
	10% EG + 10% IFR	2.276	−	−	120.6	−	−	−	−	[212]
Polyamide 6 (Nylon 6)	Pure PA6	−	456	0.05 @ 700°C	−	959	110	−	NR	[213]
	15% aluminum diethyl phosphinate	−	434	1.76 @ 700°C	−	802	101	−	V‐0	[213]
	15% aluminum methylmethoxyphosphonate	−	387	9.01 @ 700°C	−	394	87	−	V‐1	[213]
Polydimethyl siloxane (PDMS)	Pure PDMS	0.27	−	50.30 @ 800°C	−	−	−	27.3	V0	[212]
	2% EG	0.35	−	35.61 @ 800°C	−	−	−	32	V0	[212]
	2% BN	0.38	−	53.59 @ 800°C	−	−	−	32.5	V0	[212]
	17% EG + 17% BN + 6% MP	1.16	−	42.29 @ 800°C	−	−	−	39.7	V0	[212]
Polyethylene glycol (PEG)	Pure PEG	−	−	−	183.76		−	−	−	[214]
	MDI + HA + 3% EG	2.14	−	−	138.75	∼480 m^2^∙s^−1^	∼ 100	−	NR	[214]
	MDI + HA + 3% EG + 20% HCCP coating	1.13	−	−	112.38	685 m^2^∙s^−1^	∼180	−	V0	[214]
Polyurethane (PU), thermoplastic	Pure thermoplastic PU	−	340–2, 411–420, 559	0.3 @ 750C, 4.96 @800C	−	964–1389	49–113.4, 20.8**	22.5 vol%	NR	[215, 216]
	0.5% MoSe2 nanosheets	−	341, 411, 600	5.26 @ 750C	−	1516	48	−	−	[215]
	2.0% Mo‐DOPO	−	343, 412, 603	7.43 @ 750C	−	865	44	−	−	[215]
	9% APP	−	303, 370	19.74 @ 800C	−	197.3	46.3	27	V‐0	[216]
	7.5% APP + 1.5% NCM	−	316, 384	16.53 @ 800C	−	218.9	56.7	24.8	V‐0	[216]
Polyurethane (PU), rigid foam	Pure rigid PU foam	0.018–0.028	327	11.8 @ 800C	−	343, 262.7*	24.9, 20.8**	19.3	HBF	[217]
	TCPP (2% P)	−	186, 331	15.5 @ 800C	−	199.4, 193.4*	21.8, 20.2**	23.2	HF‐1	[217]
	PLF140 (2% P)	−	230, 311	22.7 @ 800C	−	252.8, 228.7*	18.8, 17.5**	22.2	HF‐1	[217]
	AAM‐DOPO (2% P)	−	318, 404	29.8 @ 800C	−	214.2, 139.2*	19.3, 17.1**	22.2	HF‐1	[217]

#### 4.2.1 Identification of Key Phase Change/Decomposition Temperatures

Preliminary tests using TGA or differential scanning calorimeter (DSC) can identify key phase change or degradation temperatures. Flame‐retardant materials in a battery must be thermally stable at operational temperatures.

TGA measures the change in mass as a material is heated to high temperatures. A sudden change in mass can signal a chemical change, such as the decomposition of a material. Often measured concurrently, differential thermal analysis (DTA) compares the temperature of the sample to a standard. Even if there is no mass loss, DTA can detect transition temperatures and differentiate between endo‐ and exothermic transitions. DSC measures the amount of energy difference needed to gradually increase the temperature of a material and a control sample. It can detect both endo‐ and exothermic processes, but unlike DTA, it can quantify the energy of a certain transition.

**FIGURE 10:**
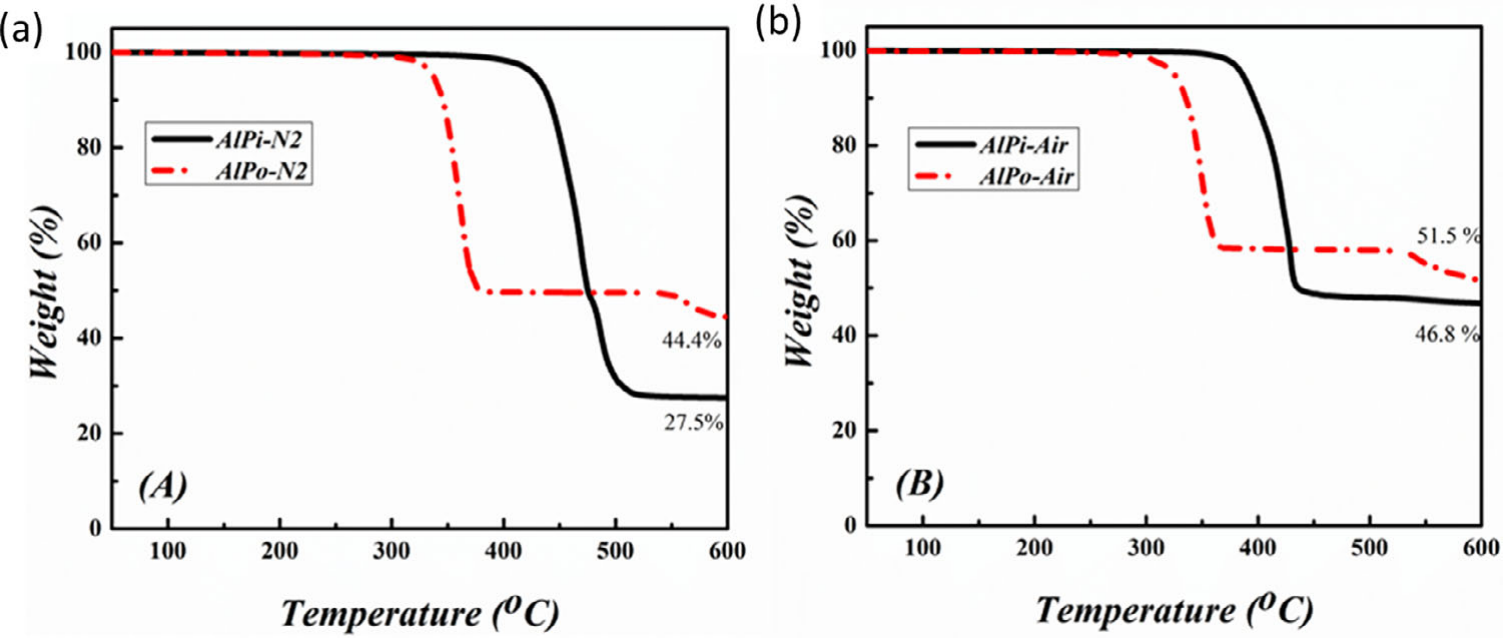
The TGA curves of two flame‐retardant fillers, AlPi and AlPo, in both (a) N2 and (b) air. In air, lower percent mass loss and lower degradation temperature were observed [213]. Copyright 2023, RSC Advances.

TGA and DTA experiments are typically performed under a stream of nitrogen gas, but performing them in air can provide additional information. Vothi et al. filled Nylon 6 with an aluminum methylmethoxyphosphonate‐based flame retardant and tested the sample in both air and nitrogen/inert atmosphere [213]. As illustrated in Figure 10, the flame‐retardant materials exhibited lower initial mass loss temperature and max mass loss rate in air than those in nitrogen. The air expedited the creation of a char layer as the material oxidized, leading to lower max percent mass loss. Conducting TGA in air made it easier to compare TGA findings to cone calorimeter data.

Besides identifying decomposition temperatures, DSC is also employed to examine the energy and temperature of transitions in PCMs (Table 4). Paraffin‐based PCMs exhibit single‐phase changes, typically between 50°C and 70°C. Alternatively, paraffin‐free PCM, such as those utilizing hydrated salts, can possess many phase transitions. Miao et al. investigated the latent heats of fusion and solidification for calcium chloride hexahydrate (CCH) based PCMs using DSC [164]. With the highest latent heat of fusion and most consistent latent heat of solidification during cycling, the PCM with 50% CCH was confirmed to have the most efficient hydrated and crystalline structure, ultimately exhibiting the best thermal stability. Using TG‐DSC (Thermogravimetry‐Differential Scanning Calorimetry) at higher temperatures, the authors identified two dehydration regimes, quantifying the reaction heats of the 50°C to 100°C and 114°C to 147°C transitions. Here, DSC was crucial for determining the thermal stability of the PCMs and quantifying the absorption of heat before testing in a TRP platform.

In summary, TGA, DTA, and DSC examine the thermal behavior of flame‐retardant materials, allowing researchers to easily compare candidates and predict flame‐retardant properties. TGA has been used in both nitrogen and air atmospheres to identify the thermal degradation of flame retardants. Meanwhile, DSC quantifies the energy a material can absorb during phase change or degradation processes, benchmarking the thermal stability of flame‐retardant materials.

#### 4.2.2 Kinetic Characterization of Heat Distribution, Absorption, and Release

Beyond identifying the transition temperature of a material, the kinetic behavior of heat is also an important distinction for TRP because flame‐retardant materials possess a certain thickness between packs, cells, or modules. The thermal conductivity, in the unit of W/m·K, is defined as the amount of heat that passes through a material with a unit thickness per unit time and area while the temperature difference across the material is one degree Kelvin. The thermal conductivity describes how well a material can transfer heat. The thermal diffusivity, in the unit of m^2^/s, describes how quickly heat travels through and affects the material, and a high diffusivity means heat travels quickly through the material. Thermal conductivity and diffusivity can also change at high temperatures. The thermal conductivity and thermal diffusivity are two ways to characterize the flux of heat throughout a material. Some common values of each are presented in Table 4.

There are many ways to measure thermal conductivity and thermal diffusivity. One example is the hot disk method, where a thin, insulated nickel probe is placed between two identical and smooth materials. The probe simultaneously heats electrically and measures the temperature change through resistance. The hot disk method is common in commercial Thermal Constant Analyzers. Another method is laser flash analysis, in which a sample with a certain thickness is heated from the bottom as a detector records the temperature change on the top. A myriad of other methods, including the hot wire, transient plane source, water flow plate method, and 3ω principle for thin films, also exist.

**FIGURE 11:**
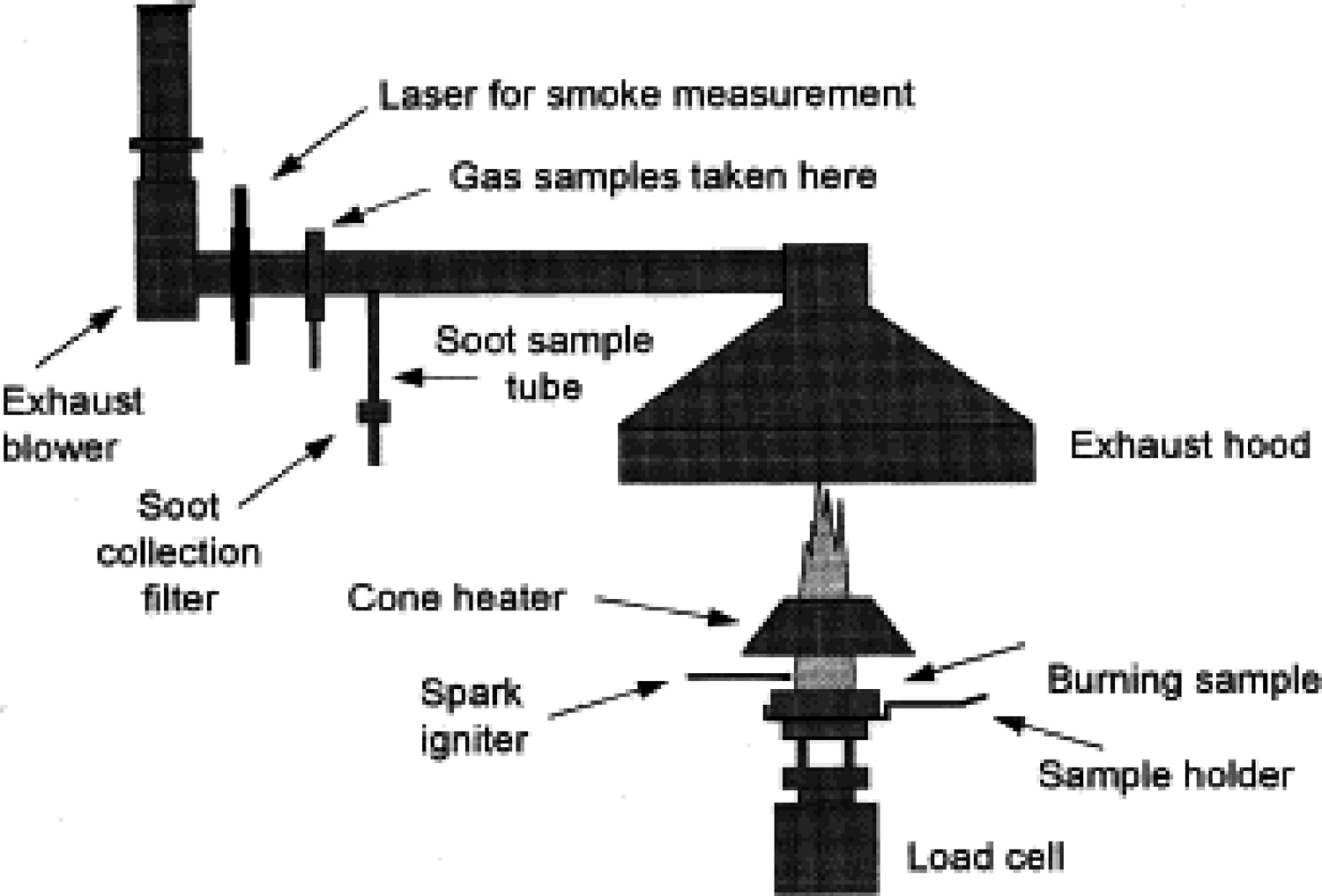
The basic form of a cone calorimeter [218]. Copyright 1999, Elsevier.

Cone calorimetry is another common method for testing the response of a material to heat [218]. As shown in Figure 11, a conical heater radiates a constant and even flux of heat above the sample. As the sample burns and emits gas, the amount of oxygen depleted is directly converted to the release of heat through oxygen consumption calorimetry, where one kilogram of oxygen consumed corresponds to 13.1 × 10^3^ kJ of heat released. The emitted gas and soot can also be collected and analyzed to find the total smoke release. In addition, many cone calorimeters feature a balance that can measure the mass loss of a sample in relation to the heat release.

In the published literature, the International Standard for Standardization ISO 5660‐1 with 35 kW/m^2^ or 50 kW/m^2^ irradiation is the most common, but the American Society for Testing and Materials ASTM E1354 is similar and sometimes used. In ISO 5660‐1, three samples with comparable mean heat release rates at 180s must be obtained to reliably report the value. In general, the time and value of the peak heat release rate (pHRR) are related to the flammability of a material. If the material emits a large amount of heat in a very short time, it can be considered flammable. In reference, polymers can reach a pHRR of over 1000 kW/m^2^ within minutes. Total heat release (THR) is how much energy is released in total. When a sample begins to sustain flames, time to ignition (TTI) is recorded, measuring the ignitability. Occasionally, the fire performance index (the TTI divided by pHRR) and fire growth index (pHRR divided by the time at pHRR) were reported. Total smoke release/production (TSR/TSP) is the total amount of smoke detected from the sample in m^2^/m^2^. More information, such as the mass loss and effective heat of combustion, can also be obtained with cone calorimetry.

For example, Wang et al. used cone calorimetry to test a thermoplastic polyurethane filled with ammonium phosphate (APP) and recycled NCM material [216]. As the ratio APP:NCM decreased in TGA samples, the temperature of the initial mass loss and max mass loss rate increased, suggesting that the NCM increased the thermal stability of the material more than APP. Despite this, the NCM made the material more flammable, as seen with cone calorimetry. At a lower APP:NCM ratio, PHRR and THR increased as the material burnt more. In addition, the TTI and pHRR are faster. With this, the authors prove that adding NCM could increase thermal stability but also increase the flammability of the material. In this case, experiments using cone calorimetry were essential to provide an understanding of how heat may impact the material.

Pyrolysis combustion flow calorimetry (PCFC), or microscale combustion calorimetry, can also be used to quantify the heat release of flame‐retardant samples. In PCFC, a few milligrams of sample are heated to a high temperature. Gases released from the degradation process are combusted separately. Like the cone calorimetry, the HRR is calculated from the amount of oxygen depleted during the combustion process. PCFC is particularly useful for elucidating the flame retardant mechanism. For example, Shabestari et al. investigated nitrogen‐ and oxygen‐doped carbon nanotubes (CNT) imbued in epoxy resin [209]. With PCFC, it was found that the pHRR and THR of the oxygen‐doped CNTs were lower than those of the nitrogen‐doped CNT or CNT alone. Lower pHRR and THR signify more flame retardancy and the suppression of reactive gas products. Thus, Shabesfari et al. determined that the oxygen‐doped CNTs increased charring and flame retardancy of the epoxy due to the increased oxygen content [209].

**FIGURE 12:**
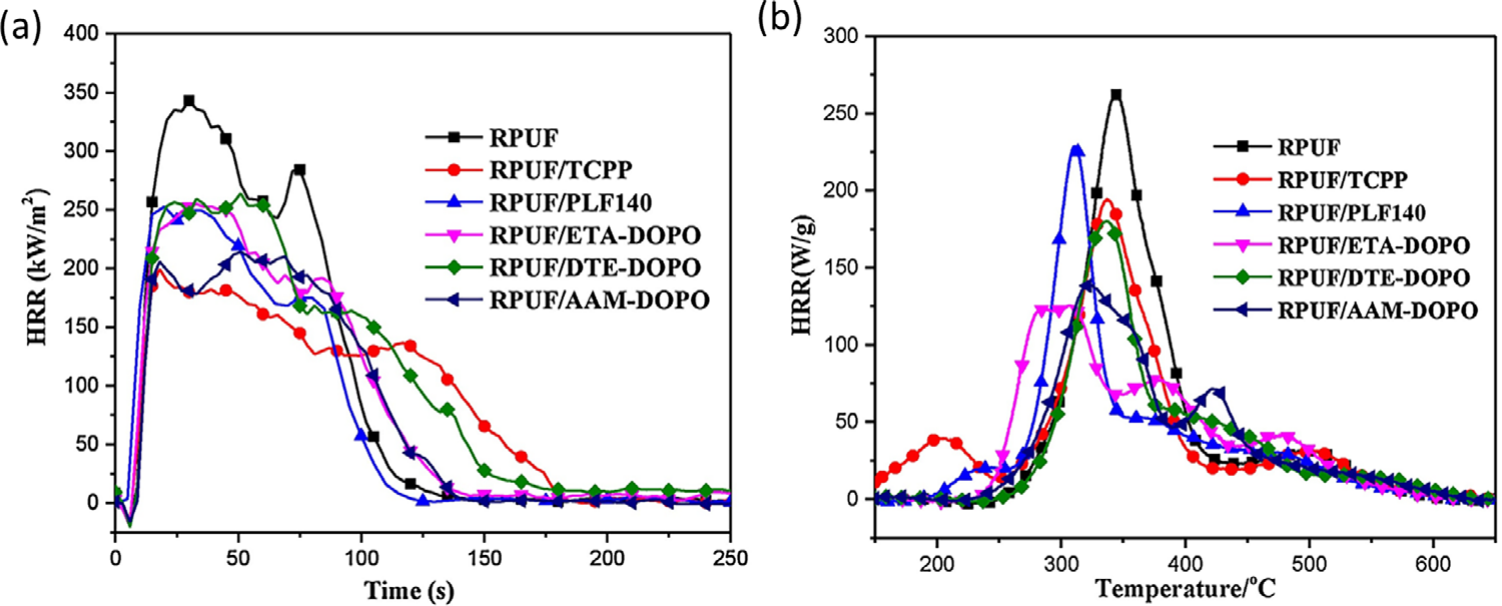
The heat release rate (HRR) uses (a) cone calorimetry and (b) PCFC of rigid polyurethane foams (RPUF) with different flame‐retardant fillers. Cone calorimetry results are plotted HRR vs. time, and PCFC results are plotted as HRR vs. temperature, reflecting the difference in information [217]. Copyright 2017, Elsevier.

While cone calorimetry accounts for both solid and gas phase reactions, PCFC combusts gases separately, yielding the heat release for only gas phase combustion reactions. In PCFC, the input gas can also be variable, which can impact the pyrolysis and provide more information about the degradation processes. The results from cone calorimetry and PCFC have been compared. As shown in Figure 12, Liu et al. used both methods to characterize DOPO‐based flame‐retardant fillers in rigid polyurethane foam [217]. In cone calorimetry, the polyurethane filled with DOPO derivatives had higher THRs than the samples with a TCPP standard, but in PCFC, the opposite was observed. TCPP relies on gas‐phase reactions to quench the flame, while the DOPO derivatives are suspected of having more condensed‐phase activity. Using PCFC, the TCPP exhibited a higher THR as its gaseous products react more than the gaseous products of DOPO derivatives.

As the combustion of materials is complex and multi‐phased, many techniques are used to deduce the mechanism of flame‐retardancy. Thermal conductivity and diffusivity are simple ways to quantify how heat moves through a material. With cone calorimetry and PCFC, the energy associated with combustion can be used to estimate how likely a material is to spread heat and fire in a battery.

#### 4.2.3 Post‐Mortem and In Situ Product Characterization for Condensed and Gas Phase Products

Accessing the identity, weight, and rate of production for both volatile and solid burning products aids the design of novel flame‐retardant materials. FTIR and Raman are two common ways to identify species in the charm. With Raman, there are also methods to determine the degree of graphitization [167]. SEM can also be used to examine how the morphology changes after burning, especially when coupled with energy dispersive X‐ray (EDS/EDX).

Degradation products in the smoke can also be monitored with online sampling using techniques such as thermogravimetric FTIR (TG‐FTIR). TG‐FTIR was used to characterize the degradation species of epoxy resin with and without a phosphorus‐doped MoS_2_ nanowire (PR‐MoS_2_) additive [24]. When the additive is present, the intensity of volatile degradation products is lower, and the peaks appear sooner. From this, the authors concluded that PR‐MoS_2_ promotes the degradation of the polymer sooner but prevents further degradation through the formation of a char layer. This is further supported by a significant reduction in signal from CO gas, ethers, and carbonyl compounds in the gas phase. Relating these results to smoke analysis in cone calorimetry, Wang et al. also found that the peak rate of CO and CO_2_ production decreased when PR‐MoS_2_ nanowires were added [24]. TG‐FTIR is a powerful tool to monitor and identify the gaseous degradation products during thermal degradation. When flame‐retardants are added, changes in volatile products can be used to evaluate the effectiveness of new gas‐phase reactions or a char layer.

Pyrolysis gas chromatography mass spectrometry (py‐GC‐MS) is also used to quantify the amount and identity of volatile products. After being quickly pyrolyzed, the gaseous products of a flame‐retardant material are separated and then ionized, yielding their identity. In Liu et al.’s work, polyurethane foams with TCPP and DOPO derivatives were tested using py‐GC‐MS [217]. Degradation of polyurethane foams alone forms various isocyanate, alcohol, and ester compounds. With the DOPO derivative, nitrile and conjugated ring products were formed, and with TCPP, phosphorus products were detected. Overall, the material with the DOPO derivative yielded fewer fragments than polyurethane alone or polyurethane with TCPP, signifying its increased activity in the condensed phase. The authors also tested the condensed phase activity with TG‐FTIR.

Both TGA and cone calorimetry measure the change in weight during the application of heat. After completing the TGA method, the %weight remaining is reported as the %char or %residue. Higher char could mean the material did not degrade fully or that more solid degradation products were formed. Information found using TGA may be supported with cone calorimetry. In Wang et al.’s work, PU with APP had a higher %char and possessed a lower total smoke release in cone calorimetry than the thermoplastic PU alone [216]. Here, the APP suppressed the creation of volatile products through the production of a char layer, lessening the smoke and increasing the amount of solid left.

#### 4.2.4 Standards for Testing Flame‐Resistance Directly

The two most common ways of proving flame‐resistance are through the UL‐94 test (ex. ASTM D3801) or by testing the limiting oxygen index (LOI, ex. ASTM D2863). Other common standards in literature are those provided by the National Standard of the People's Republic of China, namely the GB/T 2408 for flame retention and GB/T 2406 for LOI.

The UL‐94 test can be performed with vertically or horizontally held samples, named UL‐94V and UL‐94HB, respectively. Horizontal testing is used when the material is not rated in vertical testing. For vertical testing, the UL‐94 assigns a V‐0, V‐1, or V‐2 rating based on the material's ability to sustain a flame. The criterion for each rating is presented in Table 5. In brief, V‐0 materials extinguish within 10 s after flame introduction and do not produce flaming drip. V‐1 materials are the same but extinguish within 30 s. V‐2 materials extinguish within 30 s but produce flaming drips. As many materials fulfill V‐0, papers often include pictures that are used to determine the maximum time the sample carries a flame. For UL‐94HB, the rate of burning is measured, and if it burns less than 1.5 inches/min, it receives an HB rating.

**TABLE 5:** The criteria for the UL‐94 test. In addition to the conditions in the table, the sample cannot be completely burned to the clamp. After application of the flame for 10 s, t_flame_ is the amount of time a flame occurs. After the second application, t_glow_ is the amount of time the sample smolders. After 5 samples (10 total applications), the total flaming time must be below the requirement. The HB is used if sustained burning occurs.

​	1st flame, 10s​		2nd flame, 10s​			​
Rating​	t_flame,1_​	Cotton Status​	t_flame,2_​	t_glow_​	Cotton Status​	t_flame _sum across 5 samples​
V‐0​	≤ 10 s​	Not ignited​	≤ 10 s​	≤ 30 s​	Not ignited​	≤ 50 s​
V‐1​	≤ 30 s​	Not ignited​	≤ 30 s​	≤ 60 s​	Not ignited​	≤ 250 s​
V‐2​	≤ 30s​	Ignited​	≤ 30 s​	≤ 60s​	Ignited​	≤ 250 s​
HB​	After 30s of flame exposure, the burning rate is ≤ 75 mm/min for samples < 3 mm and ≤ 40 mm/min for samples with thickness from 3–13 mm.​					

Oxygen is required for combustion. The LOI is the minimum concentration of oxygen needed for a material to combust. Materials with an LOI above 27% are considered flame‐retardant, and a higher value correlates with better flame retardancy [219]. LOI testers are sold commercially, but a homebuilt system can be made using a glass column, stand, ignitor, and pressure regulators for oxygen and nitrogen gases as per the ASTM D2863 standard document. Example LOIs are reported in Table 4. In general, samples with low LOI are not likely to pass the UL‐94V test, and they must be tested using UL‐94HB.

#### 4.2.5 Mechanical Testing for Module‐Level Suitability

When designing new flame‐retardant materials for batteries, the mechanical properties are equally important as the chemical properties for their function. Battery materials come in a variety of toughness, modulus, compressibility, and flexibility to suit the needs of protective cases, cell separators, adhesives, and more. As flame‐retardant fillers are added, the mechanical properties can change dramatically, and thus, new TRP‐related materials must often balance between being thermally resistant and being mechanically stable and processable.

Tensile tests are commonly used to determine the strength, modulus, and elongation at break of a material [24, 115, 134, 148, 216, 220]. As cells breathe during charge and discharge cycles, a flexible or compressible material is needed to accommodate the volume change. Specialized testing can yield the flexural and compressive strength of the material. The flexural strength is typically found using a three‐point bend test; a sample is supported on its ends while a crosshead applies load in the middle of the sample [24, 75, 220, 221]. Standards, such as the ASTM D790, outline the calculation of flexural strength and modulus [222]. When materials exhibit superb flexibility, such as when the material does not yield or break below 5% strain, flexibility can also be described by simply bending or twisting the material 180 degrees [110, 134, 148, 168].

Compressibility is equally important for materials in cell and module separators and is commonly tested [23, 25, 28, 120, 134, 141, 143, 166]. Using a universal testing machine, the compressive strength is determined by the load force divided by the cross‐sectional area of the compressed material. The compressive modulus is obtained by multiplying the compressive strength and compressive strain, the original height or length divided by the change in height or length. A higher modulus indicates that the material is more rigid and resistant to compression. The recovery after compression can then be quantified using repetitive compressive cycles at a set strain and measuring the change in height,  max stress, and calculating the degree of plastic deformation [25]. An energy loss coefficient can also be calculated from the area under successive stress–strain cycles, and when decreasing, it suggests the material stabilizes [25, 139]. Visual inspection of the sample after compression can also reveal deficiencies, such as cracks or deformations [120, 131].

The viscoelastic properties of materials are also commonly tested. In dynamic mechanical analysis (DMA), an oscillating sinusoidal force is exerted on a sample to obtain a sample's viscosity, modulus, elasticity, and damping [25, 139, 148, 209, 221]. These properties can be extracted from each cycle, making DMA a useful tool for measuring how a material changes with time or temperature, like when quantifying fatigue resistance or a coefficient of thermal expansion [139, 146]. The results are presented as the elastic/storage modulus and loss modulus over a range of frequencies, temperatures, or cycles. When the storage modulus is higher than the loss modulus, the material is considered elastic. In Mao et al., DMA was used to investigate how the elasticity of a silica‐based aerogel changes over a broad range of frequencies, finding that the damping ratio was consistent from 0.5 to 200 Hz [25]. The expected vibrational frequency of an electric vehicle is between 1 and 50 Hz [25]. Thus, the elasticity of the aerogel will be maintained when placed in an electric vehicle. DMA for battery materials is relatively new, but it is a potentially powerful technique for seeing the viscoelastic properties of new materials.

Fire‐resistant battery materials must possess the following properties: thermal stability at typical charge and discharge temperatures, low heat release during combustion, few flammable volatile products released, and flame resistance. In addition, mechanical properties, such as strength, compressibility, and elasticity, are important to consider for realistic battery scenarios. Using a broad suite of characterization methods, these qualities can be tested prior to TRP experimentation.

Material‐level parameters should be interpreted as pathway‐specific descriptors rather than direct predictors of module‐level TRP suppression. As summarized in Table 6, thermal conductivity and thermal diffusivity primarily affect conductive heat transfer between cells, thereby influencing adjacent‐cell temperature rise and propagation delay. Heat capacity, latent heat, and endothermic decomposition determine the heat‐buffering capability of a barrier or filler material. Flame‐retardant properties, such as ignition resistance, HRR, and char formation, are more closely related to flame‐assisted propagation and secondary combustion hazards. Mechanical properties, including compressive strength, elastic recovery, and dimensional stability, determine whether a mitigation material can maintain its thickness, coverage, and interfacial contact under cell swelling, module compression, and vent‐gas impact. Therefore, the engineering relevance of material‐level properties becomes meaningful only when they are connected to module‐level indicators, such as propagation interval, peak temperature of adjacent cell, total propagation time, number of failed cells, HRR, flame duration, and pressure or gas‐release behavior. This connection also explains why material screening should be combined with representative module‐level tests and physics‐based modeling rather than relying on isolated material properties alone. For example, a material with low thermal conductivity may delay conductive heat transfer but may not effectively block flame jets or hot‐particle ejection, while a flame‐retardant material may reduce combustion hazards but still require sufficient mechanical robustness to maintain coverage under cell swelling, module compression, and vent‐gas impact.

**TABLE 6:** Linking material‐level properties to TRP pathways and module‐level metrics.

Material‐level parameter	Dominant TRP pathway	Related module‐level TRP indicator	Ref
Thermal conductivity, diffusivity	Conductive heat transfer	Peak temperature of the adjacent cell, propagation delay	[23, 131, 137, 142, 190]
Heat capacity, latent heat	Heat absorption and buffering	Time to TR onset, total propagation time	[142, 223]
Flame retardancy, char formation	Flame shielding and radiative heat blocking	HRR, flame duration, ignition risk	[131, 190, 223]
Mechanical strength, recovery	Barrier integrity under swelling/compression	Effective gap, contact resistance, barrier failure	[23, 223]
Gas permeability, sealing ability	Hot gas and particle blocking	Venting exposure, pressure evolution, flame jet impact	[223, 224]

### 4.3 Modeling Approaches

While experiments provide direct evidence of TRP behavior, modeling frameworks are indispensable for mechanism elucidation and predictive assessment of mitigation strategies at reduced risk and cost. Multiphysics approaches that couple electrochemical, thermal, and mechanical processes enable interpretation of how heat generation in the trigger cell couples into intercell transfer pathways and how material and structural parameters influence propagation sensitivity.

At the cell scale, Bernardi et al. proposed a simplified thermodynamic form of electrical heat generation that is widely used under normal charging and discharging conditions [225]. This formulation captures irreversible polarization heat and reversible entropic heat, but it does not account for the exothermic side reactions that dominate under abuse conditions. During TR, additional heat sources emerge from electrochemical side reactions such as solid‐electrolyte interphase (SEI) decomposition, electrolyte oxidation, separator shrinkage, electrolyte oxidation, and cathode oxygen release. These reactions dominate the TR onset and self‐heating rates. To describe these processes, reaction kinetic models based on the Arrhenius law have been extensively employed to characterize decomposition pathways and quantify exothermic reaction rates [226, 227, 228]. Developing an accurate single‐cell TR model thus forms the foundation for constructing conventional TRP models, as such a model provides the boundary conditions for module‐ and pack‐level simulations. Zhao et al. developed a coupled reaction–heat conduction model to describe reaction front propagation in Li‐ion battery TR within a single cell [229]. Zhang et al. introduced the concept of a thermal runaway front in long‐format cells and quantified its propagation velocity [230]. Jia et al. further described intra‐cell thermal front propagation using theoretical modeling and experimental observations, showing that the propagation velocity and front shape are strongly governed by anisotropic thermal properties, geometry, and preheating conditions [231]. These studies indicate that large‐format cells should not always be treated as spatially uniform heat sources in TRP models; instead, intra‐cell propagation can control the timing, directionality, and intensity of heat release to neighboring cells.

At the module and pack levels, modeling efforts focus on describing how the heat released by the initiating cell propagates to its neighbors. TRP models couple single‐cell TR behavior with inter‐cell heat transfer mechanisms. Depending on the required physical fidelity and computational efficiency, TRP models can be broadly categorized into three major groups: thermal resistance network models, three‐dimensional physics‐based numerical models, and data‐driven prediction models.

**FIGURE 13:**
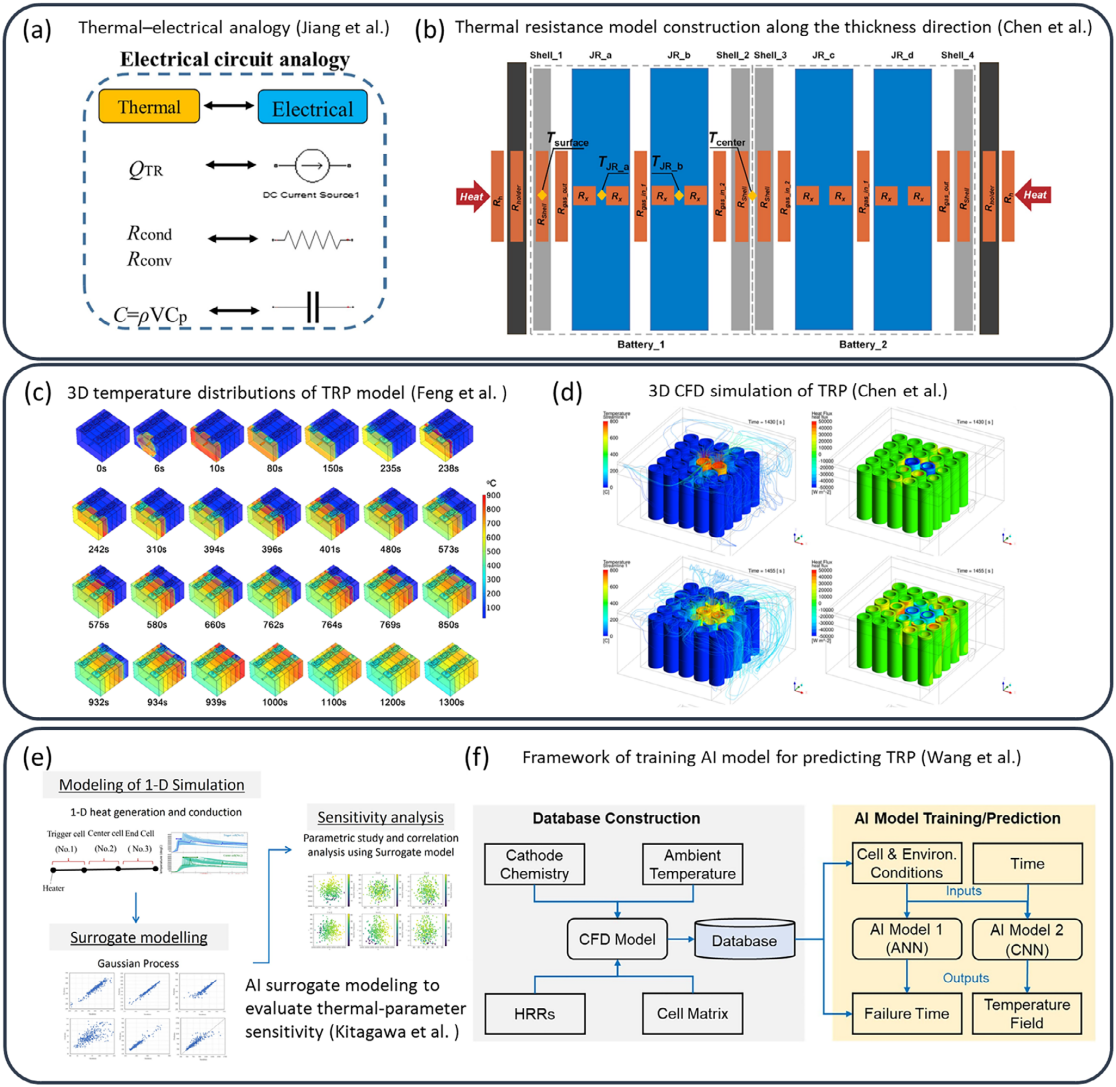
Thermal runaway propagation modeling (a) Conversion between thermal and electrical elements [232]. Copyright 2020, Elsevier. (b) Thermal resistance model construction along the thickness direction in Chen et al.’s work [233]. Copyright 2021, Elsevier. (c) A 3D thermal runaway propagation model for a large‐format lithium‐ion battery module in Feng et al.’s work [50]. Copyright 2016, Elsevier. (d) CFD visualization of temperature, gas streamlines, and heat flux in the 2 mm air‐gap TRP scenario in Chen et al.’s work [144]. Copyright 2025, Elsevier. (e) AI surrogate modeling to evaluate thermal parameter sensitivity in Kitagawa et al.’s work. Adapted from Ref. [245] under CC BY license. (f) Framework of training an AI model for predicting thermal runaway propagation in battery packs in Wang et al.’s work. Adapted from Ref. [178] under CC BY license.

As shown in Figure 13a, the thermal resistance network (TRN) model is a simplified, lumped‐parameter heat transfer framework that represents a battery module as a network of thermal nodes connected by thermal resistance and capacitances [232]. Figure 13b illustrates the one‐dimensional TRN model proposed by Chen et al., in which each battery is discretized along the thickness direction into shell and jelly‐roll nodes, and heat transfer between adjacent components is described using equivalent thermal resistances [233]. Gas thermal resistances are further introduced to account for battery swelling and rupture during TR [233]. He et al. developed a reduced‐order TRN model by redistributing heat generation among multiple characteristic nodes and introducing improved triggering criteria, enabling fast prediction of TRP in large‐scale battery systems [234]. This approach offers high computational efficiency but ignores the spatial temperature variation [234].

Three‐dimensional physics‐based numerical models provide much higher fidelity by solving the governing heat‐transfer and fluid‐dynamics equations in full spatial resolution. As illustrated in Figure 13c, Feng et al. developed a 3D TRP model based on the energy balance equation and empirical TR chemical kinetics [50]. Chen et al. further advanced a comprehensive coupled 3D computational fluid dynamics (CFD) model that incorporates TR reaction kinetics, vent‐gas flow, flaming combustion, and conjugate heat transfer to resolve spatiotemporal temperature and heat‐flux fields during module‐level propagation [144]. In this study, gas‐phase contributions have also been included, where vented gases contribute additional convective heat transfer and combustion risks. Sun et al. combined experiments and 3D CFD modeling to investigate nail‐penetration‐induced TRP in 21700 cylindrical cells and showed that appropriate interstitial thermal barriers can effectively suppress propagation by limiting heat transfer to adjacent cells [189]. These models explicitly capture the true 3D geometry, material distribution, and boundary conditions of the system. This allows accurate prediction of conduction, convection [235], radiation, and, when applicable, venting gas transport during propagation [236]. By numerically solving partial differential equations (PDE) using methods such as finite element (FE), finite volume (FV), or CFD [236, 237], they generate detailed spatiotemporal temperature fields used to evaluate the effects of cell spacing, barrier layers, vent‐gas flow [236], and structural design on TRP behavior.

To bridge the gap between computational efficiency and physical fidelity, hybrid multi‐scale modeling frameworks have also been proposed. For example, Wu et al. developed a hierarchical coupled high‐resolution CFD model with reduced‐order TRN models to capture both localized thermal phenomena and system‐level propagation dynamics [238]. The CFD model resolves localized electrochemical–thermal interactions, phase change behavior, and multi‐step TR reactions. In parallel, the TRN model represents module‐level heat transfer and intercell coupling using lumped thermal nodes and equivalent thermal resistances. Key thermo–physical parameters extracted from the CFD simulations were then mapped into the TRN framework, enabling rapid propagation prediction and multi‐parameter structural optimization. This hybrid approach enables efficient evaluation and optimization of thermal barrier and thermal management structures under TRP scenarios.

In recent years, data‐driven and AI‐based approaches have emerged as complementary strategies to overcome the limitations of purely physics‐based models [239]. Machine learning models trained on experimental and simulated datasets have been applied to predict propagation outcomes [178, 240], identifying nonlinear interactions that are difficult to capture with purely physics‐based formulations. For example, Kitagawa et al. [241] have combined one‐dimensional TRP simulations with surrogate modeling to quantify the probabilistic effects of thermal‐parameter uncertainty, as shown in Figure 13c. Wang et al. [178] developed an AI–CFD hybrid framework in which a CFD‐generated database of TRP scenarios is used to train ANN (Artificial Neural Networks) and CNN (Convolutional Neural Network) models for predicting failure time and temperature fields. Overall, these data‐driven approaches do not replace physics‐based TRP models, but instead complement them by enabling rapid prediction, uncertainty quantification, and efficient exploration of complex design spaces.

TRP modeling approaches differ substantially in their target applications, physical assumptions, computational cost, and required experimental inputs. Therefore, model selection should be guided by the specific safety question rather than by model complexity alone. As shown in Table 7, reduced‐order TRN models are useful for rapid module‐ or pack‐level screening [242], while 3D thermal models and CFD models provide higher spatial resolution for evaluating temperature gradients [243, 244], barrier placement [237], vent‐gas transport [245], and flame‐related propagation [246]. Electrochemical–thermal and mechanical–thermal models are more suitable when the propagation process is strongly affected by electrical abuse, internal short circuit, deformation, compression, or aging [31, 247, 248, 249]. Hybrid and data‐driven models can further support fast prediction and optimization, but their reliability depends on the quality, representativeness, and transparency of the training or calibration data [240, 250]. However, TR should not always be treated as a spatially uniform event. Because TR involves the abrupt release of large quantities of hot gases, liquid droplets, solid particles, and heat from localized failure regions, its development within a large cell can be described using the concept of a thermal front or thermal runaway front. Similar to a flame front, the thermal front represents the spatial progression of self‐sustaining exothermic reactions within the cell. Spatially resolved modeling is particularly important when intra‐cell thermal front propagation controls the progression of TR, especially for large‐format pouch and prismatic cells. Instead, local defects, ISCs, nonuniform heating, or separator failure may initiate localized heat generation and generate an intra‐cell thermal front before the whole cell enters runaway [102, 246, 251]. If a large‐format cell is simplified as a lumped heat source, the model may misrepresent the timing, location, and intensity of heat release, as well as the subsequent exposure of neighboring cells to hot surfaces, vent gases, flames, and particles. Therefore, TRP models for large‐format cells should consider intra‐cell temperature gradients, local reaction zones, tab location, venting direction, and spatially non‐uniform boundary conditions.

**TABLE 7:** Comparison of representative modeling approaches for TRP prediction.

‐Model	Advantages	Limitations	Application	Ref
TRN model	Low computational cost; easy parameterization; suitable for parametric studies	Limited spatial resolution; difficult to capture local hot spots, complex geometry, vent gas, and flame effects	Fast screening of module/pack TRP; cell spacing, barrier thickness, and cooling design	[234]
3D thermal model	Resolves geometry, temperature gradients, barrier placement, and cooling structures	Requires calibrated heat sources and temperature‐dependent material properties	Temperature‐field prediction; evaluation of thermal barriers and cooling structures	[72, 237, 243, 244, 252]
CFD	Captures vent‐gas transport, pressure accumulation, convective heating, jet fire, and enclosure effects	High computational cost; difficult gas‐generation, combustion, and turbulence parameterization	Venting‐path design; gas/flame propagation; confined‐space safety analysis	[245, 253, 254, 255]
Electrochemical–thermal model	Links SOC, voltage, current, reaction kinetics, and heat generation	Many uncertain parameters; difficult to generalize across chemistries and formats	TR initiation, overcharge, ISC, and electrical abuse analysis	[247, 249]
Mechanical–thermal model	Captures deformation, swelling, compression, penetration, and contact changes	Strong coupling and parameter identification are challenging	Crush/penetration abuse; module compression; barrier deformation; contact resistance	[31, 248]
Hybrid model	Balances physical fidelity and computational efficiency; supports multiscale coupling	Accuracy depends on the coupling strategy and calibration data	Multiscale TRP prediction; design optimization; physics‐informed simulation	[256, 257, 258, 259]
Data‐driven model	Fast prediction; captures nonlinear factor interactions; useful for sensitivity analysis	Requires representative datasets, transparent features, uncertainty analysis, and validation	Rapid TRP prediction; feature ranking; safety classification; digital‐twin applications	[178, 240, 250, 260]

Overall, no single modeling approach is universally optimal for TRP prediction. Reduced‐order models provide computational efficiency but may miss local heat generation and gas/flame effects, whereas high‐fidelity 3D and CFD models provide detailed spatial information but require more parameters and computational resources. Hybrid modeling and data‐driven methods offer promising routes to balance efficiency and fidelity, especially for design optimization and rapid safety screening. However, these models should be validated using independent experimental data, including ARC/DSC heat‐generation measurements, thermal‐property characterization, vent‐gas analysis, and module‐level TRP tests. Such validation is essential for translating modeling results into reliable engineering guidelines for barrier design, cooling integration, venting pathways, and cell spacing.

The overarching goal of TRP modeling is not only to reproduce TRP events but also to provide a quantitative framework for understanding heat‐generation mechanisms, propagation pathways, and their sensitivity to material and structural parameters. Within this context, such models play a critical role in guiding the development and assessment of mitigation strategies. In particular, module‐scale simulations parameterized with experimentally measured thermal conductivity, heat capacity, and flame‐retardant properties offer a cost‐effective approach to screening candidate materials and design concepts prior to large‐scale and high‐risk experiments. The insights obtained from these simulations can be further translated into rational design guidelines for cell spacing, thermal barrier placement, and material selection, thereby enabling a more systematic and model‐informed development of TRP mitigation strategies at the module and pack levels.

## 5 Future Perspectives

Although substantial progress has been made in understanding TRP and developing mitigation strategies, several critical challenges remain before TRP suppression concepts can be reliably translated into large‐scale battery systems. Future research should focus on bridging gaps across scales, standardizing evaluation methodologies, and enabling predictive, design‐oriented mitigation frameworks.

### 5.1 Toward Standardized TRP Testing and Evaluation Protocols

One major limitation in current TRP studies is the lack of standardized experimental protocols. Propagation behavior is highly sensitive to triggering methods, heating power, cell arrangement, and environmental boundary conditions, making direct comparison across studies difficult. Future efforts should aim to establish standardized TRP test configurations and reporting metrics, including trigger conditions, propagation delay definitions, failure criteria, and gas/pressure measurement standards. Such standardization would enable meaningful benchmarking of mitigation strategies and accelerate technology transfer from laboratory studies to industrial qualification.

### 5.2 Novel Sensing Technologies for TRP

TR is characterized by a cascade of exothermic reactions within a battery that drives a rapid surge in internal temperature, ultimately leading to structural degradation and catastrophic battery failure [261]. Quantitative EV safety regulations set the threshold for TR using a combination of criteria, including rapid temperature rise (*dT*/*dt* ≥ 1 K/s) and noticeable voltage drop. To ensure passenger safety, these regulations mandate a minimum 5‐min warning period before hazardous and life‐threatening conditions manifest. InLIBs, the onset of this phenomenon is typically precipitated by specific abuse conditions, predominantly classified as thermal, mechanical, and electrical abuse, alongside ISCs.

TR initiates near 80°C with SEI decomposition, exposing the graphite anode. By 100°C, subsequent electrolyte breakdown and accelerated anode–electrolyte reactions provide the primary early heat source. Between 130°C and 160°C, the polyolefin separator melts, introducing a severe risk of internal short‐circuiting that rapidly exacerbates heat generation. Above 200°C, the cathode undergoes structural phase transformations, releasing oxygen that violently reacts with organic species during the most energy‐intensive stage. Once these cascading reactions become self‐sustaining (typically >300°C), the cell is declared to enter full thermal runaway mode. This terminal phase features extreme self‐heating rates exceeding hundreds of degrees per minute, rapid gas venting, and peak temperatures above 600°C, often culminating in catastrophic fire or explosion [7, 262, 263].

The effective mitigation of TR mainly relies on the timely capture of the early‐stage signals. In a typical vehicle environment, these early‐stage signals involve anomalies in thermal, electrical, gaseous, or acoustic domains. While external temperature monitoring is intuitive, measuring the surface temperature is not efficient for detecting and remedying TR because of the low thermal diffusivity in the battery material and internal components. Therefore, internal temperature measurement has emerged as a potential strategy for detecting TR [264, 265, 266]. Embedded optical fiber sensors represent a promising detection technique; however, practical implementation at the individual cell level remains challenging, impractical, and cost‐prohibitive.

Voltage and current anomalies are readily accessible through existing battery management system (BMS) infrastructure without additional hardware, typically offering high sampling rates exceeding 100 Hz. However, electrical fault detection is inherently hindered by pack‐level signal attenuation, as parallel‐connected cells suppress weak, cell‐level perturbations and drastically reduce observability [267]. Furthermore, because battery aging alters baseline electrical characteristics, isolating fault signals from normal degradation necessitates highly complex diagnostic algorithms. To overcome these limitations, impedance‐based indicators theoretically provide superior sensitivity to internal degradation processes, driving growing research interest in simplified dynamic resistance estimation techniques.

In addition to the temperature measurement, the optical sensor can be designed to measure the internal pressure of the battery cell. A hybrid sensing technique was developed to detect early‐stage abnormal temperature and pressure signals in the battery cell [268]. Unlike external thermocouples, internal temperature measurements capture strong thermal gradients between the core and surface, which can exceed 180°C during TR. Internal pressure begins to increase due to electrolyte evaporation and gas generation triggered by SEI decomposition and early exothermic reactions. This pressure rise occurs before significant external thermal signatures are observed because surface temperature measurements rely on heat conduction from the cell interior to the exterior, which is known to be slow. As a result, monitoring internal pressure enables earlier detection of instability compared to external temperature sensing techniques. This combined analysis of internal temperature and pressure signals enables detection of the transition from stable and normal electrochemical reactions to accelerated chemical decomposition reactions.

Another TRP detection technique is acoustics. Acoustic signal monitoring provides direct detection of early events associated with the initiation and propagation of TR. High‐resolution audio systems capture the distinct transient pressure‐release waveforms generated during safety valve breakage (SVB). Specifically, vent rupture produces a broadband impulsive waveform with a decaying oscillatory tail, signifying rapid internal gas release and mechanical failure. Due to the rapid propagation of sound, these acoustic signatures register nearly instantaneously, enabling significantly faster fault identification than the slow thermal response. To evaluate practical performance, a recent study utilized comprehensive acoustic datasets containing both SVB events and ambient background noise [269]. Deep learning models are subsequently applied to isolate critical SVB signatures from environmental disturbances, establishing a robust, data‐driven framework for early TR detection.

### 5.3 Integration‐Driven Design of Multifunctional Mitigation Materials

Most flame‐retardant and thermal barrier materials are still developed and evaluated at the material level, while their effectiveness in real modules depends strongly on integration geometry, mechanical constraints, and coupling with venting and packaging design. Future mitigation strategies should move beyond single‐function materials toward multifunctional systems that simultaneously provide thermal insulation, heat absorption, flame shielding, gas control, and mechanical robustness. Co‐design of materials and module architecture—rather than material substitution alone—will be essential for achieving reliable TRP suppression under realistic operating conditions.

### 5.4 Linking Material Characterization to TRP‐Scale Performance

Although material‐level characterization techniques such as TGA, DSC, and cone calorimetry are widely used, a more mechanism‐oriented understanding of material behavior under TR conditions is still needed. Rather than relying solely on generic molecular‐level descriptions, future studies should emphasize temperature‐dependent physicochemical evolution, including phase transitions, thermal decomposition pathways, and structural transformations under high heat flux. Such processes critically govern heat absorption, gas release, char formation, and mechanical integrity, which collectively determine the effectiveness of mitigation materials during TRP. This is particularly important for multi‐component systems, where interfacial stability and component compatibility strongly influence macroscopic thermal protection performance.

It is worth emphasizing that the quantitative linkage between the results obtained from various characterization techniques and module‐level TRP behavior remains insufficiently established. Future work should focus systematically on correlating intrinsic material structures and properties (e.g., thermal conductivity, decomposition enthalpy, flammability indices) with propagation‐related metrics such as delay time, peak temperature, and number of failed cells. Establishing such structure–property–propagation relationships would provide design guidelines for materials, enabling systematic strategies to rationally screen mitigation materials and reduce reliance on costly module‐scale testing.

### 5.5 Advancing Multiscale and Hybrid Modeling Frameworks

Modeling approaches for TRP are evolving from simplified thermal networks toward high‐fidelity multiphysics simulations and data‐driven methods. However, challenges remain in balancing physical accuracy with computational efficiency, especially for large battery packs. Future modeling efforts should emphasize multiscale and hybrid frameworks that couple reduced‐order thermal network models with localized CFD or reaction‐kinetics models, supported by experimental validation. In parallel, AI‐assisted surrogate models trained on high‐quality simulation and experimental datasets offer promising pathways for rapid design optimization and uncertainty quantification toward system‐level safety design for next‐generation batteries.

### 5.6 Design Integration Under Evolving Battery Architectures

As battery technologies continue to evolve toward higher energy density, larger formats, and new chemistries, TRP mitigation must be considered as an integral component of system‐level safety design rather than an add‐on solution. Future research should extend TRP studies to emerging battery systems, including large‐format cells, solid‐state batteries, and high‐voltage chemistries, while accounting for aging, manufacturing variability, and abuse scenarios relevant to real‐world applications. Ultimately, effective TRP suppression will require coordinated advances in materials science, thermal management, mechanical design, and safety standards.

### 5.7 Economy and Cost Models for TRP Materials

The economics of a solution are equally important. A variety of models have been developed to estimate the cost of materials, plant operations, and more for battery production, but these overlook the cost of TRP‐preventing materials. This section includes a quick discussion of commonly used cost modeling strategies. The complexity of battery manufacturing is briefly mentioned.

BatPaC (Battery Performance and Cost model), a popular public resource from Argonne National Lab, converts user inputs into pack and manufacturing costs presented in dollars per kWh [270]. As of publishing, BatPaC version 6.0 is the most recent update. Users can change variables relating to the cell chemistry (i.e., electrode coupling parameters, electrode compositions, separator thickness), battery design (charging, power, storage, and configuration requirements), mechanical design (module‐level expansion, compression pad qualities), and battery management system (mass, volume, and cost of BMS used). Restrictions in the current version—such as the use of aluminum‐wrapped prismatic cells, steel‐enclosed modules, and liquid cooling—may limit its use. In the liquid‐cooling system, a 50:50 ethylene glycol:water solution flows within a steel cooling panel around the modules in parallel. Based on BatPaC 5.0, the BatPaC program does not have options for TRP‐related materials between cells or modules, only considering insulation for the pack jacket. These materials, typically made of polymers, are considered low‐cost, especially when compared to the metals within the cell. Few to no studies calculate the expected costs of the TRP‐preventing materials discussed. Additional cooling costs could also be required to offset the material's insulating behavior.

Other models, such as EverBatt by Argonne National Lab [271] and CellEST [272, 273], highlight the use of recycled materials and the impact of manufacturing processes, respectively. Bottom–up and process‐based models are most common, but there is no agreed‐upon universal cost model. Many models also do not consider real‐world volatility in material cost [274]. Although widespread, more development is needed in the current cost models. Given the commonality of TRP‐preventing materials in modern battery packs, future models should consider their use.

Beyond cost analysis, a complete tear‐down can be used to examine the complexity of battery pack and module designs. For EVs, Iceberg by Caresoft offers packages that create 3D models of battery pack and module designs. Through this, customers can identify deficiencies or unintended redundancies in the design. The process of battery manufacturing can be considered and optimized.

## 6 Conclusion

TRP represents one of the most critical safety challenges for lithium‐ion battery modules and packs, as the failure of a single cell can rapidly escalate into system‐level hazards. Rather than focusing solely on preventing the initiation of TR at the cell level, this review emphasizes that suppressing or delaying failure propagation is a more realistic and impactful strategy for improving the intrinsic safety of practical battery systems.

This review systematically distinguishes TR initiation from propagation and summarizes the dominant physical mechanisms governing TRP, including conductive, convective, and radiative heat transfer, vent‐gas release, flame jet impingement, and pressure‐driven effects. The strong dependence of propagation behavior on system‐level factors—such as cell spacing, module geometry, packaging and venting design, SOC, and cell chemistry—highlights that TRP is fundamentally a multi‐scale and multi‐physics problem that cannot be addressed through cell design alone.

To enable quantitative assessment and comparison of propagation behavior, commonly used engineering measurable metrics for TRP risk evaluation are reviewed, spanning from critical temperatures, HRR, pressure evolution, to propagation timing parameters. While ARC‐derived parameters define the thermal and energetic boundary conditions of the triggering cell, module‐level metrics such as propagation interval, propagation speed, and the number of failed cells provide direct indicators of escalation severity and mitigation effectiveness. Together, these metrics form a practical framework for evaluating TRP suppression strategies across different experimental platforms and system configurations.

A major focus of this review is the role of passive flame‐retardant and thermal barrier materials in suppressing TRP. From a functional perspective, these materials mitigate propagation through heat absorption and thermal buffering, low‐conductivity insulation, flame and gas suppression, and, increasingly, through multifunctional composite designs that integrate several mechanisms within a single barrier. Experimental evidence consistently demonstrates that appropriately designed passive barriers can significantly delay propagation, reduce peak temperatures, and limit the spatial extent of failure, even under severe abuse conditions. Importantly, the effectiveness of these materials depends not only on intrinsic properties but also on their engineering integration within battery modules, including thickness, placement, mechanical compatibility, and interaction with venting pathways.

Experimental TRP platforms and modeling approaches are reviewed as complementary tools for mechanism elucidation and mitigation evaluation. Comparative propagation experiments provide direct, application‐relevant validation of suppression strategies, while physics‐based and data‐driven models enable efficient exploration of design parameters and system‐level optimization. Material‐level characterization provides preliminary but essential information on the thermal, flammability, and mechanical behaviors of flame‐retardant materials, enabling quantitative comparison among candidate mitigation solutions. Common characterization techniques include TGA, DSC, thermal conductivity measurements, cone calorimetry, limiting oxygen index (LOI), UL‐94 vertical burning tests, and compressibility or mechanical integrity tests. Together, these approaches bridge the gap between material‐level characterization and module‐ or pack‐level safety performance.

Overall, this review highlights that effective mitigation of TRP requires an integrated, engineering‐oriented approach that combines mechanistic understanding, quantitative metrics, passive material design, and system‐level integration. By consolidating current knowledge across experiments, materials, and modeling, this work aims to provide a coherent framework to guide the development of safer lithium‐ion battery systems with enhanced resistance to TRP.

## Author Contributions

**Jinrong Su**: conceptualization, data curation, formal analysis, methodology, investigation, supervision, writing – original draft, visualization. **Wonhee Cho**: writing – original draft, writing – review and editing. **Jianing Gan**: writing – original draft, methodology. **Hanghang Yan**: writing – review and editing. **Hanlong Liu**: writing – review and editing. **Lei Chen**: funding acquisition, supervision, project administration, writing – review and editing, conceptualization, resources. **Xiangwei Guo**: writing – review and editing. **Rui Wang**: writing – review and editing. **Shuai Che**: writing – original draft. **Xinxin Yao**: writing – review and editing. **Anna Difelice**: writing – original draft, methodology. **Solomon Adera**: writing – review and editing, supervision. **Yaohong Xiao**: writing – review and editing. **Zhan Chen**: supervision, writing – review and editing, funding acquisition, resources. **Jayani Mawela**: writing – original draft, methodology. **Dingchuan Xue**: writing – review and editing.

## Conflicts of Interest

The authors declare no conflicts of interest.

## Data Availability

The data that support the findings of this study are available from the corresponding author upon reasonable request.

## References

[1] X. Feng , D. Ren , X. He , and M. Ouyang , “Mitigating Thermal Runaway of Lithium‐Ion Batteries,” Joule 4 (2020): 743–770.

[2] Y. Wang , X. Feng , W. Huang , X. He , L. Wang , and M. Ouyang , “Challenges and Opportunities to Mitigate the Catastrophic Thermal Runaway of High‐Energy Batteries,” Advanced Energy Materials 13 (2023): 2203841.

[3] H. Wang , Q. Wang , Z. Zhao , et al., “Thermal Runaway Propagation Behavior of the Cell‐to‐Pack Battery System,” Journal of Energy Chemistry 84 (2023): 162–172.

[4] Y. Jia , M. Uddin , Y. Li , and J. Xu , “Thermal Runaway Propagation Behavior Within 18,650 Lithium‐Ion Battery Packs: A Modeling Study,” Journal of Energy Storage 31 (2020): 101668.

[5] Z. Jia , X. Chen , Z. Zhao , et al., “Thermal Runaway Propagation Path and Fire Risk Assessment in Electric Vehicles Based on Full‐Vehicle Experiments,” Energy 336 (2025): 138484.

[6] S. Gao , L. Lu , M. Ouyang , et al., “Experimental Study on Module‐to‐Module Thermal Runaway‐Propagation in a Battery Pack,” Journal of The Electrochemical Society 166 (2019): A2065–A2073.

[7] D. He , J. Wang , Y. Peng , et al., “Research Advances on Thermal Runaway Mechanism of Lithium‐Ion Batteries and Safety Improvement,” Sustainable Materials and Technologies 41 (2024): 01017.

[8] Z. Zhou , M. Li , L. Li , P. Zhang , and L. Yang , “Thermal Runaway in Lithium‐Ion Battery Systems With Hybrid Electrical Connections: Propagation Induced by Spontaneous Overcharge Without Heat Transfer,” Journal of Energy Chemistry 115 (2026): 832–846.

[9] G. Wang , P. Ping , D. Kong , et al., “Advances and Challenges in Thermal Runaway Modeling of Lithium‐Ion Batteries,” The Innovation 5 (2024): 100624. 38746910 10.1016/j.xinn.2024.100624 PMC11089405

[10] X. Hu , F. Gao , Y. Xiao , et al., “Advancements in the Safety of Lithium‐Ion Battery: The Trigger, Consequence and Mitigation Method of Thermal Runaway,” Chemical Engineering Journal 481 (2024): 148450.

[11] D. Kong , G. Wang , P. Ping , and J. Wen , “A Coupled Conjugate Heat Transfer and CFD Model for the Thermal Runaway Evolution and Jet Fire of 18650 Lithium‐Ion Battery Under Thermal Abuse,” Transportation 12 (2022): 100157.

[12] X. Lai , S. Wang , H. Wang , Y. Zheng , and X. Feng , “Investigation of Thermal Runaway Propagation Characteristics of Lithium‐Ion Battery Modules Under Different Trigger Modes,” International Journal of Heat and Mass Transfer 171 (2021): 121080.

[13] S. Gao , X. Feng , L. Lu , et al., “An Experimental and Analytical Study of Thermal Runaway Propagation in a Large Format Lithium Ion Battery Module With NCM Pouch‐Cells in Parallel,” International Journal of Heat and Mass Transfer 135 (2019): 93–103.

[14] Z. Wang , N. Mao , and F. Jiang , “Study on the Effect of Spacing on Thermal Runaway Propagation for Lithium‐Ion Batteries,” Journal of Thermal Analysis and Calorimetry 140 (2020): 2849–2863.

[15] J. Fang , J. Cai , and X. He , “Experimental Study on the Vertical Thermal Runaway Propagation in Cylindrical Lithium‐Ion Batteries: Effects of Spacing and State of Charge,” Applied Thermal Engineering 197 (2021): 117399.

[16] R. Peng , D. Kong , P. Ping , et al., “Experimental Investigation of the Influence of Venting Gases on Thermal Runaway Propagation in Lithium‐Ion Batteries With Enclosed Packaging,” eTransportation 23 (2025): 100388.

[17] Z. Jia , S. Wang , P. Qin , et al., “Comparative Investigation of the Thermal Runaway and Gas Venting Behaviors of Large‐Format LiFePO_4_ Batteries Caused by Overcharging and Overheating,” Journal of Energy Storage 61 (2023): 106791.

[18] M. Theiler , A. Baumann , and C. Endisch , “Influence of Inhomogeneous State of Charge Distributions on Thermal Runaway Propagation in Lithium‐Ion Batteries,” Journal of Energy Storage 95 (2024): 112483.

[19] M. Chen , D. Ouyang , J. Weng , J. Liu , and J. Wang , “Environmental Pressure Effects on Thermal Runaway and Fire Behaviors of Lithium‐Ion Battery With Different Cathodes and State of Charge,” Process Safety and Environmental Protection 130 (2019): 250–256.

[20] C. Lee , A. O. Said , and S. I. Stoliarov , “Impact of State of Charge and Cell Arrangement on Thermal Runaway Propagation in Lithium Ion Battery Cell Arrays,” Transportation Research Record: Journal of the Transportation Research Board 2673 (2019): 408–417.

[21] A. O. Said , C. Lee , and S. I. Stoliarov , “Experimental Investigation of Cascading Failure in 18650 Lithium Ion Cell Arrays: Impact of Cathode Chemistry,” Journal of Power Sources 446 (2020): 227347.

[22] Z. Jia , Z. Huang , H. Zhai , et al., “Experimental Investigation on Thermal Runaway Propagation of 18,650 Lithium‐Ion Battery Modules With Two Cathode Materials at Low Pressure,” Energy 251 (2022): 123925.

[23] S. Chen , X. Wei , H. Wu , et al., “Multi‐Functional Thermal Barrier Suppresses Battery Thermal Runaway Propagation and Degradation,” Renewable and Sustainable Energy Reviews 223 (2025): 116056.

[24] J. Wang , Y. Zhou , Z. Wang , et al., “Fire‐Resistant and Mechanically‐Robust Phosphorus‐Doped MoS_2_/Epoxy Composite as Barrier of the Thermal Runaway Propagation of Lithium‐Ion Batteries,” Chemical Engineering Journal 497 (2024): 154866.

[25] T. Mao , Y. Xiao , X. Cheng , et al., “Effects and Mechanisms of Thickness‐Tunable Aerogel Thermal Barriers on Suppression of Thermal Runaway Propagation in Large‐Format Prismatic Lithium‐Ion Batteries,” Materials Today Energy 53 (2025): 101996.

[26] F. Menz , B. Bausch , J. K. Barillas , O. Böse , M. A. Danzer , and M. Hölzle , “Preventing Thermal Runaway Propagation in Lithium‐Ion Batteries: Model‐Based Optimization of Interstitial Heat‐Absorbing Thermal Barriers,” Journal of Power Sources 584 (2023): 233578.

[27] Y. Mao , Y. Chen , Y. Ye , Y. Chen , and M. Chen , “Comparative Study of Thermal Runaway Propagation and Material Barrier Effect of Lithium‐Ion Batteries,” Batteries 11 (2025): 214.

[28] Y. Yu , Z. Li , J. Wang , W. Mei , P. Duan , and Q. Wang , “Advanced Ultra‐Pressure‐Resistant Three‐Phase Composite Insulation: Halting Thermal Runaway in Lithium‐Ion Batteries,” Energy Storage Materials 76 (2025): 104148.

[29] S. K. WONG , Y. Hong , C. Xu , Y. Peng , S. Zheng , and X. Feng , “A Thermo‐Mechanical‐Chemical Composite Barrier for Suppressing Thermal Runaway Propagation in NCM811 Battery Module,” eTransportation 25 (2025): 100451.

[30] Q. Huang , J. Deng , X. Li , et al., “Advanced Functional Epoxy Reinforcement Framework for Flame‐Retardant Phase Change Materials Toward Battery Thermal Runaway Mitigation,” Chemical Engineering Journal 528 (2026): 172532.

[31] H. Li , D. Zhou , M. Zhang , B. Liu , and C. Zhang , “Multi‐Field Interpretation of Internal Short Circuit and Thermal Runaway Behavior for Lithium‐Ion Batteries Under Mechanical Abuse,” Energy 263 (2023): 126027.

[32] R. Xiong , R. Yang , Z. Chen , W. Shen , and F. Sun , “Online Fault Diagnosis of External Short Circuit for Lithium‐Ion Battery Pack,” IEEE Transactions on Industrial Electronics 67 (2020): 1081–1091.

[33] Z. An , Y. Zhao , X. Du , T. Shi , and D. Zhang , “Experimental Research on Thermal‐Electrical Behavior and Mechanism During External Short Circuit for LiFePO_4_ Li‐Ion Battery,” Applied Energy 332 (2023): 120519.

[34] B. Zhang , Z. Chen , Q. Tao , M. Jiao , P. Li , and N. Zhou , “Characterization Study on External Short Circuit for Lithium‐Ion Battery Safety Management: From Single Cell to Module,” Journal of Energy Storage 99 (2024): 113239.

[35] P. Liu , S. Li , K. Jin , et al., “Thermal Runaway and Fire Behaviors of Lithium Iron Phosphate Battery Induced by Overheating and Overcharging,” Fire Technology 59 (2023): 1051–1072.

[36] W. H. Yan , W. X. Huang , Y. Yang , Z. W. Wei , H. S. Zhen , and Y. Lin , “Research on Overcharge Mitigations and Thermal Runaway Risk of 18650 Lithium‐Ion Batteries,” Journal of Energy Storage 120 (2025): 116372.

[37] J. Sun , Z. Liu , Z. Gong , et al., “Thermal Runaway Behavior and Debris Analysis of Lithium‐Ion Batteries Under Different Overcharge Rates,” Applied Thermal Engineering 286 (2026): 129349.

[38] L. Qian , Y. Yi , W. Zhang , C. Fu , C. Xia , and T. Ma , “Revealing the Impact of High Current Overcharge/Overdischarge on the Thermal Safety of Degraded Li‐Ion Batteries,” International Journal of Energy Research 2023 (2023): 1–14.

[39] J. Su , H. Yan , Y. Xiao , et al., “Dual‐Scale Model Enabled Explainable‐AI Toward Decoding Internal Short Circuit Risk of Lithium Metal Batteries,” Energy Storage Materials 78 (2025): 104286.

[40] H. Yan , J. Su , Z. Zhao , et al., “Toward Deciphering the Internal Menace of Battery Safety: Soft Short Circuit versus Hard Short Circuit?,” Advanced Energy Materials 15 (2025): 2500275.

[41] X. Han , S. Mao , Y. Wang , et al., “Manipulation of Lithium Dendrites Based on Electric Field Relaxation Enabling Safe and Long‐Life Lithium‐Ion Batteries,” Nature Communications 16 (2025): 3699. 10.1038/s41467-025-58818-y PMC12008231 40251155

[42] Z. Huang , C. Zhao , H. Li , W. Peng , Z. Zhang , and Q. Wang , “Experimental Study on Thermal Runaway and Its Propagation in the Large Format Lithium Ion Battery Module With Two Electrical Connection Modes,” Energy 205 (2020): 117906.

[43] M. Zhou and J. Xu , “Coupling of Stack Pressure and Interface Contact Dictates SEI Stability in Li‐Metal Solid‐State Batteries,” Acta Materialia 314 (2026): 122327.

[44] L. Zhang , L. Liu , A. Terekhov , D. Warnberg , and P. Zhao , “Thermal Runaway of Li‐Ion Battery With Different Aging Histories,” Process Safety and Environmental Protection 185 (2024): 910–917.

[45] J. Zhang , T. Long , X. Sun , et al., “Mechanism Investigation on Microstructure Degradation and Thermal Runaway Propagation of Batteries Undergoing High‐Rate Cycling Process,” Journal of Energy Chemistry 113 (2026): 1013–1029.

[46] Z. Cheng , Y. Min , P. Qin , et al., “A Distributed Thermal‐Pressure Coupling Model of Large‐Format Lithium Iron Phosphate Battery Thermal Runaway,” Applied Energy 378 (2025): 124875.

[47] Y. Wu , W. Zhang , X. Rui , et al., “Thermal Runaway Mechanism of Composite Cathodes for All‐Solid‐State Batteries,” Advanced Energy Materials 15 (2025): 2405183.

[48] X. Tan , S. Sun , and J. Xu , “Decoding the Liquid‐Solid Interplay for the Mechanical Behaviors of the Battery Materials,” Journal of Power Sources 653 (2025): 237566.

[49] W. Yan , Z. Wang , and S. Chen , “Quantitative Analysis on the Heat Transfer Modes in the Process of Thermal Runaway Propagation in Lithium‐Ion Battery Pack Under Confined and Semi‐Confined Space,” International Journal of Heat and Mass Transfer 176 (2021): 121483.

[50] X. Feng , L. Lu , M. Ouyang , J. Li , and X. He , “A 3D Thermal Runaway Propagation Model for a Large Format Lithium Ion Battery Module,” Energy 115 (2016): 194–208.

[51] G. Wang , P. Ping , Y. Zhang , et al., “Modeling Thermal Runaway Propagation of Lithium‐Ion Batteries Under Impacts of Ceiling Jet Fire,” Process Safety and Environmental Protection 175 (2023): 524–540.

[52] Y. Zhang , H. Zhao , G. Wang , et al., “Effect of Flame Heating on Thermal Runaway Propagation of Lithium‐Ion Batteries in Confined Space,” Journal of Energy Storage 78 (2024): 110052.

[53] M. Zhang , W. Huang , P. Wang , et al., “Investigating Heat Transfer Mechanisms in Thermal Runaway Propagation: A Battery‐Smoke Decoupling Approach,” Process Safety and Environmental Protection 201 (2025): 107623.

[54] Q. Wang , H. Wang , C. Xu , et al., “Multidimensional Fire Propagation of Lithium‐Ion Phosphate Batteries for Energy Storage,” eTransportation 20 (2024): 100328.

[55] F. He , J. Deng , H. Wang , et al., “Fire Propagation and Suppression in Multi‐Layer Battery Systems,” Applied Thermal Engineering 276 (2025): 126945.

[56] J. Zhao , S. Lu , Y. Fu , W. Ma , Y. Cheng , and H. Zhang , “Experimental Study on Thermal Runaway Behaviors of 18650 Li‐Ion Battery Under Enclosed and Ventilated Conditions,” Fire Safety Journal 125 (2021): 103417.

[57] Z. Zhou , X. Zhou , B. Wang , K. M. Liew , and L. Yang , “Experimentally Exploring Thermal Runaway Propagation and Prevention in the Prismatic Lithium‐Ion Battery With Different Connections,” Process Safety and Environmental Protection 164 (2022): 517–527.

[58] L. Zhang , L. Liu , and P. Zhao , “Review—Understanding the Thermal Runaway Behavior of Li‐Ion Batteries Through Experimental Techniques,” Journal of the Electrochemical Society 166 (2019): A2165.

[59] Y.‐S. Duh , Y. Sun , X. Lin , et al., “Characterization on Thermal Runaway of Commercial 18650 Lithium‐Ion Batteries Used in Electric Vehicles: A Review,” Journal of Energy Storage 41 (2021): 102888.

[60] Z. Huang , X. Li , Q. Wang , et al., “Experimental Investigation on Thermal Runaway Propagation of Large Format Lithium Ion Battery Modules With Two Cathodes,” International Journal of Heat and Mass Transfer 172 (2021): 121077.

[61] Q. Wang , B. Mao , S. I. Stoliarov , and J. Sun , “A Review of Lithium Ion Battery Failure Mechanisms and Fire Prevention Strategies,” Progress in Energy and Combustion Science 73 (2019): 95–131.

[62] G. Zhou , C. Niu , Y. Kong , et al., “Research on Stimulation Responsive Electrolytes From the Perspective of Thermal Runaway in Lithium‐Ion Batteries: A Review,” Fuel 368 (2024): 131599.

[63] R. Saha , A. S. J. Alujjage , B. S. Vishnugopi , et al., “Interrogating the Thermo‐Electrochemical Instability and Safety in Lithium Metal Electrodes With Liquid Electrolytes,” Advanced Energy Materials 15 (2025): 04145.

[64] P. Ranganathan , B. S. Vishnugopi , A. Karmakar , et al., “Role of Anode Composition and Electrolyte Interactions on the Thermo‐Electrochemical Stability of Sodium‐Ion Batteries,” ACS Applied Materials & Interfaces 18 (2026): 9670–9682. 41650392 10.1021/acsami.5c19946 PMC12926938

[65] Y. He , T. Chen , Y. Zhang , et al., “EC‐Less Electrolytes for High‐Safety and Long‐Life Nickel‐Rich Lithium‐Ion Batteries,” Advanced Functional Materials 36 (2025): 17363.

[66] R. Chen , A. M. Nolan , J. Lu , et al., “The Thermal Stability of Lithium Solid Electrolytes With Metallic Lithium,” Joule 4 (2020): 812–821.

[67] H. Zhou , A. Karmakar , A. S. J. Alujjage , et al., “Mechanistic Understanding of Silicon‐Graphite Composite Anode Thermal Stability in Lithium‐Ion Batteries,” Energy Storage Materials 79 (2025): 104334.

[68] G. Zhong , H. Li , C. Wang , K. Xu , and Q. Wang , “Experimental Analysis of Thermal Runaway Propagation Risk Within 18650 Lithium‐Ion Battery Modules,” Journal of The Electrochemical Society 165 (2018): A1925–A1934.

[69] B. Mao , J. Lu , Y. Zhang , et al., “Mitigating the Cascading Effects of Thermal Runaway and Fire Propagation in Enclosed Clusters of 18,650‐Type Lithium‐Ion Batteries,” International Journal of Heat and Mass Transfer 239 (2025): 126577.

[70] C. F. Lopez , J. A. Jeevarajan , and P. P. Mukherjee , “Experimental Analysis of Thermal Runaway and Propagation in Lithium‐Ion Battery Modules,” Journal of The Electrochemical Society 162 (2015): A1905–A1915.

[71] H. Li , H. Chen , G. Zhong , Y. Wang , and Q. Wang , “Experimental Study on Thermal Runaway Risk of 18650 Lithium Ion Battery Under Side‐Heating Condition,” Journal of Loss Prevention in the Process Industries 61 (2019): 122–129.

[72] C. Jin , Y. Sun , H. Wang , et al., “Heating Power and Heating Energy Effect on the Thermal Runaway Propagation Characteristics of Lithium‐Ion Battery Module: Experiments and Modeling,” Applied Energy 312 (2022): 118760.

[73] Z. Huang , T. Shen , K. Jin , J. Sun , and Q. Wang , “Heating Power Effect on the Thermal Runaway Characteristics of Large‐Format Lithium Ion Battery With Li(Ni_1/3_Co_1/3_Mn_1/3_)O_2_ as Cathode,” Energy 239 (2022): 121885.

[74] Y. Liu , H. Niu , C. Xu , and X. Huang , “Thermal Runaway Propagation in Linear Battery Module Under Low Atmospheric Pressure,” Applied Thermal Engineering 216 (2022): 119086.

[75] Y. Zhou , L. Xiao , J. Zhang , et al., “Fire‐Retardant, Heat‐Resistant and Mechanically‐Robust Ceramic Fiber Reinforced Aerogel Composite as Barrier towards the Thermal Runaway Propagation of Batteries,” Applied Thermal Engineering 280 (2025): 128473.

[76] Q. Wuan , Y. Wu , Y. Wang , H. Cheng , and Y. Lu , “Electrolyte‐Centric Strategies for High‐Energy‐Density Lithium Metal Batteries,” EES Batteries 2 (2026): 721–754.

[77] C. Lin , H. Yan , C. Qi , et al., “Thermal Runaway and Gas Production Characteristics of Semi‐Solid Electrolyte and Liquid Electrolyte Lithium‐Ion Batteries: A Comparative Study,” Process Safety and Environmental Protection 189 (2024): 577–586.

[78] L. Zhao , Z. Han , W. Guo , et al., “An Experimental Study on Thermal Runaway Propagation Over Cyclic Aging Lithium‐Ion Battery Modules With Different Electrical Connections,” Journal of Energy Storage 89 (2024): 111823.

[79] D. Ouyang , J. Weng , J. Hu , M. Chen , Q. Huang , and J. Wang , “Experimental Investigation of Thermal Failure Propagation in Typical Lithium‐Ion Battery Modules,” Thermochimica Acta 676 (2019): 205–213.

[80] D. Ouyang , J. Liu , M. Chen , J. Weng , and J. Wang , “An Experimental Study on the Thermal Failure Propagation in Lithium‐Ion Battery Pack,” Journal of The Electrochemical Society 165 (2018): A2184–A2193.

[81] Z. Wang , T. He , H. Bian , F. Jiang , and Y. Yang , “Characteristics of and Factors Influencing Thermal Runaway Propagation in Lithium‐Ion Battery Packs,” Journal of Energy Storage 41 (2021): 102956.

[82] C. Tao , G. Li , J. Zhao , et al., “The Investigation of Thermal Runaway Propagation of Lithium‐Ion Batteries Under Different Vertical Distances,” Journal of Thermal Analysis and Calorimetry 142 (2020): 1523–1532.

[83] H. Niu , C. Chen , D. Ji , et al., “Thermal‐Runaway Propagation Over a Linear Cylindrical Battery Module,” Fire Technology 56 (2020): 2491–2507.

[84] Z. Zhou , X. Zhou , X. Ju , M. Li , B. Cao , and L. Yang , “Experimental Study of Thermal Runaway Propagation Along Horizontal and Vertical Directions for LiFePO_4_ Electrical Energy Storage Modules,” Renewable Energy 207 (2023): 13–26.

[85] D. Ouyang , J. Liu , M. Chen , J. Weng , and J. Wang , “Thermal Failure Propagation in Lithium‐Ion Battery Modules With Various Shapes,” Applied Sciences 8 (2018): 1263.

[86] L. Quanyi , Y. Xiaoying , and H. Xu , “Effect of Different Arrangement on Thermal Runaway Characteristics of 18650 Lithium Ion Batteries under the Typical Pressure in Civil Aviation Transportation,” Fire Technology 56 (2020): 2509–2523.

[87] T. Liu , J. Hu , C. Tao , X. Zhu , and X. Wang , “Effect of Parallel Connection on 18650‐Type Lithium Ion Battery Thermal Runaway Propagation and Active Cooling Prevention With Water Mist,” Applied Thermal Engineering 184 (2021): 116291.

[88] Q. Liu , Q. Zhu , W. Zhu , and X. Yi , “Influence of Aerogel Felt With Different Thickness on Thermal Runaway Propagation of 18650 Lithium‐Ion Battery,” Electrochemistry 90 (2022): 087003–087003.

[89] C. Lee , A. O. Said , and S. I. Stoliarov , “Passive Mitigation of Thermal Runaway Propagation in Dense 18650 Lithium Ion Cell Assemblies,” Journal of The Electrochemical Society 167 (2020): 090524.

[90] C. Yuan , Q. Wang , Y. Wang , and Y. Zhao , “Inhibition Effect of Different Interstitial Materials on Thermal Runaway Propagation in the Cylindrical Lithium‐Ion Battery Module,” Applied Thermal Engineering 153 (2019): 39–50.

[91] D. Zeng , L. Gagnon , and Y. Wang , “Network Thermal‐Runaway Propagation Model—An Experimental and Modeling Study,” Fire Safety Journal 140 (2023): 103899.

[92] Z. Li , P. Zhang , and R. Shang , “Effects of Heating Position on the Thermal Runaway Propagation of a Lithium‐Ion Battery Module in a Battery Enclosure,” Applied Thermal Engineering 222 (2023): 119830.

[93] P. Gao , L. Song , Z. Jia , et al., “Revealing the Contribution of Flame Spread to Vertical Thermal Runaway Propagation for Energy Storage Systems,” Journal of Power Sources 628 (2025): 235897.

[94] H. Chen , B. Gulsoy , A. Barai , P. Nakhanivej , M. J. Loveridge , and J. Marco , “Experimental and Numerical Study of Internal Pressure of Lithium‐Ion Batteries Under Overheating,” Journal of Energy Storage 116 (2025): 116066.

[95] B. Gulsoy , H. Chen , C. Briggs , T. A. Vincent , J. E. H. Sansom , and J. Marco , “Real‐Time Simultaneous Monitoring of Internal Temperature and Gas Pressure in Cylindrical Cells During Thermal Runaway,” Journal of Power Sources 617 (2024): 235147.

[96] X. Feng , S. Zheng , D. Ren , et al., “Investigating the Thermal Runaway Mechanisms of Lithium‐Ion Batteries Based on Thermal Analysis Database,” Applied Energy 246 (2019): 53–64.

[97] N. E. Galushkin , N. N. Yazvinskaya , and D. N. Galushkin , “Mechanism of Thermal Runaway in Lithium‐Ion Cells,” Journal of The Electrochemical Society 165 (2018): A1303–A1308.

[98] H. Zhou , A. S. Alujjage , M. Terese , et al., “Effect of Fast Charging on Degradation and Safety Characteristics of Lithium‐Ion Batteries With LiFePO_4_ Cathodes,” Applied Energy 377 (2025): 124465.

[99] T. He , T. Zhang , S. Gadkari , Z. Wang , N. Mao , and Q. Cai , “An Investigation on Thermal Runaway Behaviour of a Cylindrical Lithium‐Ion Battery Under Different States of Charge Based on Thermal Tests and a Three‐Dimensional Thermal Runaway Model,” Journal of Cleaner Production 388 (2023): 135980.

[100] Y. Li , W. Mei , Y. Yu , et al., “Revealing the Self‐Ignition Mechanism of Lithium Iron Phosphate Battery Modules: The Coupling Effect of Battery Inconsistency and BMS Failure,” Transportation 26 (2025): 100484.

[101] A. Karmakar , H. Zhou , B. S. Vishnugopi , J. A. Jeevarajan , and P. P. Mukherjee , “State‐of‐Charge Implications of Thermal Runaway in Li‐Ion Cells and Modules,” Journal of The Electrochemical Society 171 (2024): 010529.

[102] H. Wang , B. Liu , C. Xu , et al., “Dynamic Thermophysical Modeling of Thermal Runaway Propagation and Parametric Sensitivity Analysis for Large Format Lithium‐Ion Battery Modules,” Journal of Power Sources 520 (2022): 230724.

[103] C. Zhao , J. Sun , and Q. Wang , “Thermal Runaway Hazards Investigation on 18650 Lithium‐Ion Battery Using Extended Volume Accelerating Rate Calorimeter,” Journal of Energy Storage 28 (2020): 101232.

[104] Y. Liu , L. Ju , Z. Z. Jia , et al., “Experimental Study on the Internal Pressure Evolution of Large‐Format LiFePO_4_ Battery During Thermal Runaway,” Journal of Energy Storage 102 (2024): 114196.

[105] A. Hofmann , N. Uhlmann , C. Ziebert , O. Wiegand , A. Schmidt , and T. Hanemann , “Preventing Li‐Ion Cell Explosion During Thermal Runaway With Reduced Pressure,” Applied Thermal Engineering 124 (2017): 539–544.

[106] Z. Wang and J. Wang , “Investigation of External Heating‐Induced Failure Propagation Behaviors in Large‐Size Cell Modules With Different Phase Change Materials,” Energy 204 (2020): 117946.

[107] S. Wilke , B. Schweitzer , S. Khateeb , and S. Al‐Hallaj , “Preventing Thermal Runaway Propagation in Lithium Ion Battery Packs Using a Phase Change Composite Material: An Experimental Study,” Journal of Power Sources 340 (2017): 51–59.

[108] W. Zhang , Z. Liang , X. Yin , and G. Ling , “Avoiding Thermal Runaway Propagation of Lithium‐Ion Battery Modules by Using Hybrid Phase Change Material and Liquid Cooling,” Applied Thermal Engineering 184 (2021): 116380.

[109] J. Weng , C. Xiao , D. Ouyang , et al., “Mitigation Effects on Thermal Runaway Propagation of Structure‐Enhanced Phase Change Material Modules With Flame Retardant Additives,” Energy 239 (2022): 122087.

[110] Y. Zhao , X. Zhang , S. Cai , et al., “Experimental Study on Flexible Flame Retardant Phase Change Materials for Reducing Thermal Runaway Propagation of Batteries,” Journal of Energy Storage 89 (2024): 111721.

[111] S. A. Hallaj and J. R. Selman , “A Novel Thermal Management System for Electric Vehicle Batteries Using Phase‐Change Material,” Journal of The Electrochemical Society 147 (2000): 3231.

[112] A. Hussain , C. Y. Tso , and C. Y. H. Chao , “Experimental Investigation of a Passive Thermal Management System for High‐Powered Lithium Ion Batteries Using Nickel Foam‐Paraffin Composite,” Energy 115 (2016): 209–218.

[113] G. Karimi , M. Azizi , and A. Babapoor , “Experimental Study of a Cylindrical Lithium Ion Battery Thermal Management Using Phase Change Material Composites,” Journal of Energy Storage 8 (2016): 168–174.

[114] A. Babapoor , M. Azizi , and G. Karimi , “Thermal Management of a Li‐Ion Battery Using Carbon Fiber‐PCM Composites,” Applied Thermal Engineering 82 (2015): 281–290.

[115] D. Hu , L. Han , W. Zhou , et al., “Flexible Phase Change Composite Based on Loading Paraffin Into Cross‐Linked CNT/SBS Network for Thermal Management and Thermal Storage,” Chemical Engineering Journal 437 (2022): 135056.

[116] B. Mortazavi , H. Yang , F. Mohebbi , G. Cuniberti , and T. Rabczuk , “Graphene or h‐BN Paraffin Composite Structures for the Thermal Management of Li‐Ion Batteries: A Multiscale Investigation,” Applied Energy 202 (2017): 323–334.

[117] A. Mills , M. Farid , J. R. Selman , and S. Al‐Hallaj , “Thermal Conductivity Enhancement of Phase Change Materials Using a Graphite Matrix,” Applied Thermal Engineering 26 (2006): 1652–1661.

[118] J. Weng , D. Ouyang , X. Yang , M. Chen , G. Zhang , and J. Wang , “Alleviation of Thermal Runaway Propagation in Thermal Management Modules Using Aerogel Felt Coupled With Flame‐Retarded Phase Change Material,” Energy Conversion and Management 200 (2019): 112071.

[119] Y. Lv , W. Situ , X. Yang , G. Zhang , and Z. Wang , “A Novel Nanosilica‐Enhanced Phase Change Material With Anti‐Leakage and Anti‐Volume‐Changes Properties for Battery Thermal Management,” Energy Conversion and Management 163 (2018): 250–259.

[120] J. Li , J. Huang , and M. Cao , “Properties Enhancement of Phase‐Change Materials via Silica and Al Honeycomb Panels for the Thermal Management of LiFeO_4_ Batteries,” Applied Thermal Engineering 131 (2018): 660–668.

[121] W. Wu , X. Yang , G. Zhang , K. Chen , and S. Wang , “Experimental Investigation on the Thermal Performance of Heat Pipe‐Assisted Phase Change Material Based Battery Thermal Management System,” Energy Conversion and Management 138 (2017): 486–492.

[122] Y. Liu , X. Li , Y. Xu , et al., “Carbon‐Enhanced Hydrated Salt Phase Change Materials for Thermal Management Applications,” Nanomaterials 14 (2024): 1077. 38998682 10.3390/nano14131077 PMC11243696

[123] M. Zhi , R. Fan , L. Zheng , et al., “Experimental Investigation on Hydrated Salt Phase Change Material for Lithium‐Ion Battery Thermal Management and Thermal Runaway Mitigation,” Energy 307 (2024): 132685.

[124] S. Lin , Z. Ling , S. Li , C. Cai , Z. Zhang , and X. Fang , “Mitigation of Lithium‐Ion Battery Thermal Runaway and Inhibition of Thermal Runaway Propagation Using Inorganic Salt Hydrate With Integrated Latent Heat and Thermochemical Storage,” Energy 266 (2023): 126481.

[125] R. Koyama , Y. Arai , Y. Yamauchi , et al., “Thermophysical Properties of Trimethylolethane (TME) Hydrate as Phase Change Material for Cooling Lithium‐Ion Battery in Electric Vehicle,” Journal of Power Sources 427 (2019): 70–76.

[126] T. Quan , Q. Xia , X. Wei , and Y. Zhu , “Recent Development of Thermal Insulating Materials for Li‐Ion Batteries,” Energies 17 (2024): 4412.

[127] J. Xie , J. Li , C. Li , X. Huang , G. Zhang , and X. Yang , “Multi‐Level Passive‐Active Thermal Control for Battery Thermal Runaway Prevention and Suppression in Electric Vehicles,” ETransportation 26 (2025): 100467.

[128] L. D. Tai and M. Y. Lee , “Advances in the Battery Thermal Management Systems of Electric Vehicles for Thermal Runaway Prevention and Suppression,” Batteries 11 (2025): 216.

[129] J. Zhang , L. Zhang , F. Sun , and Z. Wang , “An Overview on Thermal Safety Issues of Lithium‐Ion Batteries for Electric Vehicle Application,” IEEE Access 6 (2018): 23848–23863.

[130] L. Song , Y. Zheng , Z. Xiao , C. Wang , and T. Long , “Review on Thermal Runaway of Lithium‐Ion Batteries for Electric Vehicles,” Journal of Electronic Materials 51 (2021): 30–46.

[131] J. Niu , S. Deng , X. Gao , H. Niu , Y. Fang , and Z. Zhang , “Experimental Study on Low Thermal Conductive and Flame Retardant Phase Change Composite Material for Mitigating Battery Thermal Runaway Propagation,” Journal of Energy Storage 47 (2022): 103557.

[132] Y. Zhou , Y. Jia , J. Zhang , et al., “Fire‐Resistant Polyimide‐Silica Composite Aerogels With High Thermal Insulation and Flame Retardance towards Preventing Thermal Runaway Propagation of Lithium‐Ion Batteries,” Polymer Degradation and Stability 241 (2025): 111574.

[133] S. K. Wong , K. Li , X. Rui , et al., “Mitigating Thermal Runaway Propagation in High Specific Energy Lithium‐Ion Battery Modules Through Nanofiber Aerogel Composite Material,” Energy 307 (2024): 132353.

[134] M. Cui , Y. Zhong , K. Yin , et al., “Synergistic Thermal Management in Lithium‐Ion Batteries: A Phase Change Material‐Silicone Rubber Foam Composite for Suppressing Thermal Runaway Propagation,” Chemical Engineering Journal 525 (2025): 170773.

[135] R. Li , Z. Liu , S. Zheng , et al., “Trifunctional Composite Thermal Barrier Mitigates the Thermal Runaway Propagation of Large‐Format Prismatic Lithium‐Ion Batteries,” Journal of Energy Storage 73 (2023): 109178.

[136] V. Talele , U. Morali , H. Najafi Khaboshan , et al., “Improving Battery Safety by Utilizing Composite Phase Change Material to Delay the Occurrence of Thermal Runaway Event,” International Communications in Heat and Mass Transfer 155 (2024): 107527.

[137] Y. Huang , Y. Fan , L. Sun , et al., “Mechanism of Heat Transfer Suppression and Safety Evaluation of High‐Performance Aerogel Insulation Materials in the Thermal Runaway Propagation of Lithium‐Ion Batteries,” Energy 334 (2025): 137684.

[138] M. Chen , M. Zhu , L. Zhao , and Y. Chen , “Study on Thermal Runaway Propagation Inhibition of Battery Module by Flame‐Retardant Phase Change Material Combined With Aerogel Felt,” Applied Energy 367 (2024): 123394.

[139] Y. Xiao , M. Yan , L. Shi , et al., “High‐Temperature Resistant, Super Elastic Aerogel Sheet Prepared Based on In‐Situ Supercritical Separation Method for Thermal Runaway Prohibition of Lithium‐Ion Batteries,” Energy Storage Materials 61 (2023): 102871.

[140] P. Lyu , G. Chen , X. Liu , M. Li , and Z. Rao , “Mitigating Thermal Runaway Propagation for Lithium‐Ion Batteries by a Novel Integrated Liquid Cooling/Aerogel Strategies,” Applied Thermal Engineering 269 (2025): 126001.

[141] M. Li , M. Niu , Z. Liu , H. Li , and Z. Rao , “Thermal Insulation‐Flame Retardant Synergistic Polyimide Composite Aerogel for Mitigating Thermal Runaway in Lithium‐Ion Batteries,” Applied Thermal Engineering 281 (2025): 128721.

[142] X. Dai , P. Ping , D. Kong , et al., “Heat Transfer Enhanced Inorganic Phase Change Material Compositing Carbon Nanotubes for Battery Thermal Management and Thermal Runaway Propagation Mitigation,” Journal of Energy Chemistry 89 (2024): 226–238.

[143] F. Xiong , J. Zhou , Y. Jin , et al., “Thermal Shock Protection With Scalable Heat‐Absorbing Aerogels,” Nature Communications 15 (2024): 7125. 10.1038/s41467-024-51530-3 PMC11336183 39164288

[144] L. Chen , C. Pereira , S. Pannala , D. Munjurulimana , and H. Goossens , “Mitigation of Cylindrical Lithium Ion Battery Thermal Runaway Propagation With a Flame Retardant Polypropylene Thermal Barrier,” Journal of Energy Storage 108 (2025): 115042.

[145] G. Yin , “Advancements in Insulation Technologies for Electric Vehicle Battery Cells: A Review,” IEEE Transactions on Dielectrics and Electrical Insulation 32 (2025): 2153–2161.

[146] Y. F. Zhang , Y. J. Ren , H. C. Guo , and S. Bai , “Enhanced Thermal Properties of PDMS Composites Containing Vertically Aligned Graphene Tubes,” Applied Thermal Engineering 150 (2019): 840–848.

[147] H. Jung , J. H. Han , and I. C. Jung , “Fabrication of Silicone‐Based Gap Filler for Electric Vehicles Using Magnesium Oxide Thermally Conductive Fillers,” Journal of the Korean Ceramic Society 61 (2024): 713–721.

[148] C. Wang , Y. Wu , and J. Zheng , “Polydimethylsiloxane‐Based Phase Change Composites With Excellent Low‐Temperature Flexibility for Battery Thermal Management,” Materials Today Communications 44 (2025): 111958.

[149] L. Zhou , H. Zhang , Z. Y. Lu , et al., “Preparation of Multifunctional Silicone Rubber Composites With Expanded Graphite‐Encapsulated Phase Change Materials for Thermal Management,” ACS Applied Polymer Materials 7 (2025): 12671–12681.

[150] H. Meng , T. Zhang , Z. Wang , et al., “Experimental Study on a Novel Safety Strategy for Lithium‐Ion Batteries: Fire Suppression and Thermal Runaway Mitigation in Confined Spaces Using Heat‐Triggered Fire‐Extinguishing Polymer Composite (FEPC),” Journal of Energy Storage 130 (2025): 117419.

[151] Y. Huang , X. Shen , Y. Zhao , et al., “Investigation on the Inhibition Mechanism of Thermal Runaway Propagation in High‐Rate Cycling Lithium‐Ion Pouch Cells,” Process Safety and Environmental Protection 197 (2025): 106975.

[152] X. Duan and G. F. Naterer , “Heat Transfer in Phase Change Materials for Thermal Management of Electric Vehicle Battery Modules,” International Journal of Heat and Mass Transfer 53 (2010): 5176–5182.

[153] A. Calborean , L. Máthé , and O. Bruj , “Phase Change Materials for Thermal Management in Lithium‐Ion Battery Packs: A Review,” Batteries 11 (2025): 432.

[154] H. Qiu , Z. Zhang , Z. Ling , and X. Fang , “Developing a Flame‐Retardant Flexible Composite Phase Change Material to Realize both Temperature Control and Thermal Runaway Prevention for Lithium‐Ion Battery Pack,” Applied Thermal Engineering 248 (2024): 123301.

[155] P. Chen , X. Li , C. Li , et al., “The Intrinsic Flame Retardant Multifunctional Composite Phase Change Material With Phosphorus Pentoxide for Battery Thermal Safety System,” Applied Thermal Engineering 256 (2024): 124160.

[156] S. Shivram and H. R , “Impact of Dual Nano‐Enhanced Phase Change Materials on Mitigating Thermal Runaway in Lithium‐Ion Battery Cell,” Case Studies in Thermal Engineering 60 (2024): 104667.

[157] N. Elangkeeran and A. J. Kumar , “A Review on Conductive Composite for Heat Exchange in Electric Vehicle Battery Packs,” Energy Storage 7 (2025): 70291.

[158] X. Liu , P. Su , H. Zhang , et al., “Experimental Study on the Heat Shielding Performance of Multilayer Barriers Incorporating PCM and Aerogel,” Journal of Building Engineering 111 (2025): 113184.

[159] O. E. Akinrinade and A. H. Rosa , “A Global Review of PCBs and Halogenated Flame Retardants in Indoor Air: Implication for Human Exposure Risks,” Chemosphere 384 (2025): 144495. 40499466 10.1016/j.chemosphere.2025.144495

[160] J. de Boer , S. Harrad , and M. Sharkey , “The European Regulatory Strategy for Flame Retardants—The Right Direction but Still a Risk of Getting Lost,” Chemosphere 347 (2024): 140638. 37981017 10.1016/j.chemosphere.2023.140638

[161] S. Tang , L. Qian , X. Liu , and Y. Dong , “Gas‐Phase Flame‐Retardant Effects of a Bi‐Group Compound Based on Phosphaphenanthrene and Triazine‐Trione Groups in Epoxy Resin,” Polymer Degradation and Stability 133 (2016): 350–357.

[162] K. Li , G. Zhang , X. Xu , and B. Yuan , “Engineering an Intrinsic PCM to Balance High Latent Heat and Flame Retardancy for Efficient Lithium‐Ion Battery Thermal Management,” Applied Thermal Engineering 280 (2025): 128294.

[163] X. Li , W. Yang , L. Yin , et al., “Advancing Lithium Battery Safety: Introducing a Composite Phase Change Material With Anti‐Leakage and Fire‐Resistant Properties,” eTransportation 23 (2025): 100387.

[164] W. Miao , R. Quan , J. Ju , et al., “Calcium Chloride Hexahydrate Based Composite Phase Change/Thermochemical Material for Wide‐Temperature Range Passive Battery Thermal Management,” Chemical Engineering Journal 508 (2025): 160800.

[165] W. Guo , J. Yang , L. Li , et al., “Boron Nitride Based Nanostructure Inspired Flame Retarded Thermoplastic Polyurethane Composites: Comprehensive Evaluation and Mechanism Investigation,” Construction and Building Materials 457 (2024): 139478.

[166] P. Chen , T. Wu , Z. Wu , C. Wang , and Z. Kong , “Biomass Aerogel With Humidity Sensitive for Thermal Runaway Suppression of Battery Modules and Flame‐Retardant Application,” Energy 311 (2024): 133170.

[167] Y. Lv , J. Dai , L. Xia , L. Luo , Y. Xu , and L. Dai , “Smoke Suppression and Phosphorus‐Free Condensed Phase Flame‐Retardant Epoxy Resin Composites Based on Salen‐Ni,” Polymer Degradation and Stability 201 (2022): 109980.

[168] J. Ji , Y. An , J. Gu , X. Zhang , C. Zhang , and Z. Shao , “Flexible Composite Phase Change Materials With High Thermal Conductivity and Electrical Insulation Properties for Lithium‐Ion Battery Thermal Management,” Applied Thermal Engineering 266 (2025): 125706.

[169] X. Xu , J. Shen , W. Dong , X. Wang , H. Zhang , and F. Zhou , “Development and Performance Analysis of a Multifunctional Composite Phase Change Cooling Plate for Improving Battery Thermal Management,” Applied Thermal Engineering 266 (2025): 125762.

[170] R. D. McKerracher , J. Guzman‐Guemez , R. G. A. Wills , S. M. Sharkh , and D. Kramer , “Advances in Prevention of Thermal Runaway in Lithium‐Ion Batteries,” Advanced Energy and Sustainability Research 2 (2021): 2000059.

[171] X. Tian , Y. Yi , B. Fang , et al., “Design Strategies of Safe Electrolytes for Preventing Thermal Runaway in Lithium Ion Batteries,” Chemistry of Materials 32 (2020): 9821–9848.

[172] T. Wang , H. Liu , and W. Wang , “Advances in Thermal Management of Lithium‐Ion Batteries: Causes of Thermal Runaway and Mitigation Strategies,” Processes 13 (2025): 2499.

[173] K. Shen , J. Sun , C. Xu , et al., “Experimental Investigation for the Phase Change Material Barrier Area Effect on the Thermal Runaway Propagation Prevention of Cell‐to‐Pack Batteries,” Batteries 9 (2023): 206.

[174] K. Suchorowiec , N. Paprota , and K. Pielichowska , “Aerogels for Phase‐Change Materials in Functional and Multifunctional Composites: A Review,” Materials 17 (2024): 4405. 39274794 10.3390/ma17174405 PMC11396527

[175] J. Feng , Z. Ma , J. Wu , et al., “Fire‐Safe Aerogels and Foams for Thermal Insulation: From Materials to Properties,” Advanced Materials 37 (2025): 2411856. 10.1002/adma.202411856 39558768

[176] C. Jin , Y. Sun , J. Yao , et al., “No Thermal Runaway Propagation Optimization Design of Battery Arrangement for Cell‐to‐Chassis Technology,” eTransportation 14 (2022): 100199.

[177] V. Talele , M. S. Patil , S. Panchal , R. Fraser , and M. Fowler , “Battery Thermal Runaway Propagation Time Delay Strategy Using Phase Change Material Integrated With Pyro Block Lining: Dual Functionality Battery Thermal Design,” Journal of Energy Storage 65 (2023): 107253.

[178] Z. Wang , H. Sadeghi , X. Huang , and F. Restuccia , “Thermal Runaway and Flame Propagation in Battery Packs: Numerical Simulation and Deep Learning Prediction,” Engineering Applications of Computational Fluid Mechanics 19 (2025): 2445160.

[179] C. Liang , X. Lai , K. Shen , et al., “Experimental Study on Immersion Cooling for Delaying Thermal Runaway Propagation in Lithium‑Ion Battery Modules,” Process Safety and Environmental Protection 206 (2026): 108333.

[180] G. Darikas , H. Chen , A. Barai , et al., “Analysis of Internal Cell Temperature Variations Under Different Abuse Test Conditions Using Embedded Temperature Sensors,” Journal of Energy Storage 106 (2025): 114724.

[181] Z. Huang , Y. Yu , Q. Duan , P. Qin , J. Sun , and Q. Wang , “Heating Position Effect on Internal Thermal Runaway Propagation in Large‐Format Lithium Iron Phosphate Battery,” Applied Energy 325 (2022): 119778.

[182] D. Ouyang , Y. Shen , X. Liu , et al., “Investigation on Thermal Runaway Features of Large‐Format Energy Storage Cells Under Overcharge Scenarios,” Applied Thermal Engineering 279 (2025): 128051.

[183] K. Li , Y. Li , W. Shen , et al., “Mitigation Strategy for Li‐Ion Battery Module Thermal Runaway Propagation Triggered by Overcharging,” International Journal of Thermal Sciences 198 (2024): 108880.

[184] Z. Huang , J. Liu , H. Zhai , and Q. Wang , “Experimental Investigation on the Characteristics of Thermal Runaway and Its Propagation of Large‐Format Lithium Ion Batteries Under Overcharging and Overheating Conditions,” Energy 233 (2021): 121103.

[185] A. Mallarapu , N. Sunderlin , V. Boovaragavan , et al., “Effects of Trigger Method on Fire Propagation During the Thermal Runaway Process in Li‐Ion Batteries,” Journal of The Electrochemical Society 171 (2024): 040514.

[186] Y. Zhang , P. Ping , X. Dai , et al., “Failure Mechanism and Thermal Runaway Behavior of Lithium‐Ion Battery Induced by Arc Faults,” Renewable and Sustainable Energy Reviews 207 (2025): 114914.

[187] W. Xu , K. Zhou , H. Wang , et al., “Series Arc‐Induced Internal Short Circuit Leading to Thermal Runaway in Lithium‐Ion Battery,” Energy 308 (2024): 132999.

[188] M. S. Md Said and M. Z. Mohd Tohir , “Visual and Thermal Imaging of Lithium‐Ion Battery Thermal Runaway Induced by Mechanical Impact,” Journal of Loss Prevention in the Process Industries 79 (2022): 104854.

[189] Z. Sun , E. Read , Y. Chen , Y. Dai , J. Marco , and P. R. Shearing , “Numerical and Experimental Characterization of Nail Penetration Induced Thermal Runaway Propagation in 21,700 Lithium‐Ion Batteries: Exploring the Role of Interstitial Thermal Barrier Materials,” Journal of Energy Chemistry 109 (2025): 576–589.

[190] F. Liu , J. Wang , N. Yang , et al., “Experimental Study on the Alleviation of Thermal Runaway Propagation From an Overcharged Lithium‐Ion Battery Module Using Different Thermal Insulation Layers,” Energy 257 (2022): 124768.

[191] D. Chen , J. Jiang , G. H. Kim , C. Yang , and A. Pesaran , “Comparison of Different Cooling Methods for Lithium Ion Battery Cells,” Applied Thermal Engineering 94 (2016): 846–854.

[192] Z. Qian , Y. Li , and Z. Rao , “Thermal Performance of Lithium‐Ion Battery Thermal Management System by Using Mini‐Channel Cooling,” Energy Conversion and Management 126 (2016): 622–631.

[193] F. S. Hwang , T. Confrey , C. Reidy , et al., “Review of Battery Thermal Management Systems in Electric Vehicles,” Renewable and Sustainable Energy Reviews 192 (2024): 114171.

[194] S. Panchal , I. Dincer , M. Agelin‐Chaab , R. Fraser , and M. Fowler , “Experimental and Theoretical Investigations of Heat Generation Rates for a Water Cooled LiFePO4 Battery,” International Journal of Heat and Mass Transfer 101 (2016): 1093–1102.

[195] Y. Xie , S. Shi , J. Tang , H. Wu , and J. Yu , “Experimental and Analytical Study on Heat Generation Characteristics of a Lithium‐Ion Power Battery,” International Journal of Heat and Mass Transfer 122 (2018): 884–894.

[196] T. Rappsilber , N. Yusfi , S. Krüger , et al., “Meta‐Analysis of Heat Release and Smoke Gas Emission During Thermal Runaway of Lithium‐Ion Batteries,” Journal of Energy Storage 60 (2023): 106579.

[197] S. Yang , X. Luo , X. Li , et al., “Comparing Different Battery Thermal Management Systems for Suppressing Thermal Runaway Propagation,” Journal of Energy Storage 101 (2024): 114005.

[198] H. Fu , J. Wang , L. Li , J. Gong , and X. Wang , “Numerical Study of Mini‐Channel Liquid Cooling for Suppressing Thermal Runaway Propagation in a Lithium‐Ion Battery Pack,” Applied Thermal Engineering 234 (2023): 121349.

[199] Y. Ma , Y. Zhang , N. Chen , et al., “Thermal Runaway Propagation Behavior and Cooling Effect of Water Mist Within a 18650‐Type LiFePO_4_ Battery Module Under Different Conditions,” Process Safety and Environmental Protection 185 (2024): 1362–1372.

[200] T. Liu , J. Huang , X. Hu , J. Hu , and X. Wang , “Experimental Study on the Cooling Effect of Fine Water Mist on the Thermal Runaway in a Single Lithium Ion Battery,” Applied Thermal Engineering 240 (2024): 122194.

[201] X. Rui , X. Feng , H. Wang , et al., “Synergistic Effect of Insulation and Liquid Cooling on Mitigating the Thermal Runaway Propagation in Lithium‐Ion Battery Module,” Applied Thermal Engineering 199 (2021): 117521.

[202] X. Liu , Z. Zhou , W.‐T. Wu , et al., “Inhibition of Thermal Runaway Propagation in Lithium‐Ion Battery Pack by Minichannel Cold Plates and Insulation Layers,” International Journal of Energy Research 2023 (2023): 1–29.

[203] Y. Chen , X. Guo , C. Shi , X. Zhou , and D. Zou , “Preparation of Metal‐Based Microencapsulated Phase Change Material and Its Application in a Battery for Thermal Management and Thermal Runaway Protection,” Composites Part B: Engineering 298 (2025): 112376.

[204] Y. Wang , Y. Wang , T. He , and N. Mao , “A Numerical Study on a Hybrid Battery Thermal Management System Based on PCM and Wavy Microchannel Liquid Cooling,” Renewable Energy 235 (2024): 121273.

[205] T. Ouyang , B. Liu , C. Wang , J. Ye , and S. Liu , “Novel Hybrid Thermal Management System for Preventing Li‐Ion Battery Thermal Runaway Using Nanofluids Cooling,” International Journal of Heat and Mass Transfer 201 (2023): 123652.

[206] X. Gu , W. Lei , J. Xi , and M. Song , “Investigation and Optimization of Battery Thermal Management System Based on Composite Phase Change Material and Variable Wall Liquid Cooling Plate,” International Journal of Thermofluids 24 (2024): 100886.

[207] R. Jian , P. Wang , W. Duan , et al., “Synthesis of a Novel P/N/S‐Containing Flame Retardant and Its Application in Epoxy Resin: Thermal Property, Flame Retardance, and Pyrolysis Behavior,” Industrial & Engineering Chemistry Research 55 (2016): 11520–11527.

[208] H. Lin , Z. Yao , G. Zhang , and X. Yang , “Dual‐Functional Flame‐Retardant Composite Phase Change Material for Thermal Management and Thermal Runaway Suppression,” ACS Applied Energy Materials 8 (2025): 10707–10716.

[209] M. E. Shabestari , E. N. Kalali , V. J. González , et al., “Effect of Nitrogen and Oxygen Doped Carbon Nanotubes on Flammability of Epoxy Nanocomposites,” Carbon 121 (2017): 193–200.

[210] Y. Chen , C. Tian , Y. Tu , et al., “Paraffin/Graphite/Boron Nitride Composite as a Novel Phase Change Material for Rapid Heat Absorption in Battery Thermal Management Technology,” International Journal of Heat and Mass Transfer 235 (2024): 126214.

[211] V. Dananjaya , X. Bao , N. Hansika , and C. Abeykoon , “Structural and Thermal Properties of Paraffin‐Based Graphene and Carbon Fibre Composite Phase Change Materials,” International Journal of Heat and Mass Transfer 255 (2026): 127696.

[212] M. Chen , M. Zhu , S. Zhang , et al., “Experimental Investigation on Mitigation of Thermal Runaway Propagation of Lithium‐Ion Battery Module With Flame Retardant Phase Change Materials,” Applied Thermal Engineering 235 (2023): 121401.

[213] H. Vothi , C. Kim , T. Nguyen , J. Lee , L.‐A. T. Nguyen , and J. Suhr , “Thermal Degradation and Flame Retardancy of Nylon 6/Aluminum Methylmethoxy Phosphonate Composites,” RSC Advances 13 (2023): 5219–5227. 36777944 10.1039/d2ra07297a PMC9910282

[214] T. Wang , J. Deng , J. Du , et al., “Investigation on the Polyethylene Glycol Based Composite Phase Change Materials With Coating Flame‐Retardant for Battery Thermal Management,” Case Studies in Thermal Engineering 65 (2025): 105616.

[215] J. Wang , C. Ma , X. Mu , et al., “Construction of Multifunctional MoSe2 Hybrid towards the Simultaneous Improvements in Fire Safety and Mechanical Property of Polymer,” Journal of Hazardous Materials 352 (2018): 36–46. 29571027 10.1016/j.jhazmat.2018.03.003

[216] F. Wang , Y. Tao , Z. Xu , et al., “Construction of a Green Flame‐Retardant Shield for Thermoplastic Polyurethane Composites via Interactive Effects of Nickel–Cobalt–Manganese and Ammonium Polyphosphate: From Waste Batteries to Flame Retardants,” Langmuir 41 (2025): 33869–33879. 41378454 10.1021/acs.langmuir.5c04210

[217] X. Liu , K. A. Salmeia , D. Rentsch , J. Hao , and S. Gaan , “Thermal Decomposition and Flammability of Rigid PU Foams Containing some DOPO Derivatives and Other Phosphorus Compounds,” Journal of Analytical and Applied Pyrolysis 124 (2017): 219–229.

[218] P. J. Elliot and R. H. Whiteley , “A Cone Calorimeter Test for the Measurement of Flammability Properties of Insulated Wire,” Polymer Degradation and Stability 64 (1999): 577–584.

[219] Y. Zhou , Y. Liu , H. Zhang , et al., “Aloe Vera‐Inspired Boron Nitride/Expanded Graphite/Melamine Phosphate Polymer Composites for Battery Temperature Management,” Energy 336 (2025): 138447.

[220] M. G. Rasul , A. Kiziltas , C. D. Malliakas , et al., “Polyethylene‐BN Nanosheets Nanocomposites With Enhanced Thermal and Mechanical Properties,” Composites Science and Technology 204 (2021): 108631.

[221] R. Thandavamoorthy , Y. Devarajan , J. B. Pampania , et al., “Development of Carbon Fiber Reinforced Niobium Particulate Epoxy Composites for High‐Performance Structural and Thermal Management Applications,” Journal of Materials Research and Technology 42 (2026): 4341–4354.

[222] compass . https://compass.astm.org/content‐access?contentCode=ASTM%7CD0790‐17%7Cen‐US.

[223] Y. Yue , J. Wang , L. He , et al., “Thermal Runaway Propagation Suppression Efficacy of Various Thermal Insulation Materials towards Large‐Capacity Battery: Performance Evaluation and Mechanism Investigation,” Applied Thermal Engineering 296 (2026): 130571.

[224] H. Xiao , J. E , S. Tian , Y. Huang , and X. Song , “Effect of Composite Cooling Strategy Including Phase Change Material and Liquid Cooling on the Thermal Safety Performance of a Lithium‐Ion Battery Pack Under Thermal Runaway Propagation,” Energy 295 (2024): 131093.

[225] D. Bernardi , E. Pawlikowski , and J. Newman , “General Energy Balance for Battery Systems,” Electrochemical Society Extended Abstracts 84–2 (1984): 164–165.

[226] D. Ren , X. Liu , X. Feng , et al., “Model‐Based Thermal Runaway Prediction of Lithium‐Ion Batteries From Kinetics Analysis of Cell Components,” Applied Energy 228 (2018): 633–644.

[227] R. W. Kennedy , S. Bilyaz , and O. A. Ezekoye , “Low‐Order Modeling of Lithium Cobalt Oxide Lithium Ion Battery Arrays With Various States of Charge,” Journal of Energy Storage 49 (2022): 104053.

[228] J. Chen , X. Rui , H. Hsu , et al., “Thermal Runaway Modeling of LiNi_0.6_Mn_0.2_Co_0.2_O_2_/Graphite Batteries Under Different States of Charge,” Journal of Energy Storage 49 (2022): 104090.

[229] P. Zhao , L. Liu , Y. Chen , and H. Ge , “Theoretical and Numerical Analysis for Thermal Runaway Propagation Within a Single Cell,” International Journal of Heat and Mass Transfer 181 (2021): 121901.

[230] F. Zhang , X. Feng , C. Xu , F. Jiang , and M. Ouyang , “Thermal Runaway front in Failure Propagation of Long‐Shape Lithium‐Ion Battery,” International Journal of Heat and Mass Transfer 182 (2022): 121928.

[231] Y. Jia , P. Zhao , D. P. Finegan , and J. Xu , “Dynamics of Intra‐Cell Thermal Front Propagation in Lithium‐Ion Battery Safety Issues,” Advanced Energy Materials 14 (2024): 2400621.

[232] Z. Y. Jiang , Z. G. Qu , J. F. Zhang , and Z. H. Rao , “Rapid Prediction Method for Thermal Runaway Propagation in Battery Pack Based on Lumped Thermal Resistance Network and Electric Circuit Analogy,” Applied Energy 268 (2020): 115007.

[233] J. Chen , D. Ren , H. Hsu , et al., “Investigating the Thermal Runaway Features of Lithium‐Ion Batteries Using a Thermal Resistance Network Model,” Applied Energy 295 (2021): 117038.

[234] C. X. He , Y. H. Liu , X. Y. Huang , et al., “A Reduced‐Order Thermal Runaway Network Model for Predicting Thermal Propagation of Lithium‐Ion Batteries in Large‐Scale Power Systems,” Applied Energy 373 (2024): 123955.

[235] Y. Xia , Y. Hong , Z. Zhu , et al., “Predicting Thermal Runaway Propagation in Lithium‐Ion Batteries: An Experimental and Theoretical Study,” Applied Thermal Engineering 279 (2025): 127780.

[236] G. Wang , D. Kong , P. Ping , et al., “Modeling Venting Behavior of Lithium‐Ion Batteries During Thermal Runaway Propagation by Coupling CFD and Thermal Resistance Network,” Applied Energy 334 (2023): 120660.

[237] G. Wang , W. Gao , X. He , et al., “Numerical Investigation on Thermal Runaway Propagation and Prevention in Cell‐to‐Chassis Lithium‐Ion Battery System,” Applied Thermal Engineering 236 (2024): 121528.

[238] Y. Wu , A. C. Y. Yuen , C. Mo , and X. Huang , “Modelling and Optimization of a Thermal Management and Barrier Integration Structure by Coupling CFD and Reduced‐Order Thermal Resistance Network,” Energy Conversion and Management 343 (2025): 120188.

[239] Y. Wang , X. Feng , D. Guo , et al., “Temperature Excavation to Boost Machine Learning Battery Thermochemical Predictions,” Joule 8 (2024): 2639–2651.

[240] N. Ouyang , W. Zhang , X. Yin , et al., “A Data‐Driven Method for Predicting Thermal Runaway Propagation of Battery Modules Considering Uncertain Conditions,” Energy 273 (2023): 127168.

[241] H. Kitagawa , Y. Takagishi , M. Nishiuchi , et al., “Modelling of Thermal Runaway Propagation in Li‐Ion Battery Cells Considering Variations in Thermal Property Measurements,” Batteries 11 (2025): 386.

[242] C. Xu , H. Wang , F. Jiang , et al., “Modelling of Thermal Runaway Propagation in Lithium‐Ion Battery Pack Using Reduced‐Order Model,” Energy 268 (2023): 126646.

[243] X. Zhang , J. Yao , L. Zhu , et al., “Experimental and Simulation Investigation of Thermal Runaway Propagation in Lithium‐Ion Battery Pack Systems,” Journal of Energy Storage 77 (2024): 109868.

[244] Y. Zhang , L. Song , J. Tian , et al., “Modeling the Propagation of Internal Thermal Runaway in Lithium‐Ion Battery,” Applied Energy 362 (2024): 123004.

[245] J. T. Kim , J. Y. Choi , S. Kang , N. G. Han , and D. K. Kim , “Development of Thermal Runaway Propagation Model Considering Vent Gas Combustion for Electric Vehicles,” Journal of Energy Storage 60 (2023): 106535.

[246] T. Zhang , X. Qiu , M. Li , et al., “Thermal Runaway Propagation Characteristics and Preventing Strategies Under Dynamic Thermal Transfer Conditions for Lithium‐Ion Battery Modules,” Journal of Energy Storage 58 (2023): 106463.

[247] Y. Yang , D. Raymand , W. Guo , and D. Brandell , “Modeling the Interplay Between Aging and Thermal Runaway Propagation in Large‐Format Lithium‐Ion Batteries,” Journal of Power Sources Advances 38 (2026): 100203.

[248] Y. S. Choi , S. H. Lee , J. Hong , and J. Park , “Experimental and Numerical Studies on the Thermomechanical Deformation of Lithium‐Ion Battery Pack Housing Under Thermal Runaway Propagation Condition,” eTransportation 25 (2025): 100431.

[249] E. Kwak , J. Jeong , J.‐H. Kim , and K. Y. Oh , “A Rapid Multiphysics Framework for Predicting Thermal Runaway Propagation in Lithium‐Ion Battery Packs,” eTransportation 28 (2026): 100566.

[250] W. Zhang , N. Ouyang , X. Yin , X. Li , W. Wu , and L. Huang , “Data‐Driven Early Warning Strategy for Thermal Runaway Propagation in Lithium‐Ion Battery Modules With Variable state of Charge,” Applied Energy 323 (2022): 119614.

[251] S. Chen , X. Wei , Z. Zhu , et al., “Thermal Runaway front Propagation Characteristics, Modeling and Judging Criteria for Multi‐Jelly Roll Prismatic Lithium‐Ion Battery Applications,” Renewable Energy 231 (2024): 121045.

[252] W. Tan , L.‐B. Ren , T. Tian , K. Ma , S.‐L. Wang , and Z.‐Y. Zhang , “Thermal Runaway Propagation and Suppression in Mobile Energy Storage Lithium Battery Module,” Journal of Energy Storage 131 (2025): 117615.

[253] X. Feng , F. Zhang , W. Huang , Y. Peng , C. Xu , and M. Ouyang , “Mechanism of Internal Thermal Runaway Propagation in Blade Batteries,” Journal of Energy Chemistry 89 (2024): 184–194.

[254] D. Mishra , P. Zhao , and A. Jain , “Thermal Runaway Propagation in Li‐Ion Battery Packs due to Combustion of Vent Gases,” Journal of The Electrochemical Society 169 (2022): 100520.

[255] D. Mishra , R. Tummala , and A. Jain , “Investigation of Propagation of Thermal Runaway During Large‐Scale Storage and Transportation of Li‐Ion Batteries,” Journal of Energy Storage 72 (2023): 108315.

[256] W. Zhang , J. Yuan , J. Huang , and Y. Xie , “Uncertainty Assessment Method for Thermal Runaway Propagation of Lithium‐Ion Battery Pack,” Applied Thermal Engineering 238 (2024): 121946.

[257] J. Yu , C. Guo , and J. Yu , “A Novel Methodology for Modeling and Analyzing Thermal Runaway Propagation in Lithium‐Ion Battery Modules Using Probability Functions,” Applied Thermal Engineering 257 (2024): 124503.

[258] A. Karmakar , H. Zhou , B. S. Vishnugopi , and P. P. Mukherjee , “Thermal Runaway Propagation Analytics and Crosstalk in Lithium‐Ion Battery Modules,” Energy Technology 12 (2024): 2300707.

[259] J. Gong , B. Liu , H. Lian , et al., “Numerical Investigation of Suppressing Thermal Runaway Propagation in a Lithium‐Ion Battery Pack Using Thermal Insulators,” Process Safety and Environmental Protection 176 (2023): 1063–1075.

[260] W. Yan , Z. Wang , D. Ouyang , and S. Chen , “Analysis and Prediction of Thermal Runaway Propagation Interval in Confined Space Based on Response Surface Methodology and Artificial Neural Network,” Journal of Energy Storage 55 (2022): 105822.

[261] J. B. Goodenough , “A Review of Thermal Runaway Prevention and Mitigation Strategies for Lithium‐Ion Batteries,” Energy Conversion and Management: X 16 (2022): 100310.

[262] T. He , S. Gadkari , T. Zhang , et al., “Investigation of the Internal Physical and Chemical Changes of a Cylindrical Lithium‐Ion Battery During Thermal Runaway,” Journal of Cleaner Production 434 (2024): 140548.

[263] H. Zhang , J. Xue , Y. Qin , et al., “Full‐Dimensional Analysis of Gaseous Products to Unlocking In Depth Thermal Runaway Mechanism of Li‐Ion Batteries,” Small 20 (2024): 2406110. 10.1002/smll.202406110 39113670

[264] Y. Liu , Z. Liu , W. Mei , et al., “Operando Monitoring Lithium‐Ion Battery Temperature via Implanting Femtosecond‐Laser‐Inscribed Optical Fiber Sensors,” Measurement 203 (2022): 111961.

[265] B. Li , M. H. Parekh , R. A. Adams , et al., “Lithium‐Ion Battery Thermal Safety by Early Internal Detection, Prediction and Prevention,” Scientific Reports 9 (2019): 13255. 31519993 10.1038/s41598-019-49616-w PMC6744460

[266] J. Fan , C. Liu , N. Li , et al., “Wireless Transmission of Internal Hazard Signals in Li‐Ion Batteries,” Nature 641 (2025): 639–645. 40369137 10.1038/s41586-025-08785-7

[267] R. Y. Yu , B. C. Wang , and Y. Wang , “On‐Board Implementation of Thermal Runaway Detection in Lithium‐Ion Battery Packs: Methods, Metrics, and Challenges,” Energies 19 (2026): 858.

[268] W. Mei , Z. Liu , C. Wang , et al., “Operando Monitoring of Thermal Runaway in Commercial Lithium‐Ion Cells via Advanced Lab‐on‐Fiber Technologies,” Nature Communications 14 (2023): 5251. 10.1038/s41467-023-40995-3 PMC10462619 37640698

[269] W. C. Tam , J. Chen , H. Fang , W. Tang , J. Deng , and A. Putorti , “Development of an Early‐Stage Thermal Runaway Detection Model for Lithium‐Ion Batteries,” Journal of Power Sources 641 (2025): 236714.

[270] DOE CODE , Project Metadata for Code ID 176833, https://www.osti.gov/doecode/biblio/176833.

[271] EverBatt , A Closed‐Loop Battery Recycling Cost and Environmental Impacts Model Energy Systems Division, www.anl.gov.

[272] J. Ruppert , P. Voß , L. Ihlbrock , J. Palm , S. Lux , and J. Leker , “Analyzing Material and Production Costs for Lithium‐Ion and Sodium‐Ion Batteries Using Process‐Based Cost Modeling—CellEst 3.0,” Journal of Power Sources Advances 36 (2025): 100190.

[273] M. Greenwood , M. Wentker , and J. Leker , “A Bottom‐Up Performance and Cost Assessment of Lithium‐Ion Battery Pouch Cells Utilizing Nickel‐Rich Cathode Active Materials and Silicon‐Graphite Composite Anodes,” Journal of Power Sources Advances 9 (2021): 100055.

[274] V. Pechancová , L. Hrbacková , H. Ersoy , et al., “From Minerals to Dollars: A Scrutiny of Lithium‐Ion Battery Cost Models,” Sustainable Futures 11 (2026): 101767.

